# Evaluation of Domains of Patient-Reported Outcome Measures for Recovery After Childbirth: A Scoping and Systematic Review

**DOI:** 10.1001/jamanetworkopen.2020.5540

**Published:** 2020-05-22

**Authors:** Pervez Sultan, Nishant Sadana, Nadir Sharawi, Lindsay Blake, Kariem El-Boghdadly, Andrea Falvo, Sarah Ciechanowicz, Waseem Athar, Raj Shah, Nan Guo, Sally Jensen, Yasser El-Sayed, David Cella, Brendan Carvalho

**Affiliations:** 1 Stanford University School of Medicine, Stanford, California; 2 Department of Anesthesia, Mercyhealth, Rockford, Illinois; 3 Department of Anesthesia, University of Arkansas for Medical Sciences, Little Rock; 4 Anaesthetic Service, Guy’s and St Thomas National Health Service Foundation Trust, London, United Kingdom; 5 The Anaesthetic Department, King’s College London, London, United Kingdom; 6 Anaesthetics Department, University College London Hospital, London, United Kingdom; 7 Department of Anaesthesia, Watford General Hospital, Watford, United Kingdom; 8 Department of Medical Social Sciences, Feinberg School of Medicine, Northwestern University, Chicago, Illinois; 9 Department of Obstetrics and Gynecology, Stanford University School of Medicine, Stanford, California

## Abstract

**Importance:**

Despite the global delivery rate being approximately 259 deliveries per minute in 2018, postpartum recovery remains poorly defined.

**Objectives:**

To identify validated patient-reported outcome measures (PROMs) used to assess outpatient and inpatient postpartum recovery, evaluate frequency of PROM use, report the proportion of identified PROMs used within each recovery domain, report the number of published studies within each recovery domain, summarize descriptive data (country of origin, year of study, and journal specialty) for published studies using PROMs to evaluate postpartum recovery, and report PROMs used to evaluate global postpartum recovery.

**Evidence Review:**

This study followed PRISMA-ScR guidelines. A literature search of 4 databases (MEDLINE through PubMed, Embase, Web of Science, and CINAHL) was performed on July 1, 2019, to identify PROMs used to evaluate 12 author-defined domains of postpartum recovery. All psychometrically evaluated PROMs used to evaluate inpatient or outpatient postpartum recovery after all delivery modes were included.

**Findings:**

From 8008 screened titles and abstracts, 573 studies (515 outpatient and 58 inpatient) were identified in this review. A total of 201 PROMs were used to assess recovery for outpatient studies and 73 PROMs were used to assess recovery for inpatient studies. The top 5 domains (with highest to lowest numbers of PROMs) used to assess outpatient recovery were psychosocial distress (77 PROMs), surgical complications (26 PROMs), psychosocial support (27 PROMs), motherhood experience (16 PROMs), and sexual function (13 PROMs). Among inpatient studies, the top 5 domains were psychosocial distress (32 PROMs), motherhood experience (7 PROMs), psychosocial support (5 PROMs), fatigue (5 PROMs), and cognition (3 PROMs). The 3 most frequently used PROMs were the Edinburgh Postnatal Depression Scale (267 studies), Short-Form 36 Health Questionnaire (global recovery assessment; 40 studies), and Female Sexual Function Index (35 studies). A total of 24 global recovery PROMs were identified among all included studies. Most studies were undertaken in the United States within the last decade and were published in psychiatry and obstetrics and gynecology journals.

**Conclusions and Relevance:**

Most PROMs identified in this review evaluated a single domain of recovery. Future research should focus on determining the psychometric properties of individual and global recovery PROMs identified in this review to provide recommendations regarding optimum measures of postpartum recovery.

## Key Points

**Question:**

Which patient-reported outcome measures have been used to evaluate global and individual domains of postpartum recovery?

**Findings:**

In this systematic review, 573 studies were included that used 233 patient-reported outcome measures (201 specific to outpatient studies) to assess postpartum recovery.

**Meaning:**

Patient-reported outcome measure use among studies assessing postpartum recovery is heterogeneous, which highlights the need to psychometrically evaluate the quality of available patient-reported outcome measures to formulate recommendations regarding which instruments to use.

## Teaser

This systematic review identifies patient-reported outcome measures (PROMs) used to evaluate global and individual domains of postpartum recovery.

## Introduction

In 2018, the world birth rate was approximately 259 deliveries per minute.^1^ Peripartum care is therefore responsible for a significant percentage of global health care expenditures. Recommendations regarding obstetric enhanced recovery have thus far focused on antepartum and inpatient postpartum care after cesarean delivery.^2,3,4,5^ Inpatient and outpatient postpartum recovery remain poorly defined. Approximately 10% of women undergoing cesarean delivery do not recover (defined by pain resolution, cessation of opioids, and self-assessed functional recovery) by day 50 postpartum.^6^ Poor postpartum recovery can affect families, health care systems, society, and decisions made regarding future childbirth.

Patient-reported outcome measures (PROMs) are structured questionnaires allowing patients to report their health status. The Quality of Recovery (QoR)–40 and QoR-15 are examples of clinically useful PROMs, which accurately measure nonobstetric postoperative quality of recovery^7,8^ and correlate with surgery duration and complexity.^9,10^ Value-based reimbursements based on perioperative PROM data have also been introduced into health care systems such as the National Health Service.^11^ To our knowledge, few PROMs have been developed to assess global inpatient and outpatient postpartum recovery. This may be partly because after hospital discharge, focus rapidly shifts from maternal well-being to neonatal feeding and development, in addition to recovery being difficult to define and multifactorial.

A systematic review (involving authors from this review) concluded that the Obstetric Quality of Recovery–11 scoring tool (ObsQoR-11) was the best PROM to assess functional recovery after cesarean delivery, as assessed by measures of validity, reliability, and responsiveness.^12^ However, this tool has only been validated for use up to 24 hours after delivery and did not include measures of psychological recovery.

The aims of this scoping review were to identify PROMs used to evaluate outpatient and inpatient recovery after childbirth, evaluate the frequency of PROM use, summarize descriptive data of included studies (year, country of publication, and journal specialty), and identify global recovery PROMs (most commonly, used, those developed for use in postpartum populations, and the PROM covering the greatest number of outpatient recovery domains).

## Methods

A medical librarian (L.B.) performed a literature search with no language restriction and without the use of date limiters on July 1, 2019, using the MEDLINE through PubMed, Embase, Web of Science, and CINAHL databases. Subjective PROMs of recovery after childbirth via all delivery modes were sought. The search strategy was composed by reviewing the 12 recovery domains proposed by Sharawi et al^12^ and matching them with all possible available subject headings and key words. Searches were created for each domain and reviewed by the group to supplement any missing ideas or key words. Individual domain searches were then combined into the larger search. The search strategy included terms and alternative spellings related to cesarean delivery, spontaneous vaginal delivery, and assisted vaginal delivery, in addition to evaluation methods and recovery of function. A detailed search strategy is provided in eMethods 1 in the Supplement. We developed a final list of 12 outpatient-specific postpartum recovery domains (excluding global recovery) and 8 subdomains for all modes of childbirth (eMethods 2 in the Supplement). After discussion among authors and after review of included abstracts, agreement was reached regarding the final list of domains used to describe the construct of outpatient recovery. This scoping review followed the Preferred Reporting Items for Systematic reviews and Meta-Analyses Extension for Scoping Reviews (PRISMA-ScR) guidelines.^13^

PROMs were included if they had been psychometrically evaluated (validated) in either an obstetric or nonobstetric study population. A PROM was considered to be validated if the study itself or another published article reported at least 1 measure of validity, reliability, or responsiveness as described by the COSMIN (Consensus‐Based Standards for the Selection of Health Measurement Instruments) group.^14^ Three authors (P.S., N. Sadana, and N. Sharawi) evaluated validation status of reviewed PROMs. We included all study designs including PROMs mentioned in review articles. If a PROM included more than 3 recovery domains assessing elements of global recovery (global health state) rather than recovery associated with a specific domain, it was termed a global recovery measure. The EuroQoL (EQ-5D-3L) PROM, for example, measures global health status through 5 domains and is therefore a global recovery measure. However, the Oswestry Disability Index is a PROM assessing “pain” because it specifically assesses the association of pain with several domains (physical function, sleep, social, and pain), rather than how these domains are associated with the patient’s overall global health state. Absence of evidence of a validation process (ie, ad hoc instruments) resulted in exclusion of the PROM. We excluded PROMs evaluating satisfaction, patient experience, and measures of antenatal, labor, or predelivery experience, and excluded objective health care worker–assessed measures of recovery such as the Bromage Motor Blockade score,^15^ Ramsey Sedation scale score,^16^ and LATCH (how well the infant latches onto the breast, amount of audible swallowing noted, mother’s nipple type, mother’s level of comfort, and amount of help the mother needs to hold her infant to the breast) score.^17^

### Data Collection

After removal of duplicates and animal studies, articles were entered into the Rayyan reviewing system online.^18^ All abstracts were reviewed by a minimum of 2 of us. Any disagreements were discussed among 4 authors (P.S., N. Sadana, N. Sharawi, and B.C.) until all team members agreed. Because of the volume of studies we anticipated that would require screening, we elected to include studies only if the validated PROM name was explicitly mentioned in the article title or within the abstract of a fully published article. Outpatient studies required reference to an outpatient, community or clinic (rather than hospital, ward, or inpatient) setting, or the reporting of PROM time points beyond 5 days postpartum. A standardized data collection tool was used by 8 authors (P.S., N. Sadana, N. Sharawi, L.B., K.E., A.F., W.A., and R.S.) to extract PROM data from the included studies. PROMs were assigned to individual domains by 3 authors (P.S., N. Sadana, and N. Sharawi). Further opinion was obtained when necessary from 1 additional author (B.C.). Per PRISMA-ScR guidance, evaluation of quality of evidence of included studies was outside the scope of this review. Studies containing a PROM and meeting the above inclusion criteria had the following data extracted: year the study was published, country of the study, journal specialty type, and PROM used to assess inpatient or outpatient (or both) recovery. Relevant extracted data were entered into an Excel spreadsheet and graphs were made using Microsoft Excel,version 14.7.7 (Microsoft Corp).

### Outcome Measures

The outcome measures included identification of validated outpatient and inpatient recovery scoring tools after all modes of delivery, frequency of PROM use among included studies, proportion of identified PROMs within each domain that were used for inpatient and outpatient assessment of recovery, descriptive data regarding published studies (year, country of publication, and journal specialty), and identification of global recovery PROMs for outpatient and inpatient studies. For the identified global postpartum recovery PROMs that were developed and validated for use in this setting, we also sought to evalulate the frequency of their use and the number of domains evaluated by each PROM.

## Results

**Figure 1.**
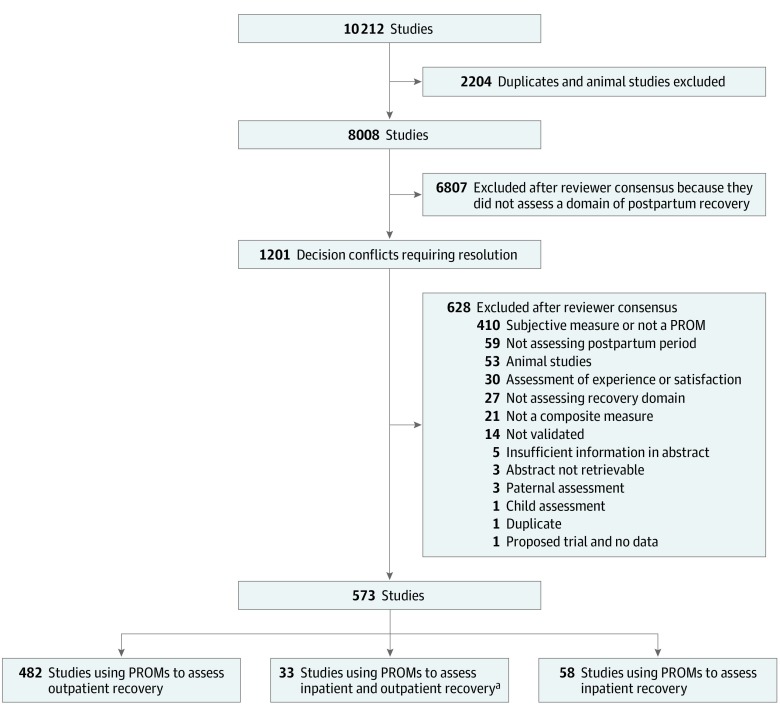
Summary of Scoping Review Search ^a^In these included studies, the same patient-reported outcome measures (PROMs) were used to assess both inpatient and outpatient recovery.

The literature search identified 10 212 publications, reduced to 8008 after removal of duplicates and animal studies. The summary of the search is provided in Figure 1. Of the 573 included studies, 515 studies used PROMs assessing some aspect of outpatient recovery and 58 studies used PROMs assessing only inpatient recovery. A total of 233 PROMs were used in the 573 included articles. The most frequently used PROMs are summarized in eTable 1 in the Supplement.

### Outpatient Recovery PROMs

Among the 515 studies that evaluated outpatient recovery (eResults in the Supplement), 482 evaluated outpatient recovery only and 33 evaluated inpatient and outpatient recovery. A total of 201 PROMs (including global recovery PROMs) were used in these 515 studies (eTable 2 in the Supplement).

### Inpatient Recovery PROMs

The 58 studies evaluating inpatient recovery used 73 different PROMs, which are summarized in eTable 3 in the Supplement. Of the 73 inpatient recovery PROMs, 41 were also used to assess outpatient recovery. Therefore 32 unique inpatient recovery PROMs were identified (eTable 3 in the Supplement).

### Number of PROMs Within Each Domain

The domains with the highest to lowest numbers of PROMs used to assess outpatient recovery were psychosocial distress (77), surgical complications (26), psychosocial support (27), motherhood experience (16), sexual function (13), pain (8), sleep (7), fatigue (5), physical function (2), breastfeeding and breast health (2), scar and wound healing (1), and cognition (0). Furthermore, 6 domains (physical function, surgical complications, pain, fatigue, scar and wound healing, and cognition) lacked PROMs developed for specific use to assess recovery in the postpartum outpatient population. The domains with the highest to lowest numbers of PROMs used to evaluate inpatient recovery domains were psychosocial distress (32), motherhood experience (7), psychosocial support (5), fatigue (5), cognition (3), breastfeeding and breast health (2), pain (2), physical function (2), sexual function (1), sleep (1), scar and wound healing (0), and surgical complications (0).

### Proportion of Studies Using PROMs From Each Domain

The 3 most frequently used PROMs were the Edinburgh Postnatal Depression Scale (267 studies), Short-Form 36 Health Questionnaire (global recovery assessment; 40 studies), and Female Sexual Function Index (35 studies). The numbers of outpatient studies (and proportion of studies from each domain excluding global PROMs) using PROMs within each domain are reported in Table 1 and the numbers of inpatient studies using PROMs within each domain are reported in Table 2. Fifty-seven percent of all studies used PROMs assessing psychosocial distress. The highest numbers of outpatient studies using PROMs were from domains of psychosocial distress, surgical complications, and sexual function. The highest numbers of inpatient studies using PROMs were from domains of psychosocial distress, pain, and motherhood experience. The most commonly used PROMs in the psychosocial distress subdomains (depression, anxiety, and psychological) for inpatient and outpatient studies were the Edinburgh Postnatal Depression Scale,^19^ the State Trait Anxiety Inventory,^20^ and the Impact of Event scale.^21^

**Table 1.** PROMs Used to Evaluate Outpatient Recovery After Childbirth According to Domains^a^

Domain	PROMs, No.	Studies, No.	Proportion of studies evaluating each domain, %	Most commonly used PROMs (No. of studies)
Physical function	2	2	<1	Disability Rating Index (1)
				KATZ-ADL (1)
Surgical complications				
Urology	17	72	16	ICIQ-UI SF (16)
Gynecology	7	16		ICIQ-VS (3)
Colorectal	11	47		Wexner Scale (8)
Pain	8	19	2	McGill Pain Score (9)
Psychosocial distress				
Psychological	50	115	63	Impact of Event Scale (21)
Anxiety	11	53		STAI (19)
Depression	25	362		EPDS (231)
Psychosocial support	27	40	5	Personal Resource Questionnaire (6)
Sleep	7	13	1	Pittsburgh Sleep Quality Index (5)
Motherhood experience				
Adapting to maternal role	11	21	4	Parenting Sense of Competence Scale-Efficacy Sub-scale (6)
Maternal-neonatal bonding	5	13		Mother-Infant Bonding Scale (5)
Breastfeeding and breast health	2	7	<1	Breastfeeding Self-Efficacy Scale (6)
Fatigue	5	10	1	Modified Fatigue Symptom Checklist (3)
Sexual function	13	56	7	FSFI (34)
Scar and wound healing	1	1	<1	Patient Scar Assessment Scale (1)
Global	17	88	NA	SF-36 (32)

Abbreviations: EPDS, Edinburgh Postnatal Depression Score; FSFI, Female Sexual Function Index; ICIQ-UI SF, International Consultation on Incontinence Questionnaire–Urinary Incontinence-Short Form; ICIQ-VS, International Consultation on Incontinence Questionnaire–Vaginal Symptoms; KATZ-ADL, Katz Index of Independence in Activities of Daily Living; NA, not applicable; PROMs, patient-reported outcome measures; SF-36, Short-Form 36 Health Survey; STAI, State-Trait Anxiety Inventory.

^a^ A total of 201 outpatient-specific PROMs were identified among included studies; no PROMs assessing cognition domain were among the included outpatient studies. Many studies used several PROMs.

**Table 2.** PROMs Used to Evaluate Inpatient Recovery After Childbirth According to Domains

**Domain**	**PROMs, No.**	**Studies, No.**	**Studies in each domain, %**	**Most commonly used PROMs (No. of studies)**
Physical function	2	2	1	Rhodes Index of Nausea and Vomiting (1)^a^
Pain	2	9	7	McGill Pain Scale (8)
Psychosocial distress				
Psychological	19	25	72	Impact of Event Scale (3)
Anxiety	3	15		STAI (12)
Depression	11	58		EPDS (36)
Psychosocial support	5	6	4	Berlin Social Support Scale (2)
Sleep	1	1	<1	Athens Insomnia Scale (1)^a^
Motherhood experience				
Adapting to maternal role	2	2	6	Parenting Needs and Parenting Confidence Questionnaire (1)^a^
Maternal-neonatal bonding	5	6		Postpartum Bonding Questionnaire (2)
Breastfeeding and breast health	2	3	2	Breastfeeding Self-Efficacy Scale (2)
Fatigue	5	6	5	Fatigue Continuum Form (2)^a^
Sexual function	1	1	<1	FSFI (1)
Cognition	3	3	2	Attentional Function Index (1)^a^
Global	13	23	NA	SF-36 (8)

Abbreviations: EPDS, Edinburgh Postnatal Depression Score; FSFI, Female Sexual Function Index; NA, not applicable; PROMs, Patient-Reported Outcome Measures; SF-36, Short-Form 36 Health Survey; STAI, State-Trait Anxiety Inventory.

^a^ Five of the 32 inpatient recovery PROMs were unique (ie, these PROMs were not included in any outpatient studies [Table 1]); no PROMs assessed surgical complications or scar and wound healing domains as part of included inpatient recovery studies.

### Descriptive Data

**Figure 2.**
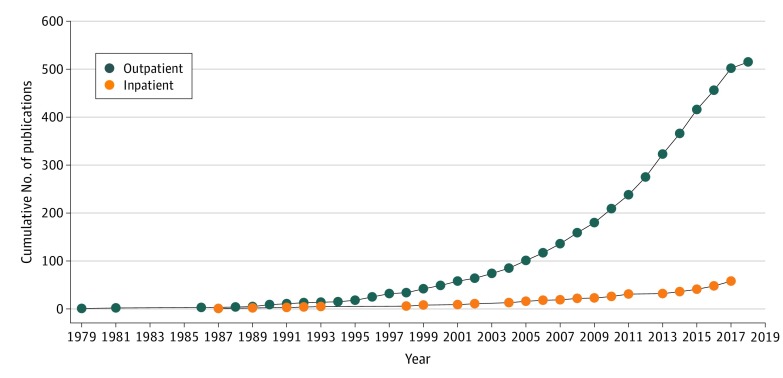
Cumulative Number of Publications Over Time Using Patient-Reported Outcome Measures to Assess Outpatient and Inpatient Recovery After Childbirth

Most outpatient and inpatient recovery studies were undertaken in the United States and published in psychiatry and obstetric and gynecology journals (Table 3). More than 80% of the outpatient studies were published within the past 13 years and more than 80% of the inpatient studies were published within the last 10 years (Figure 2).

**Table 3.** Summary of Included Studies

Characteristic	Value
**Outpatient**	
No. of articles	515
No. of PROMs	201
Journal specialty, No. of articles	
Obstetrics and gynecology	124
Psychiatry	126
Nursing	47
Medicine	47
Pediatrics	14
Anesthesia	11
Midwifery	24
Urogynecology surgery	43
Women’s health and sex medicine	32
Other	47
Country of publication, No. of articles	
United States	108
Australia	44
United Kingdom	38
Canada	26
Norway	23
Sweden	22
Japan	20
Italy	18
Iran	17
Taiwan	16
China	15
France	13
Brazil	10
Spain	12
Turkey	12
Other	121
**Inpatient**	
No. of articles	58
No. of PROMs	73 (32 unique to inpatient studies)
Journal specialty, No. of articles	
Obstetrics and gynecology	10
Psychiatry	14
Nursing	10
Medicine	7
Pediatrics	4
Anesthesia	4
Midwifery	3
Urogynecology surgery	0
Women’s health and sex medicine	1
Other	5
Country of publication	
United States	9
Italy	5
United Kingdom	4
Turkey	4
Taiwan	4
Poland	4
Sweden	3
Japan	3
France	3
Israel	2
Germany	2
Brazil	2
Other	11
Not stated	2

Abbreviation: PROMs, patient-reported outcome measures.

### Global Recovery PROMs

A total of 24 global recovery PROMs were identified among all included studies. Seventeen PROMs assessing global recovery were identified among the outpatient studies. The Short-Form 36 Health Questionnaire was the most frequently used global recovery PROM among these studies. Seven of the 17 global recovery PROMs were specifically designed for use in the postpartum population. One of these PROMs was a patient-generated item list (Mother-Generated Index)^22^ and the remaining 6 were standardized PROMs: Inventory of Functional Status After Childbirth,^23^ Barkin Index of Maternal Functioning,^24^ Maternal Concerns Questionnaire,^25^ Maternal Postpartum Quality of Life Questionnaire,^26^ Rural Postpartum Quality of Life,^27^ and Postpartum Symptom Checklist.^28^

Thirteen PROMs assessing global recovery were identified among the inpatient studies. The Short-Form 36 Health Questionnaire was the most frequently used global recovery PROM among inpatient studies. Seven of these 13 PROMs were specifically designed for use in the postpartum population. Of these 7 PROMs, 3 were also reported in outpatient studies (Inventory of Functional Status After Childbirth,^23^ Barkin Index of Maternal Functioning,^24^ and Postpartum Symptom Checklist^28^) and 4 PROMs were unique to the inpatient studies: Obstetric Quality of Recovery Score–11,^29^ Parents’ Postnatal Sense of Security Swedish instrument,^30^ Postpartum Comfort Questionnaire,^31^ and Recovery From Cesarean Section Scale.^32^

A total of 11 obstetric-specific PROMs were used to evaluate global recovery in the included 573 studies. The median number of domains evaluated by these PROMs was 5 (range, 4-10; eTable 2 and eTable 3 in the Supplement). Among the inpatient and outpatient global recovery PROMs included in this review, the Maternal Concerns Questionnaire^25^ evaluated the greatest number of recovery domains (10 of 12 domains). This is a 50-item questionnaire with scoring on a Likert scale from 1 to 4, with an option of writing additional concerns not listed in the questionnaire. This PROM was developed by a panel of 14 mothers within 1 year of delivery and 3 nurses and subsequently tested among 30 women at 3 days postpartum and 7 days after hospital discharge.^33^ This PROM has been used in 1 further published study^34^ included in the literature search.

## Discussion

The main finding from this scoping review is that there is heterogeneous use of PROMs to assess postpartum recovery. Most of the included studies that used PROMs assessed psychosocial distress. Similar PROMs were broadly used to assess recovery domains in both the inpatient and outpatient settings, with the exception of the maternal-neonatal bonding, fatigue, and cognition domains, which were featured more within inpatient studies. Measures of global recovery also differed among the outpatient and inpatient studies (such as the ObsQoR-11 used to assess inpatient recovery^29,35^). No PROMs were used to assess outpatient cognition or inpatient surgical complications and scar or wound healing issues.

Defining postpartum outpatient recovery through identification of specific domains and PROMs used is an important first step toward phenotyping postpartum recovery. Having identified recovery PROMs, further work is now needed to determine which of these instruments can best measure individual recovery domains as well as global recovery. This assessment and recommendation can be made through a series of systematic reviews of each recovery domain and psychometric evaluation of existing PROMs, using COSMIN methods.^14^ Studies are required to determine whether existing PROMs can be used effectively to measure recovery after different delivery modes, at different postpartum time points, and within high-resource and low-resource settings. If measures are not available to assess specific recovery domains, or if they perform poorly in measures of validity, reliability, and responsiveness to change, it may justify the development and validation of new PROMs specific to that domain of postpartum recovery. Methods described by the Patient-Reported Outcome Measurement Information System (PROMIS) group can be used to develop and validate a new PROM.^36^

The exponential increase in numbers of studies over time using PROMs to evaluate domains of outpatient recovery suggests that this aspect of obstetric care is clinically important yet still incompletely defined or understood. The interest in postpartum recovery across a breadth of countries also suggests that research in this area is relevant to women residing in multiple cultures and continents globally. The large number of PROMs available to measure psychological, psychiatric, and psychosocial factors after childbirth is reassuring because psychiatric disease has consistently been reported as a major factor associated with maternal mortality during the past 20 years. The UK Confidential Enquiries into Maternal Deaths report found that psychiatric disorders, and suicide in particular, were the leading cause of maternal deaths.^37,38^ In the most recent MBRACE-UK (Mothers and Babies: Reducing Risk through Audits and Confidential Enquiries Across the UK) report, maternal suicide was reported as the fifth most common cause of women’s deaths during pregnancy and was reported as the leading cause of death during the first year after pregnancy.^39^

The large number of PROMs available to assess symptoms of incontinence and prolapse may reflect the potential association with quality of life, the benefits of early diagnosis, and options for patient referral to physiotherapy and surgical subspecialties such as urogynecology, which may have resulted in increased popularity of these PROMs among postpartum recovery studies. As previously demonstrated by the paucity of recovery PROMs validated for use after cesarean delivery,^12^ evaluation of available recovery outcome measures through this review has helped to identify deficient areas. Although all domains are important, the low number of PROMs within some domains may be associated with the limited treatment options. Problems with breastfeeding, for example, may improve after input from a lactation consultant and pain may respond to pharmacotherapy. However, many of the other domains with smaller numbers of PROMs, such as sleep and fatigue, may have fewer therapeutic options available in the primary health care setting. The domains with fewer PROMs may be considered to be aspects of postpartum recovery that are currently underexplored, which may benefit from research to develop novel and effective interventions.

There is growing interest in applying PROMs to evaluate the performance of individual health care professionals, as a tool to benchmark hospital performance and determine value-based reimbursement for care delivered to patients.^11^ The lack of use of PROMs to guide obstetric care–related value-based reimbursement may partly be owing to the absence of a robustly validated and reliable PROM for use in this setting. Given the vast global expenditure on peripartum services and use of inpatient resources by parturients, this population seems an obvious choice for implementing payment according to quality of care delivered. Future work should focus on evaluating existing PROMs and developing new measures to assess global outpatient quality of recovery as a marker of care delivered.

### Limitations

This scoping review has several limitations. First, we screened only titles and abstracts of fully published articles for validated PROMs, which may have resulted in the exclusion of several validated PROMs. However, we think that this review provides a representative sample of PROMs used to assess recovery after childbirth, as the numbers of included articles and PROMs were substantial. Although the large number of included studies justifies our decision to adopt this approach, we were restricted in terms of granularity of data extracted, as not all full articles were retrieved to determine study and PROM quality. We acknowledge that recovery domain classification of PROMs is subjective. Rather than formulating a separate classification system for inpatient-specific domains, we used the outpatient domains of recovery to facilitate comparison of outpatient and inpatient distribution of PROMs among recovery domains. This approach also allowed us to determine whether an inpatient global recovery PROM could potentially be suitable for use in the outpatient setting. Although author discussion yielded 12 recovery domains, it is possible that different domains would emerge from concept elicitation interviews with key stakeholders such as patients, obstetricians, and nurses. We appreciate that objective measures of recovery (determined by a physician or health care professional) may also be effective at assessing recovery domains. For example, only 2 PROMs assessing breastfeeding were reported in this scoping review, as we excluded objective measures of breastfeeding success such as the LATCH score.^17^ To our knowledge, no studies have compared PROMs with objective measures of different recovery domains.

## Conclusions

Most PROMs identified in this review evaluated a single domain of recovery. This finding emphasizes the need to develop a measure that comprehensively assesses the multiple domains of postpartum recovery. Future research should focus on obtaining clinician and patient input on the symptoms and concerns viewed as most important to assess during recovery. The results of this review can be used to develop a conceptual framework to guide the development of a comprehensive measure of recovery that includes the most important domains. Further research is also needed to evaluate the quality of available PROMs and determine the best tool to measure each domain and global postpartum recovery.

## Notes

Supplement. **eMethods 1.** Literature Search Strategy **eMethods 2.** Development of Outpatient Recovery Domains (Adapted From Functional Recovery Domains Previously Described by Sharawi et al.^12^) **eResults.** References for the 515 Studies That Utilized Patient-Reported Outcome Measures (PROMs) to Evaluate Outpatient Recovery **eTable 1.** Most Frequently Utilized Patient-Reported Outcome Measures (PROMs) Among All Included (Outpatient and Inpatient) Studies **eTable 2.** Classification of 201 Patient-Reported Outcome Measures (PROMs) Used to Evaluate Outpatient Recovery Following Childbirth According to Domains **eTable 3.** Summary of 73 Patient-Reported Outome Measures (PROMs) Used to Evaluate Inpatient Recovery Following Childbirth
