# Supplementary material for: Evaluation of Domains of Patient-Reported Outcome Measures for Recovery After Childbirth: A Scoping and Systematic Review
Source: JAMA Netw Open. 2020 May 22;3(5):e205540. doi: 10.1001/jamanetworkopen.2020.5540 (PMC7244991; doi:10.1001/jamanetworkopen.2020.5540)
Supplement: Supplement. — eMethods 1. Literature Search Strategy eMethods 2. Development of Outpatient Recovery Domains (Adapted From Functional Recovery Domains Previously Described by Sharawi et al.12) eResults. References for the 515 Studies That Utilized Patient-Reported Outcome Measures (PROMs) to Evaluate Outpatient Recovery eTable 1. Most Frequently Utilized Patient-Reported Outcome Measures (PROMs) Among All Included (Outpatient and Inpatient) Studies eTable 2. Classification of 201 Patient-Reported Outcome Measures (PROMs) Used to Evaluate Outpatient Recovery Following Childbirth According to Domains eTable 3. Summary of 73 Patient-Reported Outome Measures (PROMs) Used to Evaluate Inpatient Recovery Following Childbirth [file jamanetwopen-3-e205540-s001.pdf]

Supplementary Online Content

Sultan P, Sadana N, Sharawi N, et al. Evaluation of domains of patient-reported outcome measures for recovery after childbirth: a scoping and systematic review. *JAMA Netw Open.* 2020;3(5):e205540. doi:10.1001/jamanetworkopen.2020.5540

eMethods 1. Literature Search Strategy
eMethods 2. Development of Outpatient Recovery Domains (Adapted From Functional Recovery Domains Previously Described by Sharawi et al.[12])
eResults. References for the 515 Studies That Utilized Patient-Reported Outcome Measures (PROMs) to Evaluate Outpatient Recovery
eTable 1. Most Frequently Utilized Patient-Reported Outcome Measures (PROMs) Among All Included (Outpatient and Inpatient) Studies
eTable 2. Classification of 201 Patient-Reported Outcome Measures (PROMs) Used to Evaluate Outpatient Recovery Following Childbirth According to Domains
eTable 3. Summary of 73 Patient-Reported Outcome Measures (PROMs) Used to Evaluate Inpatient Recovery Following Childbirth

This supplementary material has been provided by the authors to give readers additional information about their work.

© 2020 Sultan P et al. *JAMA Network Open.*

eMethods 1. Literature Search Strategy

PubMed – July 1, 2019 – 6,082 results
("Delivery, Obstetric"[Mesh] OR "Labor, Induced"[Mesh] OR "Extraction, Obstetrical"[Mesh] OR “instrumental delivery” OR “vaginal delivery” OR “vaginal birth” OR “vacuum delivery” OR “vacuum assisted delivery” OR “forceps assisted delivery” OR childbirth) AND ("Postpartum Period"[Mesh] OR "Postnatal Care"[Mesh] OR intrapartum OR postpartum OR postnatal OR "Postoperative Complications"[Mesh] OR "Postoperative Care"[Mesh] OR "Postoperative Period"[Mesh] OR “postoperative” OR “post-operative” or “postsurgery” OR “post surgical” OR “postop” OR recovery OR "Recovery Room"[Mesh] OR "recovery room" OR “post-anesthesia” OR “post-anaesthesia” OR “pacu” OR ((post-anesthesia OR post-anaesthesia) AND acute care unit)) NOT ("Prenatal Care"[Mesh] OR "Prenatal Diagnosis"[Mesh] OR "Prenatal Education"[Mesh] OR prenatal OR antenatal)

AND (("Recovery of Function"[Mesh] OR "Activities of Daily Living"[Mesh] OR "Delayed Emergence from Anesthesia"[Mesh] OR "Fatigue"[Mesh] OR"Anesthesia Recovery Period"[Mesh] OR "Emergence Delirium"[Mesh] OR "Acclimatization"[Mesh] OR "Adaptation, Physiological"[Mesh] OR "Maternal Health"[Mesh] OR “maternal health” OR caretaker OR adaptation OR “fatigue” OR “eat” OR “eating” OR “drink” OR “drinking” OR "Postoperative Nausea and Vomiting"[Mesh] OR "Lethargy"[Mesh] OR nausea OR vomiting))
OR ("Female Urogenital Diseases and Pregnancy Complications"[Mesh] OR "Urinary Incontinence"[Mesh] OR "Uterine Prolapse"[Mesh] OR "Dysmenorrhea"[Mesh] OR "Fecal Incontinence"[Mesh] OR "Gastrointestinal Tract"[Mesh] OR “colorectal” OR “colon” OR rectum OR anus OR urogenital OR urological OR gynecological OR “urinary incontinence” OR “dysmenorrhea” OR “uterine prolapse” OR “fecal incontinence”)
OR ("Pain Management"[Mesh] OR "Pain, Postoperative"[Mesh] OR “pain” OR "Myalgia"[Mesh] OR myalgia OR "Pelvic Pain"[Mesh] OR "Pain"[Mesh] OR “pelvic pain” OR "Headache"[Mesh] OR headache OR "Back Pain"[Mesh] OR “back pain”)
OR (("Mental Health Recovery"[Mesh] OR "Anxiety"[Mesh] OR "Stress Disorders, Post-Traumatic"[Mesh] OR "Depression, Postpartum"[Mesh] OR "Psychotic Disorders"[Mesh] OR "Mood Disorders"[Mesh] OR "Suicidal Ideation"[Mesh] OR "Self-Injurious Behavior"[Mesh] OR "Mental Fatigue"[Mesh] OR "Emotional Adjustment"[Mesh] OR "Pleasure"[Mesh] OR "Happiness"[Mesh] OR "Motivation"[Mesh] OR "Guilt"[Mesh] OR "Social Desirability"[Mesh] OR "Hope"[Mesh] OR "Stress, Psychological"[Mesh] OR "Psychomotor Performance"[Mesh] OR "Appetite"[Mesh] OR "Personal Satisfaction"[Mesh] OR "Adaptation, Psychological"[Mesh] OR "Perception"[Mesh] OR "Bipolar Disorder"[Mesh] OR "Phobic Disorders"[Mesh] OR "Adjustment Disorders"[Mesh] OR "Dissociative Disorders"[Mesh] OR "Dissociative Identity Disorder"[Mesh] OR "Delirium"[Mesh] OR "Neurocognitive Disorders"[Mesh] OR "Cognitive Dysfunction"[Mesh] OR "Behavior"[Mesh] OR "Conduct Disorder"[Mesh] OR "Disruptive, Impulse Control, and Conduct Disorders"[Mesh] OR "Restless Legs Syndrome"[Mesh] OR "Substance-Related Disorders"[Mesh] OR "Personality"[Mesh] OR "Amnesia"[Mesh] OR "Depersonalization"[Mesh] OR "Factitious Disorders"[Mesh] OR "Somatoform Disorders"[Mesh] OR "Conversion Disorder"[Mesh] OR "psychological recovery" OR "mental health recovery" OR

© 2020 Sultan P et al. *JAMA Network Open.*

“mental fatigue” OR "mental health" OR “depression” OR “depressing” OR “depressed” OR anxiety OR anxious OR perpetual psychosis OR “baby blues” OR “mood disorder” OR “self-harm” OR “suicidal ideation” OR “post-traumatic stress disorder” OR “ptsd” OR psychosis OR fatigue OR “emotional” OR (emotion AND control) OR “enjoy” OR enjoyable OR enjoyment OR motivation OR guilt OR wellbeing OR “worth” OR worthless OR worthlessness OR worthy OR “hope” OR hopeful OR hopeless OR hopelessness OR psychomotor OR “self-control” OR appetite OR coping OR perception OR “mania” OR “phobia” OR adjustment OR “reactive” OR dissociative OR “illness anxiety” OR conversion OR factitious OR depersonalization OR amnesia OR somatic OR substance abuse OR “restless leg” OR neurocognitive OR delirium))
OR ("Social Support"[Mesh] OR "Psychosocial Support Systems"[Mesh] OR “social support” OR "Social Adjustment"[Mesh] OR "Social Isolation"[Mesh] OR "Interpersonal Relations"[Mesh] OR “social isolation” OR "Social Adjustment"[Mesh] OR "Social Participation"[Mesh] OR "Social Behavior"[Mesh])
OR ("Sleep"[Mesh] OR “sleep” OR “sleeping” OR “awake” OR insomnia OR narcolepsy OR "Narcolepsy"[Mesh] OR "Sleep Initiation and Maintenance Disorders"[Mesh] OR "Sleep Latency"[Mesh] OR “sleep latency” OR "Sleep Wake Disorders"[Mesh])
OR ("Arousal"[Mesh] OR "Coitus"[Mesh] OR "Sexual Health"[Mesh] OR "Reproductive Health"[Mesh] OR Coitus OR intercourse OR “sexual health” OR "Sexual Dysfunctions, Psychological"[Mesh] OR "Orgasm"[Mesh] OR orgasm OR "Dyspareunia"[Mesh] OR dyspareunia)
OR ("Breast Feeding"[Mesh] OR "Object Attachment"[Mesh] OR "Infant Care"[Mesh] OR “breast feeding” OR bonding OR “infant care”)
AND ((instrumentation[sh] OR methods[sh] OR "Validation Studies"[pt] OR "Comparative Study"[pt] OR "psychometrics"[MeSH] OR psychometr*[tiab] OR clinimetr*[tw] OR clinometr*[tw] OR "outcome assessment (health care)"[MeSH] OR "outcome assessment"[tiab] OR "outcome measure*"[tw] OR "observer variation"[MeSH] OR "observer variation"[tiab] OR "Health Status Indicators"[Mesh] OR "reproducibility of results"[MeSH] OR reproducib*[tiab] OR "discriminant analysis"[MeSH] OR reliab*[tiab] OR unreliab*[tiab] OR valid*[tiab] OR "coefficient of variation"[tiab] OR coefficient[tiab] OR homogeneity[tiab] OR homogeneous[tiab] OR "internal consistency"[tiab] OR (cronbach*[tiab] AND (alpha[tiab] OR alphas[tiab])) OR (item[tiab] AND (correlation*[tiab] OR selection*[tiab] OR reduction*[tiab])) OR agreement[tw] OR precision[tw] OR imprecision[tw] OR "precise values"[tw] OR test-retest[tiab] OR (test[tiab] AND retest[tiab]) OR (reliab*[tiab] AND (test[tiab] OR retest[tiab])) OR stability[tiab] OR interrater[tiab] OR inter-rater[tiab] OR intrarater[tiab] OR intra-rater[tiab] OR intertester[tiab] OR inter-tester[tiab] OR intratester[tiab] OR intra-tester[tiab] OR interobserver[tiab] OR inter-observer[tiab] OR intraobserver[tiab] OR intra-observer[tiab] OR intertechnician[tiab] OR inter-technician[tiab] OR intratechnician[tiab] OR intra-technician[tiab] OR interexaminer[tiab] OR inter-examiner[tiab] OR intraexaminer[tiab] OR intra-examiner[tiab] OR interassay[tiab] OR inter-assay[tiab] OR intraassay[tiab] OR intra-assay[tiab] OR interindividual[tiab] OR inter-individual[tiab] OR intraindividual[tiab] OR intra-individual[tiab] OR interparticipant[tiab] OR inter-participant[tiab] OR intraparticipant[tiab] OR intra-participant[tiab] OR kappa[tiab] OR kappa's[tiab] OR kappas[tiab] OR repeatab*[tw] OR ((replicab*[tw] OR repeated[tw]) AND (measure[tw] OR measures[tw] OR findings[tw] OR result[tw] OR results[tw] OR test[tw] OR tests[tw])) OR

© 2020 Sultan P et al. *JAMA Network Open.*

generaliza*[tiab] OR generalisa*[tiab] OR concordance[tiab] OR (intraclass[tiab] AND correlation*[tiab]) OR discriminative[tiab] OR "known group"[tiab] OR "factor analysis"[tiab] OR "factor analyses"[tiab] OR "factor structure"[tiab] OR "factor structures"[tiab] OR dimension*[tiab] OR subscale*[tiab] OR (multitrait[tiab] AND scaling[tiab] AND (analysis[tiab] OR analyses[tiab])) OR "item discriminant"[tiab] OR "interscale correlation*"[tiab] OR error[tiab] OR errors[tiab] OR "individual variability"[tiab])OR "interval variability"[tiab] OR "rate variability"[tiab] OR (variability[tiab] AND (analysis[tiab] OR values[tiab])) OR (uncertainty[tiab] AND (measurement[tiab] OR measuring[tiab])) OR "standard error of measurement"[tiab] OR sensitiv*[tiab] OR responsive*[tiab] OR (limit[tiab] AND detection[tiab]) OR "minimal detectable concentration"[tiab] OR interpretab*[tiab] OR ((minimal[tiab] OR minimally[tiab] OR clinical[tiab] OR clinically[tiab]) AND (important[tiab] OR significant[tiab] OR detectable[tiab]) AND (change[tiab] OR difference[tiab])) OR (small*[tiab] AND (real[tiab] OR detectable[tiab]) AND (change[tiab] OR difference[tiab])) OR "meaningful change"[tiab] OR "ceiling effect"[tiab] OR "floor effect"[tiab] OR "Item response model"[tiab] OR IRT[tiab] OR Rasch[tiab] OR "Differential item functioning"[tiab] OR DIF[tiab] OR "computer adaptive testing"[tiab] OR "item bank"[tiab] OR "cross-cultural equivalence"[tiab])
NOT (("Delphi Technique"[Mesh] OR "Cross-Sectional Studies"[Mesh] OR "addresses"[Publication Type] OR "biography"[Publication Type] OR "case reports"[Publication Type] OR "comment"[Publication Type] OR "directory"[Publication Type] OR "editorial"[Publication Type] OR "festschrift"[Publication Type] OR "interview"[Publication Type] OR "lectures"[Publication Type] OR "legal cases"[Publication Type] OR "legislation"[Publication Type] OR "letter"[Publication Type] OR "news"[Publication Type] OR "newspaper article"[Publication Type] OR "patient education handout"[Publication Type] OR "popular works"[Publication Type] OR "congresses"[Publication Type] OR "consensus development conference"[Publication Type] OR "consensus development conference, nih"[Publication Type] OR "practice guideline"[Publication Type]) NOT ("animals"[MeSH Terms] NOT "humans"[MeSH Terms]))
Filters: English

Web of Science – July 1, 2019 – 2,114 results
("Obstetric Delivery" OR "Induced Labor" OR "Obstetrical Extraction " OR “instrumental delivery” OR “vaginal delivery” OR “vaginal birth” OR “vacuum delivery” OR “vacuum assisted delivery” OR “forceps assisted delivery” OR childbirth) AND ("Postpartum Period" OR "Postnatal Care" OR intrapartum OR postpartum OR postnatal OR "Postoperative Complications" OR "Postoperative Care" OR "Postoperative Period" OR “postoperative” OR “post-operative” or “postsurgery” OR “post surgical” OR “postop” OR recovery OR "recovery room" OR “post-anesthesia” OR “post-anaesthesia” OR “pacu” OR ((post-anesthesia OR post-anaesthesia) AND acute care unit)) NOT ("Prenatal Care" OR "Prenatal Diagnosis" OR "Prenatal Education" OR prenatal OR antenatal)

AND ("Recovery of Function" OR "Activities of Daily Living" OR "Delayed Emergence from Anesthesia" OR "Fatigue" OR "Anesthesia Recovery Period" OR "Emergence Delirium" OR "Acclimatization" OR "Adaptation, Physiological" OR "Maternal Health" OR caretaker OR adaptation OR “fatigue” OR “eat” OR “eating” OR “drink” OR “drinking” OR "Postoperative Nausea and Vomiting" OR "Lethargy"

© 2020 Sultan P et al. *JAMA Network Open.*

OR nausea OR vomiting) OR ("Female Urogenital Diseases and Pregnancy Complications" OR "Urinary Incontinence" OR "Uterine Prolapse" OR "Dysmenorrhea" OR "Fecal Incontinence" OR "Gastrointestinal Tract" OR “colorectal” OR “colon” OR rectum OR anus OR urogenital OR urological OR gynecological OR “urinary incontinence” OR “dysmenorrhea” OR “uterine prolapse” OR “fecal incontinence”) OR ("Pain Management" OR "Pain, Postoperative" OR “pain” OR "Myalgia" OR “pelvic pain” OR "Headache" OR “back pain”) OR ("Mental Health Recovery" OR "Anxiety" OR " Post-Traumatic Stress Disorders" OR "Postpartum Depression" OR "Psychotic Disorders" OR "Mood Disorders" OR "Suicidal Ideation" OR "Self-Injurious Behavior" OR "Mental Fatigue" OR "Emotional Adjustment" OR "Pleasure" OR "Happiness" OR "Motivation" OR "Guilt" OR "Social Desirability" OR "Stress" OR "Psychomotor Performance" OR "Appetite" OR "Personal Satisfaction" OR " Psychological Adaptation" OR "Perception" OR "Bipolar Disorder" OR "Phobic Disorders" OR "Adjustment Disorders" OR "Dissociative Disorders" OR "Dissociative Identity Disorder" OR "Delirium" OR "Neurocognitive Disorders" OR "Cognitive Dysfunction" OR "Behavior" OR "Conduct Disorder" OR "Disruptive, Impulse Control, and Conduct Disorders" OR "Restless Legs Syndrome" OR "Substance-Related Disorders" OR "Personality" OR "Amnesia" OR "Depersonalization" OR "Factitious Disorders" OR "Somatoform Disorders" OR "Conversion Disorder" OR "psychological recovery" OR "mental health" OR “depression” OR “depressing” OR “depressed” OR anxiety OR anxious OR perpetual psychosis OR “baby blues” OR “mood disorder” OR “self-harm” OR “ptsd” OR psychosis OR fatigue OR “emotional” OR (emotion AND control) OR “enjoy” OR enjoyable OR enjoyment OR wellbeing OR “worth” OR worthless OR worthlessness OR worthy OR “hope” OR hopeful OR hopeless OR hopelessness OR psychomotor OR “self-control” OR coping OR perception OR “mania” OR “phobia” OR adjustment OR “reactive” OR dissociative OR “illness anxiety” OR somatic OR neurocognitive OR delirium) OR ("Social Support" OR "Psychosocial Support Systems" OR "Social Adjustment" OR "Social Isolation" OR "Interpersonal Relations" OR “social isolation” OR "Social Adjustment" OR "Social Participation" OR "Social Behavior") OR ("Sleep" OR “sleeping” OR “awake” OR insomnia OR narcolepsy OR "Sleep Initiation and Maintenance Disorders" OR “sleep latency” OR "Sleep Wake Disorders") OR ("Arousal" OR "Coitus" OR "Sexual Health" OR "Reproductive Health" OR Coitus OR intercourse OR "Sexual Dysfunctions" OR orgasm OR dyspareunia) OR ("Breast Feeding" OR "Object Attachment" OR "Infant Care" OR bonding)
AND (((instrumentation OR methods OR "Validation Studies" OR "Comparative Study" OR "psychometrics" OR psychometr* OR clinimetr* OR clinometr* OR "health care outcome assessment" OR "outcome assessment" OR "outcome measure*" OR "observer variation" OR "observer variation" OR "Health Status Indicators" OR "reproducibility of results" OR reproducib* OR "discriminant analysis" OR reliab* OR unreliab* OR valid* OR "coefficient of variation" OR coefficient OR homogeneity OR homogeneous OR "internal consistency" OR (cronbach* AND (alpha OR alphas)) OR (item AND (correlation* OR selection* OR reduction*)) OR agreement OR precision OR imprecision OR "precise values" OR test-retest OR (test AND retest) OR (reliab* AND (test OR retest)) OR stability OR interrater OR inter-rater OR intrarater OR intra-rater OR intertester OR inter-tester OR intratester OR intra-tester OR interobserver OR inter-observer OR intraobserver OR intra-observer OR intertechnician OR inter-technician OR intratechnician OR intra-technician OR interexaminer OR inter-examiner OR intraexaminer OR intra-

© 2020 Sultan P et al. *JAMA Network Open.*

examiner OR interassay OR inter-assay OR intraassay OR intra-assay OR interindividual OR inter-individual OR intraindividual OR intra-individual OR interparticipant OR inter-participant OR intraparticipant OR intra-participant OR kappa OR kappa's OR kappas OR repeatab* OR ((replicab* OR repeated) AND (measure OR measures OR findings OR result OR results OR test OR tests)) OR generaliza* OR generalisa* OR concordance OR (intraclass AND correlation*) OR discriminative OR "known group" OR "factor analysis" OR "factor analyses" OR "factor structure" OR "factor structures" OR dimension* OR subscale* OR (multitrait AND scaling AND (analysis OR analyses)) OR "item discriminant" OR "interscale correlation*" OR error OR errors OR "individual variability") OR "interval variability" OR "rate variability" OR (variability AND (analysis OR values)) OR (uncertainty AND (measurement OR measuring)) OR "standard error of measurement" OR sensitiv* OR responsive* OR (limit AND detection) OR "minimal detectable concentration" OR interpretab* OR ((minimal OR minimally OR clinical OR clinically) AND (important OR significant OR detectable) AND (change OR difference)) OR (small* AND (real OR detectable) AND (change OR difference)) OR "meaningful change" OR "ceiling effect" OR "floor effect" OR "Item response model" OR IRT OR Rasch OR "Differential item functioning" OR DIF OR "computer adaptive testing" OR "item bank" OR "cross-cultural equivalence"))
NOT ((("Delphi Technique" OR "Cross-Sectional Studies" OR "addresses" OR "biography" OR "case reports" OR "comment" OR "directory" OR "editorial" OR "festschrift" OR "interview" OR "lectures" OR "legal cases" OR "legislation" OR "letter" OR "news" OR "newspaper article" OR "patient education handout" OR "popular works" OR "congresses" OR "consensus development conference" OR "nih consensus development conference" OR "practice guideline") NOT ("animals" NOT "humans")))
Filters: English

CINAHL – July 1, 2019 – 1,035 results
(MH "Labor, Induced" OR MH "Vacuum Extraction, Obstetrical" OR MH "Delivery, Obstetric" OR MH "Vaginal Birth" OR "Delivery, Obstetric" OR "Labor, Induced" OR "Extraction, Obstetrical" OR “instrumental delivery” OR “vaginal delivery” OR “vaginal birth” OR “vacuum delivery” OR “vacuum assisted delivery” OR “forceps assisted delivery” OR childbirth) AND (MH "Postnatal Care" OR MH "Postnatal Period" OR MH "Postoperative Period" OR MH "Postoperative Care" OR MH "Post Anesthesia Care" OR "Postpartum Period" OR "Postnatal Care" OR intrapartum OR postpartum OR postnatal OR "Postoperative Complications" OR "Postoperative Care" OR "Postoperative Period" OR “postoperative” OR “post-operative” or “postsurgery” OR “post surgical” OR “postop” OR recovery OR "recovery room" OR “post-anesthesia” OR “post-anaesthesia” OR “pacu” OR ((post-anesthesia OR post-anaesthesia) AND acute care unit)) NOT ("Prenatal Care" OR "Prenatal Diagnosis" OR "Prenatal Education" OR prenatal OR antenatal)

AND ((MH "Recovery") OR (MH "Anesthesia Recovery") OR (MH "Activities of Daily Living") OR (MH "Physical Activity") OR (MH "Delirium") OR (MH "Adaptation, Physiological") OR (MH "Adaptation, Psychological") OR (MH "Acclimatization") OR (MH "Nausea") OR (MH "Nausea and Vomiting") OR (MH "Vomiting") OR (MH "Appetite") OR (MH "Fatigue") OR (MH "Mental Fatigue") OR (MH "Muscle Fatigue") OR (MH "Incontinence") OR (MH "Uterine Prolapse") OR (MH "Uterine Hemorrhage") OR (MH "Rectal Prolapse") OR (MH "Uterine

© 2020 Sultan P et al. *JAMA Network Open.*

Inversion") OR (MH "Metrorrhagia") OR (MH "Pelvic Organ Prolapse") OR (MH "Uterine Rupture") OR (MH "Pelvic Pain") OR (MH "Abdominal Pain") OR (MH "Postoperative Pain") OR (MH "Back Pain") OR (MH "Pain") OR (MH "Depression") OR (MH "Depression, Postpartum") OR (MH "Bipolar Disorder") OR (MH "Stress") OR (MH "Stress Disorders, Post-Traumatic") OR (MH "Pleasure") OR (MH "Hope") OR (MH "Optimism") OR (MH "Hopelessness") OR (MH "Self Concept") OR (MH "Personality") OR (MH "Amnesia") OR (MH "Breast Feeding") OR (MH "Mental Health") OR (MH "Sexual Health") OR (MH "Sexual Dysfunction, Female") OR (MH "Coitus") OR (MH "Self-Injurious Behavior") OR (MH "Personal Satisfaction") OR (MH "Sexual Satisfaction") OR (MH "Headache") OR (MH "Muscle Pain") OR (MH "Anxiety") OR (MH "Adjustment Disorders") OR (MH "Dyssomnias") OR (MH "Sleep") OR (MH "Sleep-Wake Transition Disorders") OR (MH "Parasomnias") OR (MH "Sleep Arousal Disorders") OR (MH "Sleep Disorders") OR (MH "Social Behavior Disorders") OR (MH "Social Anxiety Disorders") OR (MH "Social Adjustment") OR (MH "Social Isolation") OR (MH "Parent-Infant Bonding") OR (MH "Infant Care") OR (MH "Perception") OR (MH "Dissociative Disorders") OR (MH "Suicidal Ideation") OR (MH "Happiness") OR (MH "Motivation") OR (MH "Phobic Disorders") OR (MH "Cognition Disorders") OR (MH "Behavior") OR (MH "Parental Behavior") OR (MH "Emotions") OR "Recovery of Function" OR "Activities of Daily Living" OR "Delayed Emergence from Anesthesia" OR "Fatigue" OR "Anesthesia Recovery Period" OR "Emergence Delirium" OR "Acclimatization" OR "Adaptation, Physiological" OR "Maternal Health" OR caretaker OR adaptation OR “fatigue” OR “eat” OR “eating” OR “drink” OR “drinking” OR "Postoperative Nausea and Vomiting" OR "Lethargy" OR nausea OR vomiting) OR ("Female Urogenital Diseases and Pregnancy Complications" OR "Urinary Incontinence" OR "Uterine Prolapse" OR "Dysmenorrhea" OR "Fecal Incontinence" OR "Gastrointestinal Tract" OR “colorectal” OR “colon” OR rectum OR anus OR urogenital OR urological OR gynecological OR “urinary incontinence” OR “dysmenorrhea” OR “uterine prolapse” OR “fecal incontinence”) OR ("Pain Management" OR "Pain, Postoperative" OR “pain” OR "Myalgia" OR “pelvic pain” OR "Headache" OR “back pain”) OR ("Mental Health Recovery" OR "Anxiety" OR " Post-Traumatic Stress Disorders" OR "Postpartum Depression" OR "Psychotic Disorders" OR "Mood Disorders" OR "Suicidal Ideation" OR "Self-Injurious Behavior" OR "Mental Fatigue" OR "Emotional Adjustment" OR "Pleasure" OR "Happiness" OR "Motivation" OR "Guilt" OR "Social Desirability" OR "Stress" OR "Psychomotor Performance" OR "Appetite" OR "Personal Satisfaction" OR " Psychological Adaptation" OR "Perception" OR "Bipolar Disorder" OR "Phobic Disorders" OR "Adjustment Disorders" OR "Dissociative Disorders" OR "Dissociative Identity Disorder" OR "Delirium" OR "Neurocognitive Disorders" OR "Cognitive Dysfunction" OR "Behavior" OR "Conduct Disorder" OR "Disruptive, Impulse Control, and Conduct Disorders" OR "Restless Legs Syndrome" OR "Substance-Related Disorders" OR "Personality" OR "Amnesia" OR "Depersonalization" OR "Factitious Disorders" OR "Somatoform Disorders" OR "Conversion Disorder" OR "psychological recovery" OR "mental health" OR “depression” OR “depressing” OR “depressed” OR anxiety OR anxious OR perpetual psychosis OR “baby blues” OR “mood disorder” OR “self-harm” OR “ptsd” OR psychosis OR fatigue OR “emotional” OR (emotion AND control) OR “enjoy” OR enjoyable OR enjoyment OR wellbeing OR “worth” OR worthless OR worthlessness OR worthy OR “hope” OR hopeful OR hopeless OR hopelessness OR psychomotor OR “self-control” OR coping OR perception OR “mania” OR “phobia” OR

adjustment OR “reactive” OR dissociative OR “illness anxiety” OR somatic OR neurocognitive OR delirium) OR ("Social Support" OR "Psychosocial Support Systems" OR "Social Adjustment" OR "Social Isolation" OR "Interpersonal Relations" OR “social isolation” OR "Social Adjustment" OR "Social Participation" OR "Social Behavior") OR ("Sleep" OR “sleeping” OR “awake” OR insomnia OR narcolepsy OR "Sleep Initiation and Maintenance Disorders" OR “sleep latency” OR "Sleep Wake Disorders") OR ("Arousal" OR "Coitus" OR "Sexual Health" OR "Reproductive Health" OR Coitus OR intercourse OR "Sexual Dysfunctions" OR orgasm OR dyspareunia) OR ("Breast Feeding" OR "Object Attachment" OR "Infant Care" OR bonding)
AND (((instrumentation OR methods OR "Validation Studies" OR "Comparative Study" OR "psychometrics" OR psychometr* OR clinimetr* OR clinometr* OR "health care outcome assessment" OR "outcome assessment" OR "outcome measure*" OR "observer variation" OR "observer variation" OR "Health Status Indicators" OR "reproducibility of results" OR reproducib* OR "discriminant analysis" OR reliab* OR unreliab* OR valid* OR "coefficient of variation" OR coefficient OR homogeneity OR homogeneous OR "internal consistency" OR (cronbach* AND (alpha OR alphas)) OR (item AND (correlation* OR selection* OR reduction*)) OR agreement OR precision OR imprecision OR "precise values" OR test-retest OR (test AND retest) OR (reliab* AND (test OR retest)) OR stability OR interrater OR inter-rater OR intrarater OR intra-rater OR intertester OR inter-tester OR intratester OR intra-tester OR interobserver OR inter-observer OR intraobserver OR intra-observer OR intertechnician OR inter-technician OR intratechnician OR intra-technician OR interexaminer OR inter-examiner OR intraexaminer OR intra-examiner OR interassay OR inter-assay OR intraassay OR intra-assay OR interindividual OR inter-individual OR intraindividual OR intra-individual OR interparticipant OR inter-participant OR intraparticipant OR intra-participant OR kappa OR kappa's OR kappas OR repeatab* OR ((replicab* OR repeated) AND (measure OR measures OR findings OR result OR results OR test OR tests)) OR generaliza* OR generalisa* OR concordance OR (intraclass AND correlation*) OR discriminative OR "known group" OR "factor analysis" OR "factor analyses" OR "factor structure" OR "factor structures" OR dimension* OR subscale* OR (multitrait AND scaling AND (analysis OR analyses)) OR "item discriminant" OR "interscale correlation*" OR error OR errors OR "individual variability") OR "interval variability" OR "rate variability" OR (variability AND (analysis OR values)) OR (uncertainty AND (measurement OR measuring)) OR "standard error of measurement" OR sensitiv* OR responsive* OR (limit AND detection) OR "minimal detectable concentration" OR interpretab* OR ((minimal OR minimally OR clinical OR clinically) AND (important OR significant OR detectable) AND (change OR difference)) OR (small* AND (real OR detectable) AND (change OR difference)) OR "meaningful change" OR "ceiling effect" OR "floor effect" OR "Item response model" OR IRT OR Rasch OR "Differential item functioning" OR DIF OR "computer adaptive testing" OR "item bank" OR "cross-cultural equivalence"))
NOT ((("Delphi Technique" OR "Cross-Sectional Studies" OR "addresses" OR "biography" OR "case reports" OR "comment" OR "directory" OR "editorial" OR "festschrift" OR "interview" OR "lectures" OR "legal cases" OR "legislation" OR "letter" OR "news" OR "newspaper article" OR "patient education handout" OR "popular works" OR "congresses" OR "consensus development conference" OR "nih consensus development conference" OR "practice guideline") NOT ("animals" NOT "humans")))

© 2020 Sultan P et al. *JAMA Network Open.*

Filters: English, Academic Journals and Dissertations

EMBASE – July 1, 2019 – 981 results
('delivery, obstetric'/de OR 'labor, induced'/de OR 'extraction, obstetrical'/de OR 'instrumental delivery'/de OR 'vaginal delivery'/de OR 'vaginal birth'/de OR 'vacuum delivery'/de OR 'vacuum assisted delivery'/de OR 'forceps assisted delivery' OR 'childbirth'/de) AND ('postpartum period'/de OR 'postnatal care'/de OR intrapartum OR 'postpartum'/de OR postnatal OR 'postoperative complications'/de OR 'postoperative care'/de OR 'postoperative period'/de OR 'postoperative' OR 'post-operative' OR 'postsurgery' OR 'post surgical' OR 'postop' OR 'recovery'/de OR 'recovery room'/de OR 'post-anesthesia' OR 'post-anaesthesia' OR 'pacu' OR (('post anesthesia' OR 'post anaesthesia') AND acute AND 'care'/de AND 'unit'/de))

AND 'recovery of function'/de OR 'activities of daily living'/de OR 'delayed emergence from anesthesia'/de OR 'anesthesia recovery period'/de OR 'emergence delirium'/de OR 'acclimatization'/de OR 'adaptation, physiological'/de OR 'maternal health'/de OR caretaker OR 'adaptation'/de OR 'eat' OR 'eating'/de OR 'drink' OR 'drinking'/de OR 'postoperative nausea and vomiting'/de OR 'lethargy'/de OR 'nausea'/de OR 'vomiting'/de OR 'female urogenital diseases and pregnancy complications'/de OR 'gastrointestinal tract'/de OR 'colorectal' OR 'colon'/de OR 'rectum'/de OR 'anus'/de OR urogenital OR urological OR gynecological OR 'urinary incontinence'/de OR 'dysmenorrhea'/de OR 'uterine prolapse'/de OR 'fecal incontinence'/de OR 'pain management'/de OR 'pain, postoperative'/de OR 'pain'/de OR 'myalgia'/de OR 'pelvic pain'/de OR 'headache'/de OR 'back pain'/de OR (('mental health recovery'/de OR 'post-traumatic stress disorders' OR 'postpartum depression'/de OR 'psychotic disorders'/de OR 'mood disorders'/de OR 'suicidal ideation'/de OR 'self-injurious behavior'/de OR 'mental fatigue'/de OR 'emotional adjustment'/de OR 'pleasure'/de OR 'happiness'/de OR 'motivation'/de OR 'guilt'/de OR 'social desirability'/de OR 'stress'/de OR 'psychomotor performance'/de OR 'appetite'/de OR 'personal satisfaction'/de OR 'psychological adaptation'/de OR 'perception'/de OR 'bipolar disorder'/de OR 'phobic disorders'/de OR 'adjustment disorders'/de OR 'dissociative disorders'/de OR 'dissociative identity disorder'/de OR 'delirium'/de OR 'neurocognitive disorders'/de OR 'cognitive dysfunction'/de OR 'behavior'/de OR 'conduct disorder'/de OR 'disruptive, impulse control, and conduct disorders'/de OR 'restless legs syndrome'/de OR 'substance-related disorders'/de OR 'personality'/de OR 'amnesia'/de OR 'depersonalization'/de OR 'factitious disorders'/de OR 'somatoform disorders'/de OR 'conversion disorder'/de OR 'psychological recovery' OR 'mental health'/de OR 'depression'/de OR 'depressing' OR 'depressed' OR 'anxiety'/de OR anxious OR perpetual) AND 'psychosis'/de) OR 'baby blues' OR 'mood disorder'/de OR 'self-harm'/de OR 'ptsd'/de OR 'psychosis'/de OR 'fatigue'/de OR 'emotional' OR ('emotion'/de AND 'control'/de) OR 'enjoy' OR enjoyable OR 'enjoyment'/de OR 'wellbeing'/de OR 'worth' OR worthless OR 'worthlessness'/de OR worthy OR 'hope'/de OR hopeful OR hopeless OR 'hopelessness'/de OR psychomotor OR 'self-control'/de OR 'coping'/de OR 'perception'/de OR 'mania'/de OR 'phobia'/de OR 'adjustment'/de OR 'reactive' OR dissociative OR 'illness anxiety' OR somatic OR neurocognitive OR 'delirium'/de OR 'social support'/de OR 'psychosocial support systems'/de OR 'interpersonal relations'/de OR 'social isolation'/de OR 'social adjustment'/de OR 'social participation'/de OR 'social behavior'/de OR 'sleep'/de OR 'sleeping'/de OR 'awake'/de OR 'insomnia'/de OR 'narcolepsy'/de OR 'sleep initiation and maintenance disorders'/de OR 'sleep latency'/de OR 'sleep wake disorders'/de OR

© 2020 Sultan P et al. *JAMA Network Open.*

'arousal'/de OR 'sexual health'/de OR 'reproductive health'/de OR 'coitus'/de OR 'intercourse'/de OR 'sexual dysfunctions' OR 'orgasm'/de OR 'dyspareunia'/de OR 'breast feeding'/de OR 'object attachment'/de OR 'infant care'/de OR 'bonding'/de AND ('instrumentation'/de OR 'methods'/de OR 'validation studies'/de OR 'comparative study'/de OR 'psychometrics'/de OR psychometr* OR clinimetr* OR clinometr* OR 'health care outcome assessment' OR 'outcome assessment'/de OR 'outcome measure*' OR 'observer variation'/de OR 'health status indicators'/de OR 'reproducibility of results'/de OR reproducib* OR 'discriminant analysis'/de OR reliab* OR unreliab* OR valid* OR 'coefficient of variation'/de OR coefficient OR 'homogeneity'/de OR homogeneous OR 'internal consistency'/de OR (cronbach* AND (alpha OR alphas)) OR (item AND (correlation* OR selection* OR reduction*)) OR 'agreement'/de OR 'precision'/de OR imprecision OR 'precise values' OR 'test retest' OR ('test'/de AND retest) OR (reliab* AND ('test'/de OR retest)) OR 'stability'/de OR interrater OR 'inter rater' OR intrarater OR 'intra rater' OR intertester OR 'inter tester' OR intratester OR 'intra tester' OR interobserver OR 'inter observer' OR intraobserver OR 'intra observer' OR intertechnician OR 'inter technician' OR intratechnician OR 'intra technician' OR interexaminer OR 'inter examiner' OR intraexaminer OR 'intra examiner' OR interassay OR 'inter assay' OR intraassay OR 'intra assay' OR interindividual OR 'inter individual' OR intraindividual OR 'intra individual' OR interparticipant OR 'inter participant' OR intraparticipant OR 'intra participant' OR kappa OR kappas OR repeatab* OR ((replicab* OR repeated) AND (measure OR measures OR findings OR result OR results OR 'test'/de OR tests)) OR generaliza* OR generalisa* OR 'concordance'/de OR (intraclass AND correlation*) OR discriminative OR 'known group' OR 'factor analysis'/de OR 'factor analyses' OR 'factor structure'/de OR 'factor structures' OR dimension* OR subscale* OR (multitrait AND 'scaling'/de AND ('analysis'/de OR analyses)) OR 'item discriminant' OR 'interscale correlation*' OR 'error'/de OR errors OR 'individual variability' OR 'interval variability' OR 'rate variability' OR ('variability'/de AND ('analysis'/de OR values)) OR ('uncertainty'/de AND ('measurement'/de OR measuring)) OR 'standard error of measurement'/de OR sensitiv* OR responsive* OR (limit AND 'detection'/de) OR 'minimal detectable concentration' OR interpretab* OR ((minimal OR minimally OR 'clinical'/de OR clinically) AND (important OR significant OR detectable) AND ('change'/de OR difference)) OR (small* AND (real OR detectable) AND ('change'/de OR difference)) OR 'meaningful change' OR 'ceiling effect'/de OR 'floor effect'/de OR 'item response model' OR irt OR rasch OR 'differential item functioning'/de OR dif OR 'computer adaptive testing'/de OR 'item bank' OR 'cross-cultural equivalence') NOT (('delphi technique'/de OR 'cross-sectional studies'/de OR 'addresses' OR 'biography'/de OR 'case reports' OR 'comment' OR 'directory'/de OR 'editorial'/de OR 'festschrift' OR 'interview'/de OR 'lectures' OR 'legal cases' OR 'legislation'/de OR 'letter'/de OR 'news' OR 'newspaper article' OR 'patient education handout' OR 'popular works' OR 'congresses'/de OR 'consensus development conference'/de OR 'nih consensus development conference' OR 'practice guideline'/de OR NOT ("Prenatal Care" OR "Prenatal Diagnosis" OR "Prenatal Education" OR prenatal OR antenatal OR 'animals'/de))
Filters: English

Total = 10,212
Endnote duplicate and animal removal = 8,585
Rayyan duplicate removal = 8,008

© 2020 Sultan P et al. *JAMA Network Open.*

eMethods 2. Development of Outpatient Recovery Domains (Adapted From Functional Recovery Domains Previously Described by Sharawi et al.[12])

Domains used by Sharawi et al. to describe functional recovery following cesarean delivery[12]

Physical (general)	Physical (Genitourinary / Gynecology / Fecal incontinence)^	Comfort & satisfaction*	Anesthesia side-effects*	Pain	Anesthesia & surgical complications^	Psychological^	Psychosocial & support^	Sleep	Sexual^	Nursing^	Cognition

Domains used to describe outpatient recovery following childbirth in this review (12; excluding global recovery measures)

Physical function	Surgical complications			Pain	Psychosocial distress ≠			Psychosocial support	Sleep	Motherhood experience ≠		Breast feeding / Breast health	Fatigue ≠	Sexual function	Scar and wound healing ≠	Cognition	(Global recovery)
	Urology	Gyne-cological	Colorectal		Psychological (other) ¥	Depression ¥ ≠	Anxiety ≠			Adapting to maternal role ≠	Maternal –neonatal bonding ≠						
Musculoskeletal and CVS deconditioning	Urinary Incontinence (type, urge, severity index)			Incision / wound	General health perception	Depression	Anxiety	Lifestyle / participation in community activities	Sleep quality	Caring for baby	Bonding	Breast feeding	Mental fatigue	Behavioral emotive	Appearance	Memory	> 3 domains assessing general health state rather than recovery related to specific domain
ADLs (personal hygiene, transferring)	Toileting (micturition)			Body	Emotional role / state			Social role / interaction with others	Latency	Parenting	Attachment	Lactation	Physical fatigue	Arousal	Healing	Critical thinking	
Physiological (physical independence, vitality)	Dysmenorrhea			Muscle	Emotional adoption			Social relationships with other people and function	Sleep quantity / change			Mastitis		Physical	Dehiscence	Problem solving	
Ability to hold baby	Uterine prolapse			Backache	Negative emotional experience			Support from family / staff / friends	Middle insomnia			Breast health		Partner related			
	Fecal incontinence			Headache	Mood			Psychological support	Terminal insomnia					Desire			
	Hemorrhoids			Sensory / affective	Enjoyment of life and fulfillment			Home / environment adaptation / maintenance of personal dignity	Insomnia					Lubrication			
	Anal fissure			Pelvic (not associated with menses or dyspareunia)	Mental health disorder (not depression)				Sleep medication					Orgasm			
				Physical discomfort	Motivation				Daytime sleepiness					Dyspareunia			
					Guilt / worthlessness				Sleep disturbance					Sex satisfaction			
					Helpless / hopelessness				Energy related to sleep								

© 2020 Sultan P et al. *JAMA Network Open.*

			Wellbeing				Nightmares								
			Psychomotor retardation (speech affected due to psychological reason)												
			In control / angry / confused												
			Appetite												
			Recued activity (psychological)												
			Living stress												
			Life satisfaction												
			Coping behavior												
			Embarrassment												
			Feeling alone												

^=domains re-classified or title amended; *=items removed from functional recovery following cesarean delivery domains, as they were felt not to be as relevant for assessment of outpatient recovery for the majority of patients. ¥=Certain aspects of psychological (other) domain are related to diagnosing depression. PROMs aimed at screening or diagnosing depression were included in the depression domain, whereas PROMs assessing a specific aspect of depression such as guilt / worthlessness were classified as psychological domain. ≠ new domains / sub-domains added to describe outpatient recovery in this review. CVS=cardiovascular; ADLs=activities of daily living.

Three domains were split into a total of 8 sub-domains. The domains of “anesthetic complications” and “anesthesia side-effects” were removed as these were felt to be less likely to affect women’s recovery following hospital discharge. Furthermore “anxiety” “depression” and “psychological” domains were grouped under the broad domain heading of “psychosocial distress” in order to acknowledge the overlapping nature of these conditions and frequency of psychiatric co-morbidity that exists within the community in the postpartum period. “Adaptation to maternal role” and “maternal-neonatal bonding” were also grouped under the broad domain of “motherhood experience” in order to acknowledge life-style and relationship adjustment required following delivery and the mother’s response to a new role. Finally, “fatigue,” and “scar and wound healing” were also added to the list of domains to include these aspects of outpatient postpartum recovery.

© 2020 Sultan P et al. *JAMA Network Open.*

eResults 1. References for the 515 Studies That Utilized Patient-Reported Outcome Measures (PROMs) to Evaluate Outpatient Recovery

1. Handa VL, Zyczynski HM, Burgio KL, et al. The impact of fecal and urinary incontinence on quality of life 6 months after childbirth. *Am J Obstet Gynecol.* 197(6):636.e631-636.
2. Abbasi M, van den Akker O, Bewley C. Persian couples' experiences of depressive symptoms and health-related quality of life in the pre- and perinatal period. *Journal of Psychosomatic Obstetrics and Gynecology.* 35(1):16-21.
3. Abdollahi F, Zarghami M. Effect of postpartum depression on women's mental and physical health four years after childbirth. *Eastern Mediterranean Health Journal.* 2018;24(10):1002-1009.
4. Abdollahi F, Zarghami M, Sazlina S-G, Zain AM, Mohammad AJ, Lye M-S. Prediction of incidence and bio-psycho-socio-cultural risk factors of post-partum depression immediately after birth in an Iranian population. *Archives of Medical Science.* 2016;12(5):1043-1051.
5. Abdollahpour S, Khosravi A, Bolbolhaghighi N. The effect of the magical hour on post-traumatic stress disorder (PTSD) in traumatic childbirth: a clinical trial. *Journal of Reproductive and Infant Psychology.* 34(4):403-412.
6. Acele EO, Karacam Z. Sexual problems in women during the first postpartum year and related conditions. *Journal of Clinical Nursing.* 21(7):929-937.
7. Adams SS, Eberhard-Gran M, vik AR, Eskild A. Mode of delivery and postpartum emotional distress: a cohort study of 55 814 women. *Bjog-an International Journal of Obstetrics and Gynaecology.* 119(3):298-305.
8. Adewuya AO, Ologun YA, Ibigbami OS. Post-traumatic stress disorder after childbirth in Nigerian women: Prevalence and risk factors. *BJOG: An International Journal of Obstetrics and Gynaecology.* 2006;113(3):284-288.
9. Ahlborg T, Dahlöf LG, Hallberg LRM. Quality of the intimate and sexual relationship in first-time parents six months after delivery. *Journal of Sex Research.* 2005;42(2):167-174.
10. Aksu S, Varol FG, Sahin NH. Long-term postpartum health problems in Turkish women: prevalence and associations with self-rated health. *Contemporary Nurse.* 53(2):167-181.
11. Aktan NM. Functional Status After Childbirth and Related Concepts. *Clinical Nursing Research.* 19(2):165-180.
12. Aktan NM. Social Support and Anxiety in Pregnant and Postpartum Women: A Secondary Analysis. *Clinical Nursing Research.* 21(2):183-194.
13. Aktan NM. *The relationship between social support, anxiety, and demographic variables and functional status after childbirth*, Rutgers The State University of New Jersey - Newark; 2009.
14. Ali NS, Ali BS, Azam IS. Post partum anxiety and depression in peri-urban communities of Karachi, Pakistan: a quasi-experimental study. *BMC Public Health.* 9:384.
15. Ali NS, Mahmud S, Khan A, Ali BS. Impact of postpartum anxiety and depression on child's mental development from two peri-urban communities of Karachi, Pakistan: a quasi-experimental study. *BMC Psychiatry.* 13:274.
16. Almutairi AF, Salam M, Alanazi S, Alweldawi M, Alsomali N, Alotaibi N. Impact of help-seeking behavior and partner support on postpartum depression among Saudi women. *Neuropsychiatric Disease and Treatment.* 2017;13:1929-1936.
17. Alves S, Fonseca A, Canavarro MC, Pereira M. Preliminary Psychometric Testing of

© 2020 Sultan P et al. *JAMA Network Open.*

the Postpartum Depression Predictors Inventory-Revised (PDPI-R) in Portuguese Women. *Maternal and Child Health Journal.* 22(4):571-578.
18. Anding JE, Rohrle B, Grieshop M, Schucking B, Christiansen H. Couple comorbidity and correlates of postnatal depressive symptoms in mothers and fathers in the first two weeks following delivery. *J Affect Disord.* 190:300-309.
19. Andreucci CB, Bussadori JC, Pacagnella RC, et al. Sexual life and dysfunction after maternal morbidity: A systematic review. *BMC Pregnancy and Childbirth.*15(1):307.
20. Andreucci CB, Cecatti JG, Pacagnella RC, et al. Does Severe Maternal Morbidity Affect Female Sexual Activity and Function? Evidence from a Brazilian Cohort Study. *Plos One.* 10(12):14.
21. Angelini DJ, Myers D, Raker C. Assessment of anal symptoms in primiparous women following spontaneous vaginal birth at term. *Journal of Midwifery & Women's Health.* 2011;56(5):526-526.
22. Arnott B, Brown A. An Exploration of Parenting Behaviours and Attitudes During Early Infancy: Association with Maternal and Infant Characteristics. *Infant and Child Development.* 22(4):349-361.
23. Arrue M, Diez-Itza I, Ibañez L, Paredes J, Murgiondo A. Changes in quality of life of women with stress urinary incontinence two years after first delivery. *International Urogynecology Journal and Pelvic Floor Dysfunction.* 2011;22:S169-S170.
24. Babineaux JU. Adolescent maternal competence and a postpartum home-based nursing intervention, Texas Woman's University; 1992.
25. Baghianimoghadam MH, Zadeh DS, Aminian AH. Caesarean Section, Vaginal Delivery and Post Natal Depression. *Iranian Journal of Public Health.* 2009;38(3):118-122.
26. Baghirzada L, Downey K, Macarthur A. Assessment of QOL indicators in the postpartum period. *Canadian Journal of Anesthesia.* 59:1-190.
27. Baghirzada L, Downey KN, Macarthur AJ. Assessment of quality of life indicators in the postpartum period. *International Journal of Obstetric Anesthesia.* 22(3):209-216.
28. Bahrami N, Karimian Z, Bahrami S, Bolbolhaghighi N. Comparing the Postpartum Quality of Life Between Six to Eight Weeks and Twelve to Fourteen Weeks After Delivery in Iran. *Iranian Red Crescent Medical Journal.* 16(7):5.
29. Bakker M, van der Beek AJ, Hendriksen IJM, Bruinvels DJ, van Poppel MNM. Predictive factors of postpartum fatigue: A prospective cohort study among working women. *Journal of Psychosomatic Research.* 77(5):385-390.
30. Bales M, Rascle N, Verdoux H, Sutter-Dallay AL. Characterization of perinatal mood disorders. *Archives of Women's Mental Health.* 2013;16:S85.
31. Barbara G, Pifarotti P, Facchin F, et al. Impact of Mode of Delivery on Female Postpartum Sexual Functioning: Spontaneous Vaginal Delivery and Operative Vaginal Delivery vs Cesarean Section. *Journal of Sexual Medicine.* 13(3):393-401.
32. Barkin JL, Willis GB, Hawkins KC, Stanfill-Thomas T, Beals L, Bloch JR. Semantic Assessment of the Barkin Index of Maternal Functioning in a Medically Underserved Obstetric Population. *Perspectives in Psychiatric Care.* 53(2):95-103.
33. Barkin JL, Wisner KL, Bromberger JT, Beach SR, Terry MA, Wisniewski SR. Development of the Barkin Index of Maternal Functioning. *Journal of Women's Health.* 19(12):2239-2246.
34. Barkin JL, Wisner KL, Bromberger JT, Beach SR, Terry MA, Wisniewski SR. The development of the barkin index of maternal functioning. *Archives of Women's Mental Health.* 2011;14:S8.
35. Barkin JL, Wisner KL, Wisniewski SR. The Psychometric Properties of the Barkin Index of Maternal Functioning. *Jognn-Journal of Obstetric Gynecologic and*

Neonatal Nursing. 43(6):792-802.
36. Barthel D, Kriston L, Barkmann C, et al. Longitudinal course of ante- and postpartum generalized anxiety symptoms and associated factors in West-African women from Ghana and Cote d'Ivoire. *J Affect Disord.* 197:125-133.
37. Bassi M, Delle Fave A, Cetin I, et al. Psychological well-being and depression from pregnancy to postpartum among primiparous and multiparous women. *Journal of Reproductive and Infant Psychology.* 2017;35(2):183-195.
38. Beck CT. Revision of the postpartum depression predictors inventory. *J Obstet Gynecol Neonatal Nurs.* 31(4):394-402.
39. Beck CT, Froman RD, Bernal H. Acculturation level and postpartum depression in Hispanic mothers. *MCN Am J Matern Child Nurs.* 30(5):299-304.
40. Beck CT, Gable RK. Postpartum depression screening scale: Spanish version. *Nurs Res.* 52(5):296-306.
41. Bell AF, Carter CS, Davis JM, et al. Childbirth and symptoms of postpartum depression and anxiety: a prospective birth cohort study. *Arch Women's Ment Health.* 19(2):219-227.
42. Berman RO. *Family adaptability, family cohesion, marital communication and the emerging family*, New York University; 1997.
43. Bijlenga D, Boers KE, Birnie E, et al. Maternal health-related quality of life after induction of labor or expectant monitoring in pregnancy complicated by intrauterine growth retardation beyond 36 weeks. *Qual Life Res.* 20(9):1427-1436.
44. Bijlenga D, Koopmans CM, Birnie E, et al. Health-related quality of life after induction of labor versus expectant monitoring in gestational hypertension or preeclampsia at term. *Hypertens Pregnancy.* 2011;30(3):260-274.
45. Birch M, Kulbel M. A dyadic model of treatment for postpartum support and education groups. *Archives of Women's Mental Health.* 2013;16:S107-S108.
46. Bjell, EK, Owe KM, et al. Pelvic pain after childbirth: a longitudinal population study. *Pain (03043959).* 2016;157(3):710-716.
47. Blasio PD, Camisasca E, Caravita SC, Ionio C, Milani L, Valtolina GG. The effects of expressive writing on postpartum depression and posttraumatic stress symptoms. *Psychol Rep.* 117(3):856-882.
48. Bo K, Hilde G, Tennfjord MK, Engh ME. Does episiotomy influence vaginal resting pressure, pelvic floor muscle strength and endurance, and prevalence of urinary incontinence 6 weeks postpartum? *Neurourol Urodyn.* 36(3):683-686.
49. Boateng GO, Martin SL, Tuthill EL, et al. Adaptation and psychometric evaluation of the breastfeeding self-efficacy scale to assess exclusive breastfeeding. *BMC Pregnancy Childbirth.* 19(1):73.
50. Boath E, Cox J, Lewis M, Jones P, Pryce A. When the cradle falls: the treatment of postnatal depression in a psychiatric day hospital compared with routine primary care. *J Affect Disord.* 53(2):143-151.
51. Bobevski I, Rowe H, Clarke DM, McKenzie DP, Fisher J. Early postnatal demoralisation among primiparous women in the community: measurement, prevalence and associated factors. *BMC Pregnancy Childbirth.* 15:259.
52. Borello-France D, Burgio KL, Richter HE, et al. Fecal and urinary incontinence in primiparous women. *Obstetrics & Gynecology.* 2006;108(4):863-872.
53. Botelho S, Alves FK, Vieira KJ, et al. Sex after the first pregnancy. *International Urogynecology Journal and Pelvic Floor Dysfunction.* 2012;23(2):S182-S183.
54. Botelho S, Silva JM, Palma P, Herrmann V, Riccetto C. Can the delivery method influence lower urinary tract symptoms triggered by the first pregnancy? *Int Braz J Urol.* 38(2):267-276.

© 2020 Sultan P et al. *JAMA Network Open.*

55. Botros SM, Abramov Y, Miller JJR, et al. Effect of parity on sexual function - An identical twin study. *Obstetrics and Gynecology.* 107(4):765-770.
56. Boufidou F, Lambrinoudaki I, Argeitis J, et al. CSF and plasma cytokines at delivery and postpartum mood disturbances. *J Affect Disord.* 115(1):287-292.
57. Boyce P, Hickie I, Parker G. Parents, partners or personality - risk-factors for postnatal depression. *Journal of Affective Disorders.* 21(4):245-255.
58. Boz I, Selvi N. Testing the Psychometric Properties of the Postpartum Sleep Quality Scale in Turkish Women. *Journal of Nursing Research.* 26(6):385-392.
59. Bozoky I, Corwin EJ. Fatigue as a predictor of postpartum depression. *Jognn-Journal of Obstetric Gynecologic and Neonatal Nursing.* 31(4):436-443.
60. Braga A, Sorice P, Furiga S, et al. Diastasis recti abdominis: Correlation with parity and stress urinary incontinence. *Neurourology and Urodynamics.* 2014;33:S8-S9.
61. Brown S, Bruinsma F, Darcy MA, Small R, Lumley J. Early discharge: no evidence of adverse outcomes in three consecutive population-based Australian surveys of recent mothers, conducted in 1989, 1994 and 2000. *Paediatric and Perinatal Epidemiology.* 18(3):202-213.
62. Brown S, Lumley J. Maternal health after childbirth: results of an Australian population based survey. *British Journal of Obstetrics and Gynaecology.* 105(2):156-161.
63. Brubaker L, a VL, Bradley CS, et al. Sexual function 6 months after first delivery. *Obstetrics & Gynecology.* 2008;111(5):1040-1044.
64. Buist A, Ellwood D, Brooks J, et al. National program for depression associated with childbirth: the Australian experience. *Best Practice & Research: Clinical Obstetrics & Gynaecology.* 2007;21(2):193-206.
65. Buist AE, Austin MPV, Hayes BA, et al. Postnatal mental health of women giving birth in Australia 2002-2004: findings from the beyondblue National Postnatal Depression Program. *Australian and New Zealand Journal of Psychiatry.* 2008;42(1):66-73.
66. Burgio MA, Lagana AS, Chille G, et al. Does epidural analgesia play a role in postpartum urinary incontinence? Medium-term results from a case-control study. *Journal of Maternal-Fetal & Neonatal Medicine.* 29(11):1773-1776.
67. Callahan JL, Borja SE, T Hynan M. Modification of the Perinatal PTSD Questionnaire to enhance clinical utility. *Journal of Perinatology.* 26(9):533-539.
68. Callahan JL, Hynan MT. Identifying mothers at risk for postnatal emotional distress: further evidence for the validity of the Perinatal Posttraumatic Stress Disorder Questionnaire. *Journal of Perinatology.* 2002;22(6):448-454.
69. Cano-Climent A, Oliver-Roig A, Cabrero-Garcia J, de Vries J, Richart-Martinez M. The Spanish version of the Fatigue Assessment Scale: reliability and validity assessment in postpartum women. *Peerj.* 5:15.
70. Carl, er AKK, Andolf E, Edman G, Wiklund I. Health-related quality of life five years after birth of the first child. *Sexual & Reproductive Healthcare.* 6(2):101-107.
71. Carter FA, Carter JD, Luty SE, et al. What is worse for your sex life: Starving, being depressed, or a new baby? *International Journal of Eating Disorders.* 40(7):664-667.
72. Cerro CR, Franco EM, Santoro GA, Palau MJ, Wieczorek P, Espuna-Pons M. Residual defects after repair of obstetric anal sphincter injuries and pelvic floor muscle strength are related to anal incontinence symptoms. *Int Urogynecol J.* 28(3):455-460.
73. Chang SR, Chang TC, Chen KH, Lin HH. Sexual Function in Women 3 Days and 6 Weeks After Childbirth: A Prospective Longitudinal Study Using the Taiwan Version of the Female Sexual Function Index. *Journal of Sexual Medicine.* 7(12):3946-3956.

74. Chayachinda C, Titapant V, Ungkanungdecha A. Dyspareunia and sexual dysfunction after vaginal delivery in Thai primiparous women with episiotomy. *J Sex Med.* 12(5):1275-1282.
75. Chen C. The prevalence of and psychosocial factors associated with postpartum depression of mothers in Taiwan, University of Pittsburgh;1994.
76. Chen CF, Gao JF, Zhang JJ, Jia LY, Yu TG, Zheng YZ. Serum leptin level measured 48 h after delivery is associated with development of postpartum depressive symptoms: a 3-month follow-up study. *Archives of Women's Mental Health.* 19(6):1001-1008.
77. Chen CH, Wang SY. Psychosocial outcomes of vaginal and cesarean births in Taiwanese primiparas. *Research in Nursing & Health.* 25(6):452-458.
78. Chen CM, Kuo SF, Chou YH, Chen HC. Postpartum Taiwanese women: their postpartum depression, social support and health-promoting lifestyle profiles. *Journal of Clinical Nursing.* 16(8):1550-1560.
79. Chin EG, Vincent C, Wilkie D. A comprehensive description of postpartum pain after cesarean delivery. *J Obstet Gynecol Neonatal Nurs.* 43(6):729-741.
80. Chinweuba AU, Okoronkwo IL, Anarado AN, Agbapuonwu NE, Ogbonnaya NP, Ihudiebube-Splendor CN. Differentials in health-related quality of life of employed and unemployed women with normal vaginal delivery. *BMC Women's Health.* 18(1):10.
81. Chivers ML, Pittini R, Grigoriadis S, Villegas L, Ross LE. The Relationship between Sexual Functioning and Depressive Symptomatology in Postpartum Women: A Pilot Study. *Journal of Sexual Medicine.* 8(3):792-799.
82. Chung FF, Wan GH, Kuo SC, Lin KC, Liu HE. Mother-infant interaction quality and sense of parenting competence at six months postpartum for first-time mothers in Taiwan: a multiple time series design. *BMC Pregnancy Childbirth.* 18(1):365.
83. Chung MY, Wan OY, Cheung RY, Chung TK, Chan SS. Prevalence of levator ani muscle injury and health-related quality of life in primiparous Chinese women after instrumental delivery. *Ultrasound Obstet Gynecol.* 45(6):728-733.
84. Clavenna A, Seletti E, Cartabia M, et al. Postnatal depression screening in a paediatric primary care setting in Italy. *Bmc Psychiatry.* 17(1):7.
85. Clevesy MA, Gatlin TK, Strebel KT. Is There a Relationship Between Gestational Diabetes and Perinatal Depression? *MCN-the American Journal of Maternal-Child Nursing.* 43(4):206-212.
86. Cohen LS, Viguera AC, Bouffard SM, et al. Venlafaxine in the treatment of postpartum depression. *Journal of Clinical Psychiatry.* 62(8):592-596.
87. Conradt E, Manian N, Bornstein MH. Screening for Depression in the Postpartum using the Beck Depression Inventory-II: What Logistic Regression Reveals. *J Reprod Infant Psychol.* 2012;30(5):427-435.
88. Coo S, Milgrom J, Kuppens P, Trinder J. Perinatal distress, an appraisal perspective. *Journal of Reproductive and Infant Psychology.* 33(2):190-204.
89. Coo-Calcagni S, Kuppens P, Trinder J, Milgrom J. Appraisal components of perinatal distress. *Archives of Women's Mental Health.* 2013;16:S113.
90. Cooklin AR, Giallo R, Rose N. Parental fatigue and parenting practices during early childhood: an Australian community survey. *Child Care Health and Development.* 38(5):654-664.
91. Cortes E, Basra R, Kelleher CJ. Waterbirth and pelvic floor injury: a retrospective study and postal survey using ICIQ modular long form questionnaires. *Eur J Obstet Gynecol Reprod Biol.* 155(1):27-30.
92. Cosimato C, Cipullo LM, Troisi J, et al. Ultrasonographic evaluation of urethrovesical

© 2020 Sultan P et al. *JAMA Network Open.*

junction mobility: correlation with type of delivery and stress urinary incontinence. *Int Urogynecol J.* 26(10):1495-1502.
93. Creti L, Tran D, Rizzo D, Zelkowitz P, Libman E. How do new mothers experience sleep? *Sleep Medicine.* 2015;16:S154-S155.
94. Cryan E, Keogh F, Connolly E, Cody S, Quinlan A, Daly I. Depression among postnatal women in an urban Irish community. *Irish Journal of Psychological Medicine.* 18(1):5-10.
95. Currie MJ, Reay RE, Thompson JF, Raphael B, Ellwood DA. Exercise and postnatal emotionalwellbeing: A randomised controlled trial comparing two exercise and support programs. *Archives of Women's Mental Health.* 2013;16:S12-S13.
96. Cyr M-P, Kruger J, Wong V, Dumoulin C, Girard I, Morin M. Pelvic floor morphometry and function in women with and without puborectalis avulsion in the early postpartum period. *American Journal of Obstetrics & Gynecology.* 2017;216(3):274.e271-274.e278.
97. Dagher RK, McGovern PM, Dowd BE. Maternity Leave Duration and Postpartum Mental and Physical Health: Implications for Leave Policies. *Journal of Health Politics, Policy & Law.* 2014;39(2):369-416.
98. Dagher RK, McGovern PM, Dowd BE, Gjerdingen DK. Postpartum Depression and Health Services Expenditures Among Employed Women. *Journal of Occupational and Environmental Medicine.* 54(2):210-215.
99. Dagher RK, McGovern PM, Dowd BE, Lundberg U. Postpartum depressive symptoms and the combined load of paid and unpaid work: a longitudinal analysis. *International Archives of Occupational and Environmental Health.* 84(7):735-743.
100. Dankner R, Goldberg RP, Fisch RZ, Crum RM. Cultural elements of postpartum depression - A study of 327 Jewish Jerusalem women. *Journal of Reproductive Medicine.* 45(2):97-104.
101. Dave B, Leader-Cramer A, Mueller MG, Johnson L, Kenton K, Lewicky-Gaupp C. Anal sphincter injuries after spontaneous vs operative delivery-is there a difference in postpartum symptoms? *Female Pelvic Medicine and Reconstructive Surgery.* 2015;21(5):S5-S6.
102. Dave BA, Leader-Cramer A, Mueller M, Johnson LL, Kenton K, Lewicky-Gaupp C. Anal Sphincter Injuries After Operative Vaginal Versus Spontaneous Delivery-Is There a Difference in Postpartum Symptoms? *Female Pelvic Medicine and Reconstructive Surgery.* 22(4):194-198.
103. De souza A, Dwyer PL, Charity M, Thomas E, Schierlitz L. A prospective study of sexual well being at 6 months postpartum. Does mode of delivery matter? *International Urogynecology Journal and Pelvic Floor Dysfunction.* 2012;23(2):S180-S181.
104. De Souza A, Dwyer PL, Charity M, Thomas E, Schierlitz L. A prospective study of sexual well being during pregnancy and the effects of breast feeding, depression and maternal BMI 6 months postpartum. *International Urogynecology Journal and Pelvic Floor Dysfunction.* 2012;23(2):S228-S229.
105. De souza AM, Dwyer PL, Thomas E, Charity M, Schierlitz L. A prospective study of sexual well being at 12 months postpartum. Does mode of delivery matter? *International Urogynecology Journal and Pelvic Floor Dysfunction*. 2013;24:S84.
106. Delpe SD, Russo A, Yeh J, et al. Influence of lower urinary tract symptoms on proficiency of urinary incontinence knowledge. *Female Pelvic Medicine and Reconstructive Surgery.* 2016;22(5):S69-S70.
107. Demirbas S, Atay V, Filiz AI, Sucullu I. Overlapping anal sphincter repair improves anal function in patients after obstetric and non-obstetric sphincter injury.

© 2020 Sultan P et al. *JAMA Network Open.*

Proktologia. 2006;7(3):176-185.
108. Demirchyan A, Petrosyan D, Armenian H. Rate and predictors of postpartum depression in a 22-year follow-up of a cohort of earthquake survivors in Armenia. *Archives of Women's Mental Health.* 2014;17(3):229-237.
109. Demitrack MA, Brock DG, Groom P, et al. Effectiveness of neurostar transcranial magnetic stimulation (TMS) in patients with major depressive disorder with postpartum onset. *Biological Psychiatry.* 2016;79(9):399S.
110. Denis A, Callahan S, Bouvard M. Evaluation of the French Version of the Multidimensional Scale of Perceived Social Support During the Postpartum Period. *Maternal and Child Health Journal.*19(6):1245-1251.
111. Denis A, Parant O, Callahan S. Post-traumatic stress disorder related to birth: a prospective longitudinal study in a French population. *Journal of Reproductive and Infant Psychology.* 2011;29(2):125-135.
112. Dennis C, Ross L. Relationships among infant sleep patterns, maternal fatigue, and development of depressive symptomatology. *Birth: Issues in Perinatal Care.* 2005;32(3):187-193.
113. Dennis CL. The process of developing and implementing a telephone-based peer support program for postpartum depression: evidence from two randomized controlled trials. *Trials.*15:8.
114. Dennis CL, Letourneau N. Global and relationship-specific perceptions of support and the development of postpartum depressive symptomatology. *Social Psychiatry and Psychiatric Epidemiology.* 42(5):389-395.
115. Dennis CL, McQueen K. Does maternal postpartum depressive symptomatology influence infant feeding outcomes? *Acta Paediatrica.* 96(4):590-594.
116. Dennis CLE. Identifying predictors of breastfeeding self-efficacy in the immediate postpartum period. *Research in Nursing & Health.* 29(4):256-268.
117. Des Rivieres-Pigeon C, Saurel-Cubizolles MJ, Romito P. Psychological distress one year after childbirth - A cross-cultural comparison between France, Italy and Quebec. *European Journal of Public Health.* 13(3):218-225.
118. Desseauve D, Proust S, Carlier-Guerin C, Rutten C, Pierre F, Fritel X. Evaluation of long-term pelvic floor symptoms after an obstetric anal sphincter injury (OASI) at least one year after delivery: A retrospective cohort study of 159 cases. *Gynecologie Obstetrique & Fertilite.* 44(7):385-390.
119. Di Dedda MC, Cantaluppi S, Sorice P, et al. Sexual disfunctions after childbirth and mode of delivery: Is there a correlation? *Neurourology and Urodynamics.* 2017;36:S49.
120. Di Florio A, Putnam K, Altemus M, et al. The impact of education, country, race and ethnicity on the self-report of postpartum depression using the Edinburgh Postnatal Depression Scale. *Psychological Medicine.* 47(5):787-799.
121. di Scalea TL, Hanusa BH, Wisner KL. Sexual Function in Postpartum Women Treated for Depression: Results From a Randomized Trial of Nortriptyline Versus Sertraline. *Journal of Clinical Psychiatry.* 70(3):423-428.
122. Dikmen-Yildiz P, Ayers S, Phillips L. Depression, anxiety, PTSD and comorbidity in perinatal women in Turkey: A longitudinal population-based study. *Midwifery.* 55:29-37.
123. Dikmen-Yildiz P, Ayers S, Phillips L. Screening for birth-related PTSD: psychometric properties of the Turkish version of the Posttraumatic Diagnostic Scale in postpartum women in Turkey. *Eur J Psychotraumatol.* 2017;8(1):1306414.
124. Ding G, Yu J, Vinturache A, Gu H, Lu M. Therapeutic effects of the traditional "Doing the month" practices on postpartum depression in China. *American Journal of*

© 2020 Sultan P et al. *JAMA Network Open.*

Psychiatry. 2018;175(11):1071-1072.
125. Ding T, Wang DX, Qu Y, Chen Q, Zhu SN. Epidural Labor Analgesia Is Associated with a Decreased Risk of Postpartum Depression: A Prospective Cohort Study. *Anesthesia and Analgesia.* 119(2):383-392.
126. Doering Runquist JJ, Morin K, Stetzer FC. Severe fatigue and depressive symptoms in lower-income urban postpartum women. *Western Journal of Nursing Research.* 2009;31(5):599-612.
127. Dogan B, Gun I, Ozdamar O, Yilmaz A, Muhcu M. Long-term impacts of vaginal birth with mediolateral episiotomy on sexual and pelvic dysfunction and perineal pain. *Journal of Maternal-Fetal & Neonatal Medicine.* 2017;30(4):457-460.
128. Dooher ME. Lamaze method of childbirth. *Nurs Res.* 29(4):220-224.
129. Dorheim SK, Bjorvatn B, Eberhard-Gran M. Can Insomnia in Pregnancy Predict Postpartum Depression? A Longitudinal, Population-Based Study. *Plos One.* 9(4):10.
130. Downe SM, Butler E, Hinder S. Screening tools for depressed mood after childbirth in UK-based South Asian women: a systematic review. *Journal of Advanced Nursing.* 57(6):565-583.
131. Ducarme G, Hamel JF, Brun S, Madar H, Merlot B, Sentilhes L. Sexual function and postpartum depression 6 months after attempted operative vaginal delivery according to fetal head station: A prospective population-based cohort study. *Plos One.* 12(6):17.
132. Dunn AB, Paul S, Ware LZ, Corwin EJ. Perineal Injury During Childbirth Increases Risk of Postpartum Depressive Symptoms and Inflammatory Markers. *Journal of Midwifery & Women's Health.* 60(4):428-436.
133. Durnea C, Carlson V, Khashan A, Kenny LC, O'Reilly B A. Prevalence of pelvic floor dysfunction in primiparous women at 1 year after delivery. *International Urogynecology Journal and Pelvic Floor Dysfunction.* 2011;22:S74-S75.
134. Durnea C, Khashan AS, Tabirca S, Kenny LC, O'Reilly BA. Prepregnancy pelvic floor dysfunction and postnatal pathology. *Reproductive Sciences.* 2013;20(3):336A.
135. Durnea CM, Khashan AS, Kenny LC, Durnea UA, O'Reilly B A. Risk factors for PFD in nulliparous women and in primiparous at 1 year postnatally. *Female Pelvic Medicine and Reconstructive Surgery.* 2014;20:S187-S188.
136. Durnea CM, Khashan AS, Kenny LC, Durnea UA, Smith MM, O'Reilly B A. Risk factors for postnatal pelvic organ prolapse in primiparous women. *International Urogynecology Journal and Pelvic Floor Dysfunction.* 2013;24:S128-S129.
137. Durnea CM, Khashan AS, Kenny LC, Tabirca SS, O'Reilly BA. The role of prepregnancy pelvic floor dysfunction in postnatal pelvic morbidity in primiparous women. *International Urogynecology Journal.* 25(10):1363-1374.
138. Easterbrooks MA, Bartlett JD, Raskin M, et al. Limiting home visiting effects: maternal depression as a moderator of child maltreatment. *Pediatrics.* 132:S126-133.
139. Eberhard-Gran M, Eskild A, Tambs K, Samuelsen SO, Opjordsmoen S. Depression in postpartum and non-postpartum women: prevalence and risk factors. *Acta Psychiatrica Scandinavica.* 106(6):426-433.
140. Eberhard-Gran M, Tambs K, Opjordsmoen S, Skrondal A, Eskild A. Depression during pregnancy and after delivery: A repeated measurement study. *Journal of Psychosomatic Obstetrics and Gynecology.* 25(1):15-21.
141. Eckerdal P, Georgakis MK, Kollia N, Wikstrom AK, Hogberg U, Skalkidou A. Delineating the association between mode of delivery and postpartum depression symptoms: a longitudinal study. *Acta Obstet Gynecol Scand.* 97(3):301-311.
142. Edhborg M. Comparisons of different instruments to measure blues and to predict depressive symptoms 2 months postpartum: a study of new mothers and fathers.

© 2020 Sultan P et al. *JAMA Network Open.*

Scandinavian Journal of Caring Sciences. 2008;22(2):186-195.
143. Edhborg M, Matthiesen AS, Lundh W, Widstrom AM. Some early indicators for depressive symptoms and bonding 2 months postpartum - a study of new mothers and fathers. *Archives of Women's Mental Health.* 8(4):221-231.
144. Edhborg M, Nasreen H-E, Kabir Z. Impact of postpartum depressive and anxiety symptoms on mothers' emotional tie to their infants 2-3 months postpartum: a population-based study from rural Bangladesh. *Archives of Women's Mental Health.* 2011;14(4):307-316.
145. Eid MA, Sayed A, Abdel-Rehim R, Mostafa T. Impact of the mode of delivery on female sexual function after childbirth. *International Journal of Impotence Research.* 27(3):118-120.
146. El-Heis S, Crozier SR, Healy E, et al. Maternal stress and psychological distress preconception: association with offspring atopic eczema at age 12 months. *Clinical and Experimental Allergy.* 47(6):760-769.
147. Elliott SA, Gerrard J, Ashton C, Cox JL. Training health visitors to reduce levels of depression after childbirth: an evaluation. *Journal of Mental Health.* 2001;10(6):613-625.
148. elzalts JE, Hairston IS, Matatyahu A. Construct Validity and Psychometric Properties of the Hebrew Version of the City Birth Trauma Scale. *Front Psychol.* 2018;9:1726.
149. Eriko T, Yasue K. The development of a maternal caregiving system: Based on changes in the attachment--caregiving balance scale up to 6-7 months postpartum. *Journal of Japan Academy of Midwifery.* 2013;27(2):237-246.
150. Escriba V, Mas R, Romito P, Saurel-Cubizolles MJ. Psychological distress of new Spanish mothers. *European Journal of Public Health.* 9(4):294-299.
151. Faltin DL, Boulvain M, Floris LA, et al. Diagnosis of anal sphincter tears to prevent fecal incontinence: a randomized controlled trial. *Obstetrics & Gynecology.* 106(1):6-13.
152. Fatoye FO, Adeyemi AB, Oladimeji BY. Postpartum depression following normal vaginal delivery among Nigerian women. *Psychol Rep.* 94(3):1276-1278.
153. Fatoye FO, Oladimeji BY, Adeyemi AB. Difficult delivery and some selected factors as predictors of early postpartum psychological symptoms among Nigerian women. *Journal of Psychosomatic Research.* 60(3):299-301.
154. Fatusic Z, Hudic I, Sinanovic O, Kapidzic M, Hotic N, Music A. Short-term postnatal quality of life in women with previous Misgav Ladach caesarean section compared to Pfannenstiel-Dorffler caesarean section method. *J Matern Fetal Neonatal Med.* 24(9):1138-1142.
155. Ferber SG, Granot M, Zimmer EZ. Catastrophizing labor pain compromises later maternity adjustments. *American Journal of Obstetrics and Gynecology.* 192(3):826-831.
156. Ferreira P, Gurl, B, Hull T, Zutshi M. Biologics in anal sphincter repair: To use or not to use. *Diseases of the Colon and Rectum.* 2010;53(4):566-567.
157. Ferreira Soares GC, de Almeida Andretto D, Grilo Diniz CS, Narhci NdZ. Adjustment disorders in the postpartum resulting from childbirth: a descriptive and exploratory study. *Online Brazilian Journal of Nursing.* 2012;11(3):907-922.
158. Fiala A, Švancara J, Klánová J, Kašpárek T. Sociodemographic and delivery risk factors for developing postpartum depression in a sample of 3233 mothers from the Czech ELSPAC study. *BMC Psychiatry.* 17(1):104.
159. Figueiredo B, Conde A. Anxiety and depression in women and men from early pregnancy to 3-months postpartum. *Arch Women's Ment Health.* 14(3):247-255.
160. Fishbein EG, Burggraf E. Early postpartum discharge: how are mothers managing?

JOGNN: Journal of Obstetric, Gynecologic & Neonatal Nursing. 1998;27(2):142-148.
161. Fisher J, Astbury J, Smith A. Adverse psychological impact of operative obstetric interventions: a prospective longitudinal study. *Australian and New Zealand Journal of Psychiatry.* 31(5):728-738.
162. Fisher J, Hammarberg K, Wynter K, et al. Childbirth and the first encounter with a newborn after assisted conception: Preliminary findings from the parental age and transition to parenthood Australia (PATPA) study. *Australian and New Zealand Journal of Obstetrics and Gynaecology.* 2009;49:A7.
163. Flaherman VJ, Gay B, Scott C, Aby J, Stewart AL, Lee KA. Development of the breast milk expression experience measure. *Maternal and Child Nutrition.* 2013;9(3):425-430.
164. Flink IK, Mroczek MZ, Sullivan MJ, Linton SJ. Pain in childbirth and postpartum recovery: the role of catastrophizing. *European Journal of Pain.* 2009;13(3):312-316.
165. Fritel X, Tsegan YE, Magnin G, Schweitzer M, Saurel-Cubizolles M. Is there a link between postpartum depression and urinary incontinence? A cohort study. *Neurourology and Urodynamics.* 2010;29(6):1143-1145.
166. Fu CW, Liu JT, Tu WJ, Yang JQ, Cao Y. Association between serum 25-hydroxyvitamin D levels measured 24hours after delivery and postpartum depression. *Bjog-an International Journal of Obstetrics and Gynaecology.* 122(12):1688-1694.
167. Furuta M, all J, Cooper D, Bick D. The relationship between severe maternal morbidity and psychological health symptoms at 6-8 weeks postpartum: a prospective cohort study in one English maternity unit. *Bmc Pregnancy and Childbirth.*14:14.
168. Gagnon LH, Boucher J, Robert M. Impact of pelvic floor muscle training in the postpartum period. *International Urogynecology Journal.* 27(2):255-260.
169. Ganann R, Sword W, Thabane L, Newbold B, Black M. Predictors of Postpartum Depression Among Immigrant Women in the Year After Childbirth. *Journal of Women's Health.* 25(2):155-165.
170. Gao LL, Chan SWC, You LM, Li XM. Experiences of postpartum depression among first-time mothers in mainland China. *Journal of Advanced Nursing.*66(2):303-312.
171. Gao X, Wang J, Yao H, Cai Y, Cheng R. Serum BDNF concentration after delivery is associated with development of postpartum depression: A 3-month follow up study. *Journal of Affective Disorders.* 2016;200:25-30.
172. Garthus-Niegel S, von Soest T, Vollrath ME, Eberhard-Gran M. The impact of subjective birth experiences on post-traumatic stress symptoms: a longitudinal study. *Archives of Women's Mental Health.* 16(1):1-10.
173. Gausia K, Ryder D, Ali M, Fisher C, Moran A, Koblinsky M. Obstetric Complications and Psychological Well-being: Experiences of Bangladeshi Women during Pregnancy and Childbirth. *Journal of Health Population and Nutrition.* 30(2):172-180.
174. Gebuza G, Kazmierczak M, Mieczkowska E, Gierszewska M. Social support as a determinant of life satisfaction in pregnant women and women after surgical delivery. *Psychiatr Pol.* 52(3):585-598.
175. Gelabert E, Subirà S, Garcia-Esteve L, et al. Perfectionism and other risk factors in postpartum depression: A case-control study. *European Psychiatry.* 2011;26.
176. Ghubash R, AbouSaleh MT. Postpartum psychiatric illness in Arab culture: Prevalence and psychosocial correlates. *British Journal of Psychiatry.*171:65-68.
177. Ghubash R, AbouSaleh MT, Daradkeh TK. The validity of the Arabic Edinburgh Postnatal Depression Scale. *Social Psychiatry and Psychiatric Epidemiology.* 32(8):474-476.

© 2020 Sultan P et al. *JAMA Network Open.*

178. Giallo R, Gartl, D, Woolhouse H, Brown S. Differentiating maternal fatigue and depressive symptoms at six months and four years post partum: Considerations for assessment, diagnosis and intervention. *Midwifery.* 31(2):316-322.
179. Glavin K, Smith L, Sorum R, Ellefsen B. Supportive counselling by public health nurses for women with postpartum depression. *Journal of Advanced Nursing.* 66(6):1317-1327.
180. Gonidakis F, Rabavilas AD, Varsou E, Kreatsas G, Christodoulou GN. A 6-month study of postpartum depression and related factors in Athens Greece. *Comprehensive Psychiatry.* 2008;49(3):275-282.
181. Gottlieb LN, Mendelson MJ. Mothers' moods and social support when a second child is born. *Maternal-Child Nursing Journal.* 1995;23(1):3-14.
182. Goulding AN, Grewen K, Meltzer-Brody S, Pearson B, Stuebe AM. 241: Maternal perception of birth trauma and its association with postpartum mood and parenting stress. *American Journal of Obstetrics and Gynecology.* 2019;220(1):S173.
183. Goyal D, Gay C, Lee K. Fragmented maternal sleep is more strongly correlated with depressive symptoms than infant temperament at three months postpartum. *Archives of Women's Mental Health.* 12(4):229-237.
184. Goyal K, Purbiya P, Lal SN, Kaur J, Anthwal P, Puliyel JM. Correlation of Infant Gender with Postpartum Maternal and Paternal Depression and Exclusive Breastfeeding Rates. *Breastfeeding Medicine.* 2017;12(5):279-282.
185. Gregoire AJ, Kumar R, Everitt B, Henderson AF, Studd JW. Transdermal oestrogen for treatment of severe postnatal depression. *Lancet.* 347(9006):930-933.
186. Grice MM, Feda D, McGovern P, et al. Giving birth and returning to work: The impact of work-family conflict on women's health after childbirth. *Annals of Epidemiology.* 17(10):791-798.
187. Grussu P, Quatraro RM. Prevalence and risk factors for a high level of postnatal depression symptomatology in Italian women: A sample drawn from ante-natal classes. *European Psychiatry.* 24(5):327-333.
188. Grussu P, Quatraro RM. Maternity Blues in Italian Primipara Women: Symptoms and Mood States in the First Fifteen Days After Childbirth. *Health Care for Women International.* 2013;34(7):556-576.
189. Grylka-Baeschlin S, van Teijlingen E, Gross MM. Cultural differences in postnatal quality of life among German-speaking women - a prospective survey in two countries. *BMC Pregnancy and Childbirth.* 14(1):277.
190. Gureje O, Oladeji BD, Araya R, et al. Expanding care for perinatal women with depression (EXPONATE): study protocol for a randomized controlled trial of an intervention package for perinatal depression in primary care. *BMC Psychiatry.*15:9.
191. Gursen C, Inanoglu D, Kaya S, Akbayrak T, Baltaci G. Effects of exercise and Kinesio taping on abdominal recovery in women with cesarean section: a pilot randomized controlled trial. *Arch Gynecol Obstet.* 293(3):557-565.
192. Guzman Rojas R, Wong V, Shek KL, Dietz HP. Impact of levator trauma on pelvic floor muscle function. *Int Urogynecol J.* 25(3):375-380.
193. Haddad-Rodrigues M, Márcia Spanó Nakano A, Stefanello J, Campos Pereira Silveira RC. Acupuncture for Anxiety in Lactating Mothers with Preterm Infants: A Randomized Controlled Trial. *Evidence-based Complementary & Alternative Medicine (eCAM).* 2013;2013:1-9.
194. Haga SM, Ulleberg P, Slinning K, Kraft P, Steen T, Staff AC. A longitudinal study of postpartum depression: Multilevel growth curve analyses of emotion regulation strategies, breastfeeding self-efficacy and social support. *Archives of Women's Mental Health.* 2013;16:S123.

© 2020 Sultan P et al. *JAMA Network Open.*

195. Hairston IS, elzalts JE, Assis C, Kovo M. Postpartum bonding difficulties and adult attachment styles: the mediating role of postpartum depression and childbirth-related PTSD. *Infant Mental Health Journal.* 39(2):198-208.
196. Hall PL, Papageorgiou C. Negative thoughts after childbirth: Development and preliminary validation of a self-report scale. *Depression and Anxiety.* 2005;22(3):121-129.
197. Halperin O, Noble A, Balachsan S, Klug E, Liebergall-Wischnitzer M. Association between severities of striae gravidarum and Obstetric Anal Sphincter Injuries (OASIS). *Midwifery.* 54:25-28.
198. Hanusa BH, Scholle SH, Haskett RF, Spadaro K, Wisner KL. Screening for depression in the postpartum period: A comparison of three instruments. *Journal of Women's Health.* 17(4):585-596.
199. Harris B, Othman S, Davies JA, et al. Association between postpartum thyroid-dysfunction and thyroid antibodies and depression. *BMJ-British Medical Journal.* 305(6846):152-156.
200. Harvey MA, Johnston SL, Davies GAL. Mid-trimester serum relaxin concentrations and post-partum pelvic floor dysfunction. *Acta Obstetricia et Gynecologica Scandinavica.* 2008;87(12):1315-1321.
201. Hastings V, McCallister AM, Curtis SA, Valant RJ, Yao S. Efficacy of Osteopathic Manipulative Treatment for Management of Postpartum Pain. *J Am Osteopath Assoc.* 116(8):502-509.
202. Hellmers C, Schuecking B. Primiparae's well-being before and after birth and relationship with preferred and actual mode of birth in Germany and the USA. *Journal of Reproductive and Infant Psychology.* 2008;26(4):351-372.
203. Henry Folcarelli P. An evaluation of the influence of social support and self-esteem on physical and mental health in postpartum mothers, New York University; 2004.
204. Henshaw C, Foreman D, Cox J. Postnatal blues: a risk factor for postnatal depression. *J Psychosom Obstet Gynaecol.* 25(3):267-272.
205. Heron J, Haque S, Oyebode F, Craddock N, Jones I. A longitudinal study of hypomania and depression symptoms in pregnancy and the postpartum period. *Bipolar Disord.* 11(4):410-417.
206. Hill PD, Aldag JC. Maternal perceived quality of life following childbirth. *Jognn-Journal of Obstetric Gynecologic and Neonatal Nursing.* 36(4):328-334.
207. Hills NF, Graham RB, McLean L. Comparison of Trunk Muscle Function Between Women With and Without Diastasis Recti Abdominis at 1 Year Postpartum. *Physical Therapy.*98(10):891-901.
208. Hiltunen P, Raudaskoski T, Ebeling H, Moilanen I. Does pain relief during delivery decrease the risk of postnatal depression? *Acta Obstetricia Et Gynecologica Scandinavica.* 83(3):257-261.
209. Hiscock H, Bayer J, Gold L, Hampton A, Ukoumunne OC, Wake M. Improving infant sleep and maternal mental health: a cluster randomised trial. *Archives of Disease in Childhood.* 92(11):952-958.
210. Hoedjes M, Berks D, Vogel I, et al. Poor Health-related Quality of Life After Severe Preeclampsia. *Birth-Issues in Perinatal Care.* 38(3):246-255.
211. Hoffenkamp HN, Tooten A, Hall RA, et al. The impact of premature childbirth on parental bonding. *Evol Psychol.* 10(3):542-561.
212. Holden JM. Postnatal depression: its nature, effects, and identification using the Edinburgh Postnatal Depression Scale. *Birth: Issues in Perinatal Care.* 1991;18(4):211-221.
213. Hosseini L, Iran-Pour E, Safarinejad MR. Sexual Function of Primiparous Women

© 2020 Sultan P et al. *JAMA Network Open.*

After Elective Cesarean Section and Normal Vaginal Delivery. *Urology Journal.* 9(2):498-504.
214. Hou YM, Hu PC, Zhang YM, et al. Cognitive behavioral therapy in combination with systemic family therapy improves mild to moderate postpartum depression. *Revista Brasileira De Psiquiatria.* 36(1):47-52.
215. House SJ, Tripathi SP, Knight BT, Morris N, Newport DJ, Stowe ZN. Obsessive-compulsive disorder in pregnancy and the postpartum period: course of illness and obstetrical outcome. *Arch Women's Ment Health.* 19(1):3-10.
216. Houston KA, Kaimal AJ, Nakagawa S, Gregorich SE, Yee LM, Kuppermann M. Mode of delivery and postpartum depression: the role of patient preferences. *Am J Obstet Gynecol.* 212(2):229.e221-227.
217. Howell EA, Balbierz A, Wang J, Parides M, Zlotnick C, Leventhal H. Reducing Postpartum Depressive Symptoms Among Black and Latina Mothers A Randomized Controlled Trial. *Obstetrics and Gynecology.* 119(5):942-949.
218. Huang CM, Carter PA, Guo JL. A comparison of sleep and daytime sleepiness in depressed and non-depressed mothers during the early postpartum period. *J Nurs Res.* 12(4):287-296.
219. Huang YC, Mathers NJ. A comparison of sexual satisfaction and post-natal depression in the UK and Taiwan. *Int Nurs Rev.* 53(3):197-204.
220. Hung CH. Postpartum stress as a predictor of women's minor psychiatric morbidity. *Community Mental Health Journal.* 43(1):1-12.
221. Hung CH. Measuring postpartum stress. *Journal of Advanced Nursing.* 2005;50(4):417-424.
222. Hung CH, Chung HH. The effects of postpartum stress and social support on postpartum women's health status. *Journal of Advanced Nursing.* 36(5):676-684.
223. Hung CH, Lin CJ, Stocker J, Yu CY. Predictors of postpartum stress. *Journal of Clinical Nursing.* 20(5):666-674.
224. Hung HM, Chen CH. Sleep Quality in Postpartum Women: Exploring Correlation With Childbirth Experience and Household Work. *Journal of Nursing Research.* 22(1):20-27.
225. Huppelschoten AG, van Ginderen JC, van den Broek KC, Bouwma AE, Oosterbaan HP. Different ways of subcutaneous tissue and skin closure at cesarean section: a randomized clinical trial on the long-term cosmetic outcome. *Acta Obstet Gynecol Scand.* 92(8):916-924.
226. Hutton EK, Hannah ME, Ross S, et al. Maternal 3 month outcomes after planned cesarean (CS) vs planned vaginal birth (VB) for twin pregnancies: The twin birth study (TBS). *Reproductive Sciences.* 2014;21(3):284A.
227. Hutton EK, Hannah ME, Ross S, et al. Maternal outcomes at 3 months after planned caesarean section versus planned vaginal birth for twin pregnancies in the Twin Birth Study: a randomised controlled trial. *BJOG: An International Journal of Obstetrics & Gynaecology.* 2015;122(12):1653-1662.
228. il S, Jabr S, Wagler S, Collin SM. Postpartum depression in the Occupied Palestinian Territory: a longitudinal study in Bethlehem. *BMC Pregnancy Childbirth.*16(1):375.
229. Iliadis S, Koulouris P, Gingnell M, et al. Personality and risk for postpartum depressive symptoms. *Archives of Women's Mental Health.* 2015;18(3):539-546.
230. Iliadis SI, Comasco E, Sylvén S, Hellgren C, Poromaa IS, Skalkidou A. Prenatal and postpartum evening salivary cortisol levels in association with peripartum depressive symptoms. *PLoS ONE.* 2015;10(8):e0135471.
231. Iliadis SI, Skalkidou A, Ranstr, et al. Self-Harm Thoughts Postpartum as a Marker for Long-Term Morbidity. *Frontiers in Public Health.*6:9.

© 2020 Sultan P et al. *JAMA Network Open.*

232. Imsiragic AS, Begic D, Sarajlic I, Palavra IR, Orban M. Predictors of Exclusive Breastfeeding 6-9 Weeks After Delivery: a Prospective Cohort Study. Public Mental Health Perspective. *Psychiatr Danub.* 28(4):395-403.
233. Imsiragic AS, Begic D, Simicevic L, Bajic Z. Prediction of posttraumatic stress disorder symptomatology after childbirth - A Croatian longitudinal study. *Women and Birth.* 30(1):E17-E23.
234. Imsiragic AS, Begic D, Vukovic IS, Simicevic L, Javorina T. Multivariate analysis of predictors of depression symptomatology after childbirth. *Psychiatria Danubina.* 2014;26:416-421.
235. İnan C, Ağır MÇ, Sağır FG, et al. Assessment of the effects of perineoplasty on female sexual function. *Balkan Medical Journal.* 2015;32(3):260-265.
236. Ionio C, Di Blasio P. Post-traumatic stress symptoms after childbirth and early mother–child interactions: an exploratory study. *Journal of Reproductive & Infant Psychology.* 2014;32(2):163-181.
237. Ishida K, Stupp P, Serbanescu F, Tullo E. Perinatal risk for common mental disorders and suicidal ideation among women in Paraguay. *International Journal of Gynecology & Obstetrics.* 110(3):235-240.
238. Ishikawa N, Goto S, Murase S, et al. Prospective study of maternal depressive symptomatology among Japanese women. *Journal of Psychosomatic Research.* 71(4):264-269.
239. Izard CE, Libero DZ, Putnam P, Haynes OM. Stability of emotion experiences and their relations to traits of personality. *Journal of Personality and Social Psychology.* 64(5):847-860.
240. Jahanfar S, Howard LM, Medley N. Interventions for preventing or reducing domestic violence against pregnant women. *Cochrane Database of Systematic Reviews.* 2014(11):65.
241. Jamaliv, S, Char, abi SMA, Mirghafourv, M. Comparing the Effect of Electronic Software and Training Booklet on Maternal Self-Confidence and Awareness About Newborn Care: A Randomized Controlled Clinical Trial. *Iranian Red Crescent Medical Journal.* 19(4):9.
242. Jansen AJ, Duvekot JJ, Hop WC, et al. New insights into fatigue and health-related quality of life after delivery. *Acta Obstet Gynecol Scand.* 2007;86(5):579-584.
243. Jansen AJ, Essink-Bot ML, Duvekot JJ, van Rhenen DJ. Psychometric evaluation of health-related quality of life measures in women after different types of delivery. *J Psychosom Res.* 63(3):275-281.
244. Jawed-Wessel S, Schick V, Herbenick D. The Sexual Function Questionnaire's Medical Impact Scale (SFQ-MIS): Validation Among a Sample of First-time Mothers. *Journal of Sexual Medicine.* 10(11):2715-2722.
245. Ji S, Long Q, Newport DJ, et al. Validity of depression rating scales during pregnancy and the postpartum period: impact of trimester and parity. *J Psychiatr Res.* 45(2):213-219.
246. Johannessen HH, Stafne SN, Falk RS, Stordahl A, Wibe A, Morkved S. Prevalence and predictors of double incontinence 1 year after first delivery. *Int Urogynecol J.* 29(10):1529-1535.
247. Johannessen HH, Wibe A, Stordahl A, et al. Do pelvic floor muscle exercises reduce postpartum anal incontinence? A randomised controlled trial. *BJOG: An International Journal of Obstetrics & Gynaecology.* 2017;124(4):686-694.
248. Johansson M, Svensson I, Stenstrom U, Massoudi P. Depressive symptoms and parental stress in mothers and fathers 25 month after birth. *Journal of Child Health Care.* 21(1):65-73.

© 2020 Sultan P et al. *JAMA Network Open.*

249. Jonas W, Nissen E, Ransjo-Arvidson A, Matthiesen A, Uvnas-Moberg K. Influence of oxytocin or epidural analgesia on personality profile in breastfeeding women: a comparative study. *Archives of Women's Mental Health.* 11(5):335-345.
250. Jordan PA, Naidu M, Thakar R, Sultan AH. Effect of subsequent vaginal delivery on bowel symptoms and anorectal function in women who sustained a previous obstetric anal sphincter injury. *Int Urogynecol J.* 29(11):1579-1588.
251. Josefsson A, Larsson C, Sydsjo G, Nyl, er PO. Temperament and character in women with postpartum depression. *Arch Women's Ment Health.* 10(1):3-7.
252. Kadir AA, Nordin R, Ismail SB, Yaacob MJ, Mustapha WMR. Postnatal depression in mothers attending primary care clinics in Kelantan, Malaysia. *International Medical Journal.* 2005;12(2):105-109.
253. Kahramanoglu I, Baktiroglu M, Hamzaoglu K, et al. The impact of mode of delivery on the sexual function of primiparous women: a prospective study. *Archives of Gynecology & Obstetrics.* 2017;295(4):907-916.
254. Kahyaoglu Sut H, Balkanli Kaplan P. Effect of pelvic floor muscle exercise on pelvic floor muscle activity and voiding functions during pregnancy and the postpartum period. *Neurourol Urodyn.* 35(3):417-422.
255. Kaitz M, Stecklov G, Devor N. Anxiety symptoms of new mothers during a period of recurrent, local terror. *J Affect Disord.* 107(1):211-215.
256. Kakyo TA, Muliira JK, Mbalinda SN, Kizza IB, Muliira RS. Factors associated with depressive symptoms among postpartum mothers in a rural district in Uganda. *Midwifery.* 2012;28(3):374-379.
257. Kalis V, smanova J, Bednarova B, Karbanova J, Laine K, Rokyta Z. Evaluation of the incision angle of mediolateral episiotomy at 60 degrees. *International Journal of Gynecology & Obstetrics.* 112(3):220-224.
258. Karacam Z, Coban A, Akbas B, Karabulut E. Status of postpartum depression in Turkey: A meta-analysis. *Health Care Women Int.* 39(7):821-841.
259. Kastikainen J, Ballen N, hi J, et al. Universal screening for PPD: Identifying the gaps in care delivery for at-risk populations. *Archives of Women's Mental Health.* 2013;16:S53-S54.
260. Kemppinen K, Kumpulainen K, Moilanen I, Ebeling H. Recurrent and transient depressive symptoms around delivery and maternal sensitivity. *Nord J Psychiatry.* 2006;60(3):191-199.
261. Kermode M, Fisher J, Jolley D. Health insurance status and mood during pregnancy and following birth: a longitudinal study of multiparous women. *Australian and New Zealand Journal of Psychiatry.* 34(4):664-670.
262. Khajehei M. Prevalence and Risk Factors of Relationship Dissatisfaction in Women During the First Year After Childbirth: Implications for Family and Relationship Counseling. *Journal of Sex & Marital Therapy.* 2016;42(6):484-493.
263. Khalifa DS, Glavin K, Bjertness E, Lien L. Course of depression symptoms between 3 and 8 months after delivery using two screening tools (EPDS and HSCL-10) on a sample of Sudanese women in Khartoum state. *BMC Pregnancy Childbirth.* 18(1):324.
264. Kim TH, Connolly JA, Tamim H. The effect of social support around pregnancy on postpartum depression among Canadian teen mothers and adult mothers in the maternity experiences survey. *BMC Pregnancy Childbirth.* 14:162.
265. Kindberg S, Stehouwer M, Hvidman L, Henriksen TB. [Commentary on] Postpartum perineal repair performed by midwives: a randomized trial comparing two suture techniques leaving the skin unsutured...Including commentary by Rouse DJ. *Obstetrical & Gynecological Survey.* 2008;63(7):416-417.

© 2020 Sultan P et al. *JAMA Network Open.*

266. Klein K, Worda C, Leipold H, Gruber C, Husslein P, Wenzl R. Does the Mode of Delivery Influence Sexual Function after Childbirth? *Journal of Women's Health.* 18(8):1227-1231.
267. Koc O, Duran B, Ozdemirci S, Bakar Y, Ozengin N. Is cesarean section a real panacea to prevent pelvic organ disorders? *International Urogynecology Journal.* 22(9):1135-1141.
268. Koenigseder LA. Patterns of change in primiparas' moods and functional status: an extension of Rubin's nursing model, University Of Texas at Austin; 1991.
269. Kohler S, Sidney Annerstedt K, Diwan V, et al. Postpartum quality of life in Indian women after vaginal birth and cesarean section: a pilot study using the EQ-5D-5L descriptive system. *BMC Pregnancy Childbirth.* 18(1):427.
270. Komatsu R, Carvalho B, Flood P. Prediction of outliers in pain, analgesia requirement, and recovery of function after childbirth: a prospective observational cohort study. *British Journal of Anaesthesia.* 121(2):417-426.
271. Koo V, Lynch J, Cooper S. Risk of postnatal depression after emergency delivery. *Journal of Obstetrics and Gynaecology Research.* 29(4):246-250.
272. Kossakowska K. Psychological risk factors of postpartum depression among high-risk pregnancy women. *Archives of Women's Mental Health.* 2013;16:S126.
273. Koutra K, Vassilaki M, Georgiou V, et al. Pregnancy, perinatal and postpartum complications as determinants of postpartum depression: The Rhea mother-child cohort in Crete, Greece. *Epidemiology and Psychiatric Sciences.* 2018;27(3):244-255.
274. Koyasu K, Kinkawa M, Ueyama N, Tanikawa Y, Adachi K, Matsuo H. The prevalence of primary neck and shoulder pain, and its related factors in Japanese postpartum women. *Clinical and Experimental Obstetrics & Gynecology.* 2015;42(1):5-10.
275. Kozinszky Z, Dudas RB, Csatordai S, et al. Social dynamics of postpartum depression: a population-based screening in South-Eastern Hungary. *Social Psychiatry and Psychiatric Epidemiology.* 46(5):413-423.
276. Ku CM, Chow SKY. Factors influencing the practice of exclusive breastfeeding among Hong Kong Chinese women: a questionnaire survey. *Journal of Clinical Nursing.*19(17):2434-2445.
277. Kubota C, Okada T, Morikawa M, et al. Postpartum depression among women in Nagoya indirectly exposed to the Great East Japan Earthquake. *Scientific Reports.* 8(1):6.
278. Kudo N, Shinohara H, Kodama H. Heart Rate Variability Biofeedback Intervention for Reduction of Psychological Stress During the Early Postpartum Period. *Applied Psychophysiology and Biofeedback.* 39(3):203-211.
279. Kuo SY, Yang YL, Kuo PC, Tseng CM, Tzeng YL. Trajectories of Depressive Symptoms and Fatigue Among Postpartum Women. *Jognn-Journal of Obstetric Gynecologic and Neonatal Nursing.* 41(2):216-226.
280. Lagaert L, Weyers S, Van Kerrebroeck H, Elaut E. Postpartum dyspareunia and sexual functioning: a prospective cohort study. *European Journal of Contraception and Reproductive Health Care.* 2017;22(3):200-206.
281. Lagana AS, Burgio MA, Ciancimino L, et al. Evaluation of recovery and quality of sexual activity in women during postpartum in relation to the different mode of delivery: a retrospective analysis. *Minerva Ginecol.* 67(4):315-320.
282. Lavender T, Walkinshaw SA. Can midwives reduce postpartum psychological morbidity? A randomized trial. *Birth-Issues in Perinatal Care.* 25(4):215-219.
283. Lawrence CL, Norris AE. Psychometric Properties of an Instrument to Measure Mother-Infant Togetherness After Childbirth. *J Nurs Meas.* 2016;24(1):108-130.

© 2020 Sultan P et al. *JAMA Network Open.*

284. Lawrie TA, Hofmeyr GJ, De Jager M, Berk M, Paiker J, Viljoen E. A double-blind randomised placebo controlled trial of postnatal norethisterone enanthate: the effect on postnatal depression and serum hormones. *British Journal of Obstetrics and Gynaecology.* 105(10):1082-1090.
285. Leahy-Warren P, McCarthy G, Corcoran P. First-time mothers: social support, maternal parental self-efficacy and postnatal depression. *Journal of Clinical Nursing.* 21(3):388-397.
286. LeCheminant JD, Hinman T, Pratt KB, et al. Effect of resistance training on body composition, self- efficacy, depression, and activity in postpartum women. *Scandinavian Journal of Medicine & Science in Sports.* 24(2):414-421.
287. Lecompte V, Rousseau C. Determinants of Child Attachment in the Years Postpartum in a High-Risk Sample of Immigrant Women. *Journal of Immigrant and Minority Health.* 20(5):1166-1172.
288. Lee DT, Yip AS, Chiu HF, Chung TK. Screening for postnatal depression using the double-test strategy. *Psychosom Med.* 62(2):258-263.
289. Lee IS, Choi ES. Pelvic floor muscle exercise by biofeedback and electrical stimulation to reinforce the pelvic floor muscle after normal delivery. *Taehan Kanho Hakhoe Chi.* 36(8):1374-1380.
290. Levy V. The maternity blues in post-partum and post-operative women. *Br J Psychiatry.* 151:368-372.
291. Li ZY, Xu T, Li ZA, Gong J, Liu Q, Zhu L. Lower urinary tract symptoms 7 years after the first delivery: Correlation to the mode of delivery. *Neurourology and Urodynamics.* 38(2):793-800.
292. Lilja G, Edhborg M, Nissen E. Depressive mood in women at childbirth predicts their mood and relationship with infant and partner during the first year postpartum. *Scandinavian Journal of Caring Sciences.* 26(2):245-253.
293. Lim G, Farrell LM, Facco FL, Gold MS, Wasan AD. Labor Analgesia as a Predictor for Reduced Postpartum Depression Scores: A Retrospective Observational Study. *Anesthesia and Analgesia.* 126(5):1598-1605.
294. Lim G, Farrell LM, Nam S, Wasan AD. Moderation-mediation effects between labor and postpartum pain, prenatal factors, and postpartum depression. *Journal of Women's Health.* 2018;27(11):1424-1425.
295. Lin YH, Chang SD, Hsieh WC, et al. Persistent stress urinary incontinence during pregnancy and one year after delivery; its prevalence, risk factors and impact on quality of life in Taiwanese women: An observational cohort study. *Taiwanese Journal of Obstetrics & Gynecology.* 57(3):340-345.
296. Ling-ling G, Wen X, Xiao Y, Wai-chi Chan S. Effects of an interpersonal-psychotherapy-oriented postnatal programme for Chinese first-time mothers: A randomized controlled trial. *International Journal of Nursing Studies.* 2015;52(1):22-29.
297. Liu SP, Yan Y, Gao X, et al. Risk factors for postpartum depression among Chinese women: path model analysis. *Bmc Pregnancy and Childbirth.* 17(1):7.
298. Liu Y, Chen X, Guo AH, Lv CX. Effect of extended nursing intervention on improvement of anxiety in puerperal patients. *International Journal of Clinical and Experimental Medicine.* 2018;11(12):13033-+.
299. Lo J, Osterweil P, Li H, Mori T, Eden KB, Guise JM. Quality of Life in Women With Postpartum Anal Incontinence. *Obstetrics and Gynecology.* 115(4):809-814.
300. Logsdon MC. Initial psychometric properties of the Inventory of Functional Status after Childbirth -- Revised for Adolescents. *Journal of Reproductive & Infant Psychology.* 2009;27(4):346-356.

© 2020 Sultan P et al. *JAMA Network Open.*

301. Lommatzsch M, Hornych K, Zingler C, Schuff-Werner P, Hoppner J, Virchow JC. Maternal serum concentrations of BDNF and depression in the perinatal period. *Psychoneuroendocrinology.* 31(3):388-394.
302. Lopez-Lapeyrere C, Serna-Gomez N, Hern, et al. The development and validation of a new postpartum sexual function and dyspareunia assessment tool: The Carol Scale. *Midwifery.* 58:27-36.
303. Lung FW, Shu BC, Chiang TL, Lin SJ. Parental mental health, education, age at childbirth and child development from six to 18 months. *Acta Paediatrica.* 98(5):834-841.
304. Luoma I, Puura K, Mantymaa M, Latva R, Salmelin R, Tamminen T. Fathers' postnatal depressive and anxiety symptoms: An exploration of links with paternal, maternal, infant and family factors. *Nordic Journal of Psychiatry.* 67(6):407-413.
305. Lydon-Rochelle MT, Holt VL, Martin DP. Delivery method and self-reported postpartum general health status among primiparous women. *Paediatric and Perinatal Epidemiology.* 15(3):232-240.
306. MacArthur C, Wilson D, Herbison P, et al. Urinary incontinence persisting after childbirth: extent, delivery history, and effects in a 12-year longitudinal cohort study. *Bjog-an International Journal of Obstetrics and Gynaecology.* 123(6):1022-1029.
307. MacArthur C, Winter HR, Bick DE, et al. Effects of redesigned community postnatal care on womens' health 4 months after birth: a cluster randomised controlled trial. *Lancet.* 359(9304):378-385.
308. Machado MC, Assis KF, Oliveira Fde C, et al. Determinants of the exclusive breastfeeding abandonment: psychosocial factors. *Rev Saude Publica.* 48(6):985-994.
309. Mackenzie N, Parry L, Tasker M, et al. Anal function following third degree tears. *Colorectal Dis.* 6(2):92-96.
310. Magro PMH, Saenz EV, Zavala MJ, Munro RDS, Ramirez JLR. Endoanal sonography in assessment of fecal incontinence following obstetric trauma. *Ultrasound in Obstetrics & Gynecology.* 22(6):616-621.
311. Mak JKL, Lee AH, Pham NM, et al. Gestational diabetes and postnatal depressive symptoms: A prospective cohort study in Western China. *Women and Birth.* 32(3):E427-E431.
312. Maldonado PA, Good MM, McIntire DD, Pathi SD, Roshanravan SM, Corton MM. Overlapping sphincteroplasty for cloacal defect following obstetrical injury: Presenting characteristics and subjective long-term outcomes. *International Urogynecology Journal and Pelvic Floor Dysfunction.* 2014;25(1):S190.
313. Maliszewska K, Bidzan M, Swiatkowska-Freund M, Preis K. Medical and psychosocial determinants of risk of postpartum depression: a cross-sectional study. *Acta Neuropsychiatrica.* 29(6):347-355.
314. Maloni JA, Park S. Postpartum symptoms after antepartum bed rest. *Jognn-Journal of Obstetric Gynecologic and Neonatal Nursing.* 34(2):163-171.
315. Malus A, Szyluk J, Galinska-Skok B, Konarzewska B. Incidence of postpartum depression and couple relationship quality. *Psychiatria Polska.* 2016;50(6):1135-1146.
316. Mannion A, Slade P. Psychotic-like experiences in pregnant and postpartum women without a history of psychosis. *Schizophrenia Research.*160(1):118-123.
317. Marin-Morales D, Toro-Molina S, Penacoba-Puente C, Losa-Iglesias M, Carmona-Monge FJ. Relationship Between Postpartum Depression and Psychological and Biological Variables in the Initial Postpartum Period. *Maternal and Child Health Journal.* 22(6):866-873.
318. Marques J, Botelho S, Pereira LC, et al. Pelvic Floor Muscle Training Program

Increases Muscular Contractility During First Pregnancy and Postpartum: Electromyographic Study. *Neurourology and Urodynamics.* 32(7):998-1003.
319. Martinez-Galiano JM, Hern, ez-Martinez A, et al. Women's Quality of Life at 6 Weeks Postpartum: Influence of the Discomfort Present in the Puerperium. *International Journal of Environmental Research and Public Health.* 16(2):9.
320. Mathe M, Valancogne G, Atallah A, et al. Early pelvic floor muscle training after obstetrical anal sphincter injuries for the reduction of anal incontinence. *Eur J Obstet Gynecol Reprod Biol.* 199:201-206.
321. Matsuura D, Okuhara S, Oguma S, et al. Self-care (behavioral intention) during pregnancy and postnatal depression in females who conceived with in vitro fertilization (IVF). *Human Reproduction.* 2018;33:i388.
322. Mautner E, Greimel E, Trutnovsky G, Daghofer F, Egger JW, Lang U. Psychosocial aspects in pregnancy and postpartum. *Journal of Psychosomatic Research.* 2010;68(6):647-648.
323. McCall-Hosenfeld JS, Phiri K, Schaefer E, Zhu JJ, Kjerulff K. Trajectories of Depressive Symptoms Throughout the Peri- and Postpartum Period: Results from the First Baby Study. *Journal of Women's Health.* 25(11):1112-1121.
324. McCoy SJB, Beal JM, Payton ME, Stewart AL, DeMers AM, Watson GH. Correlations of visual analog scales with Edinburgh Postnatal Depression Scale. *Journal of Affective Disorders.* 86(2):295-297.
325. McCoy SJB, Beal JM, Saunders B, Hill EN, Payton ME, Watson GH. Risk factors for postpartum depression: a retrospective investigation. *Journal of Reproductive Medicine.* 2008;53(3):166-170.
326. McCoy SJB, Beal JM, Shipman SBM, Payton ME, Watson GH. Risk factors for postpartum depression: a retrospective investigation at 4-weeks postnatal and a review of the literature [corrected] [published erratum appears in J Am Osteopath Assoc. 2006 Dec;106(12):687, and 2008 Apr;108(4):217]. *JAOA: Journal of the American Osteopathic Association.* 2006;106(4):193-198.
327. McDonald EA, Gartl, D, Small R, Brown SJ. Frequency, severity and persistence of postnatal dyspareunia to 18 months post partum: A cohort study. *Midwifery.* 34:15-20.
328. McKeen DM, George RB, Boyd JC, Allen VM, Pink A. Transversus abdominis plane block does not improve early or late pain outcomes after Cesarean delivery: a randomized controlled trial. *Can J Anaesth.* 61(7):631-640.
329. McManus BM, Poehlmann J, McManus BM, Poehlmann J. Parent-child interaction, maternal depressive symptoms and preterm infant cognitive function. *Infant Behavior & Development.* 2012;35(3):489-498.
330. McVeigh C. Functional status after childbirth: a comparison of Australian women from English and non-English speaking backgrounds. *Aust Coll Midwives Inc J.* 10(2):15-21.
331. McVeigh C. Functional status after childbirth in an Australian sample. *JOGNN: Journal of Obstetric, Gynecologic & Neonatal Nursing.* 1998; 27(4):402-409.
332. McVeigh C, Chaboyer W. Reliability and validity of the Inventory of Functional Status after Childbirth when used in an Australian population. *Nurs Health Sci.* 4(3):107-112.
333. McVeigh C, Smith M. A comparison of adult and teenage mother's self-esteem and satisfaction with social support. *Midwifery.* 16(4):269-276.
334. McVeigh CA. An Australian study of functional status after childbirth. *Midwifery.* 1997;13(4):172-178.
335. McVeigh CA. Investigating the relationship between satisfaction with social support

© 2020 Sultan P et al. *JAMA Network Open.*

and functional status after childbirth. *MCN: The American Journal of Maternal Child Nursing.* 2000;25(1):25-30.
336. Meager I, Milgrom J. Group treatment for postpartum depression: A pilot study. *Australian and New Zealand Journal of Psychiatry.* 30(6):852-860.
337. Meky HK, Shaaban MM, Ahmed MR, Mohammed TY. Prevalence of postpartum depression regarding mode of delivery: a cross-sectional study. *J Matern Fetal Neonatal Med.*1-8.
338. Mendes I, Azeredo Z, Rodrigues R. Validation of the portuguese version of maternal postpartum quality of life questionnaire-MAPP-QOL. *Atencion Primaria.* 2014;46:29.
339. Menos MD, Wilson A. Affective experiences and levels of self-organization in maternal postpartum depression. *Psychoanalytic Psychology.* 15(3):396-419.
340. Micali N, Simonoff E, Treasure J. Pregnancy and post-partum depression and anxiety in a longitudinal general population cohort: The effect of eating disorders and past depression. *Journal of Affective Disorders.* 131(1):150-157.
341. Milne LC, Greenway P, Hansen L. Predictors of postnatal depression in a community sample. *Neonatal, Paediatric & Child Health Nursing.* 2007;10(1):20-26.
342. Mirghafourv, M, Mohammad-Alizadeh-Char, et al. Psychometric Properties of the Iranian Version of the Inventory of Functional Status after Childbirth (IFSAC). *Iranian Red Crescent Medical Journal.* 19(5):9.
343. Misund AR, Nerdrum P, Diseth TH. Mental health in women experiencing preterm birth. *Bmc Pregnancy and Childbirth.* 14:8.
344. Moore ER, Bergman N, Anderson GC, Medley N. Early skin-to-skin contact for mothers and their healthy newborn infants. *Cochrane Database of Systematic Reviews.* 2016(11):159.
345. Mori T, Tsuchiya KJ, Matsumoto K, Suzuki K, Mori N, Takei N. Psychosocial risk factors for postpartum depression and their relation to timing of onset: the Hamamatsu Birth Cohort (HBC) Study. *J Affect Disord.* 135(1):341-346.
346. Morof D, Barrett G, Peacock J, Victor CR, Manyonda I. Postnatal depression and sexual health after childbirth. *Obstetrics and Gynecology.* 102(6):1318-1325.
347. Mortazavi F, Chaman R, Mousavi SA, Khosravi A, Ajami ME. Maternal psychological state during the transition to motherhood: A longitudinal study. *Asia-Pacific Psychiatry.* 5(2):E49-E57.
348. Murphy FL. *Effects of post-discharge nursing visits on emotional and parental adjustment of postpartum women*, Texas Woman's University; 1989.
349. Namaky D, Herzberg J, Dehoop T, Jones E, Wedig K, Marcotte M. Long-term maternal depression after expectant management of early preterm birth. *J Reprod Med.* 56(11):479-484.
350. Navarro P, García-Esteve L, Ascaso C, Aguado J, Gelabert E, Martín-Santos R. Non-psychotic psychiatric disorders after childbirth: Prevalence and comorbidity in a community sample. *Journal of Affective Disorders.* 2008;109(1):171-176.
351. Nebioglu M, Kabalcioglu F, Eroglu Y, Yalniz H. The investigating risk factors for postpartum depression in seasonal farm worker women in sanliurfa (Turkey). *Archives of Women's Mental Health.* 2013;16:S131-S132.
352. Ngai FW, Chan SW, Ip WY. The effects of a childbirth psychoeducation program on learned resourcefulness, maternal role competence and perinatal depression: a quasi-experiment. *Int J Nurs Stud.* 46(10):1298-1306.
353. Nieminen K, Berg I, Frankenstein K, et al. Internet-provided cognitive behaviour therapy of posttraumatic stress symptoms following childbirth-a randomized controlled trial. *Cognitive Behaviour Therapy.* 2016;45(4):287-306.
354. Niklasson B, Ohman SG, Segerdahl M, Blanck A. Risk factors for persistent pain and

© 2020 Sultan P et al. *JAMA Network Open.*

its influence on maternal wellbeing after cesarean section. *Acta Obstetricia Et Gynecologica Scandinavica.* 94(6):622-628.
355. Niksalehi S, Taghadosi M, Mazhariazad F, Tashk M. The effectiveness of mobile phone text massaging support for mothers with postpartum depression: A clinical before and after study. *J Family Med Prim Care.* 7(5):1058-1062.
356. Nishimura A, Fujita Y, Katsuta M, Ishihara A, Ohashi K. Paternal postnatal depression in Japan: an investigation of correlated factors including relationship with a partner. *Bmc Pregnancy and Childbirth.* 15:8.
357. Nolens B, van den Akker T, Lule J, Twinomuhangi S, van Roosmalen J, Byamugisha J. Birthing experience and quality of life after vacuum delivery and second-stage caesarean section: a prospective cohort study in Uganda. *Tropical Medicine & International Health.* 23(8):914-922.
358. Noor NM, Abd Aziz A, Mostapa MR, Awang Z. Validation of the Malay Version of the Inventory of Functional Status after Childbirth Questionnaire. *Biomed Research International.* 2015;2015:10.
359. Norderval S, Nsubuga D, Bjelke C, Frasunek J, Myklebust I, Vonen B. Anal incontinence after obstetric sphincter tears: incidence in a Norwegian county. *Acta Obstetricia Et Gynecologica Scandinavica.* 83(10):989-994.
360. Norderval S, Røssaak K, Markskog A, et al. Incontinence after primary repair of obstetric anal sphincter tears is related to relative length of reconstructed external sphincter: a case-control study. *Ultrasound in Obstetrics & Gynecology.* 2012;40(2):207-214.
361. Norhayati MN, Aniza A, Hazlina NHN, Azman MY. Psychometric properties of the revised Malay version Medical Outcome Study Social Support Survey using confirmatory factor analysis among postpartum mothers. *Asia-Pacific Psychiatry.* 7(4):398-405.
362. Norhayati MN, Hazlina NHN, Aniza A. Functional status of women with and without severe maternal morbidity: A prospective cohort study. *Women and Birth.* 29(5):443-449.
363. Norhayati MN, Nik Hazlina NH, Aniza AA. Immediate and long-term relationship between severe maternal morbidity and health-related quality of life: a prospective double cohort comparison study. *BMC Public Health.* 16(1):818.
364. Norhayati MN, Nik Hazlina NH, Aniza AA, Asrenee AR. Severe Maternal Morbidity and Postpartum Depressive Symptomatology: A Prospective Double Cohort Comparison Study. *Res Nurs Health.* 39(6):415-425.
365. O'Reilly A, Choby D, Séjourné N, Callahan S. Feelings of control, unconditional self-acceptance and maternal self-esteem in women who had delivered by caesarean. *Journal of Reproductive & Infant Psychology.* 2014;32(4):355-365.
366. Oakley SH, Ghodsi VC, Crisp CC, et al. Impact of Pelvic Floor Physical Therapy on Quality of Life and Function After Obstetric Anal Sphincter Injury: A Randomized Controlled Trial. *Female Pelvic Medicine and Reconstructive Surgery.* 22(4):205-213.
367. Oakley SH, Ghodsi VC, Crisp CC, et al. Effects of physical therapy on pelvic floor symptoms and quality of life in postpartum women following severe perineal trauma: A randomized controlled trial. *Female Pelvic Medicine and Reconstructive Surgery.* 2015;21(5):S18.
368. Ohashi Y, Kitamura T, Sakanashi K, Tanaka T. Postpartum Bonding Disorder: Factor Structure, Validity, Reliability and a Model Comparison of the Postnatal Bonding Questionnaire in Japanese Mothers of Infants. *Healthcare (Basel).* 4(3).
369. Ohoka H, Koide T, Goto S, et al. Effects of maternal depressive symptomatology during pregnancy and the postpartum period on infant-mother attachment. *Psychiatry*

© 2020 Sultan P et al. *JAMA Network Open.*

and Clinical Neurosciences. 68(8):631-639.
370. Olde E, Kleber RJ, van der Hart O, Pop VJM. Childbirth and posttraumatic stress responses - A validation study of the Dutch Impact of Event Scale - Revised. *European Journal of Psychological Assessment.* 2006;22(4):259-267.
371. Orbach-Zinger S, au R, Ben Harousch A, et al. The Relationship Between Women's Intention to Request a Labor Epidural Analgesia, Actually Delivering With Labor Epidural Analgesia, and Postpartum Depression at 6 Weeks: A Prospective Observational Study. *Anesthesia and Analgesia.* 126(5):1590-1597.
372. Ortner CM, Turk DC, Theodore BR, Siaulys MM, Bollag LA, au R. The Short-FormMcGill Pain Questionnaire-Revised to Evaluate Persistent Pain and Surgery-Related Symptoms in Healthy Women Undergoing a Planned Cesarean Delivery. *Regional Anesthesia and Pain Medicine.* 39(6):478-486.
373. Parizek A, Mikesova M, Jirak R, et al. Steroid hormones in the development of postpartum depression. *Physiol Res.* 2014;63:S277-282.
374. Park ER, Psaros C, Traeger L, et al. Development of a Postpartum Stressor Measure. *Maternal and Child Health Journal.* 19(10):2094-2101.
375. Patel RR, Murphy DJ, Peters TJ. Operative delivery and postnatal depression: a cohort study. *Bmj.*330(7496):879.
376. Paul IM, Downs DS, Schaefer EW, Beiler JS, Weisman CS. Postpartum Anxiety and Maternal-Infant Health Outcomes. *Pediatrics.* 131(4):E1218-E1224.
377. Pauls RN, Occhino JA, Dryfhout VL. Effects of pregnancy on female sexual function and body image: A prospective study. *Journal of Sexual Medicine.* 5(8):1915-1922.
378. Pereira LC, Botelho S, Marques J, et al. Electromyographic pelvic floor activity: Is there impact during the female life cycle? *Neurourol Urodyn.* 35(2):230-234.
379. Perez F, Catalan A, Morales A, et al. Assessment of postpartum depression in a group of chilean parents. *Journal of Men's Health.* 2018;14(2):E56-E64.
380. Petrou S, Kim SW, McParl, P, Boyle EM. Mode of Delivery and Long-Term Health-Related Quality-of-Life Outcomes: A Prospective Population-Based Study. *Birth-Issues in Perinatal Care.* 44(2):110-119.
381. Petrozzi A, Gagliardi L. Breastfeeding self-efficacy scale: Validation of the Italian version and correlation with breast-feeding at 3 months. *Journal of Pediatric Gastroenterology and Nutrition.* 2016;62(1):137-139.
382. Pfost KS, Stevens MJ, Lum CU. The relationship of demographic variables, antepartum depression, and stress to postpartum depression. *J Clin Psychol.* 46(5):588-592.
383. Polachek IS, Harari LH, Baum M, Strous RD. Postpartum Anxiety in a Cohort of Women from the General Population: Risk Factors and Association with Depression during Last Week of Pregnancy, Postpartum Depression and Postpartum PTSD. *Israel Journal of Psychiatry and Related Sciences.* 2014;51(2):128-134.
384. Pollock JI, Manaseki-Holl, S, Patel V. Depression in Mongolian women over the first 2 months after childbirth: Prevalence and risk factors. *Journal of Affective Disorders.*116(1):126-133.
385. Pop VJ, Truijens SE, Spek V, Wijnen HA, van Son MJ, Bergink V. A new concept of maternity blues: Is there a subgroup of women with rapid cycling mood symptoms? *J Affect Disord.*177:74-79.
386. Pourkhiz Z, Mohammad-Alizadeh-Char, abi S, et al. Effect of Pelvic Floor Muscle Training on Female Sexual Function During Pregnancy and Postpartum: A Randomized Controlled Trial. *Iranian Red Crescent Medical Journal.* 19(10):8.
387. Powell C, Bamber D, Long J, et al. Mental health and well-being in parents of excessively crying infants: Prospective evaluation of a support package. *Child Care*

© 2020 Sultan P et al. *JAMA Network Open.*

Health Dev. 44(4):607-615.
388. Priest SR, Henderson J, Evans SF, Hagan R. Stress debriefing after childbirth: A randomised controlled trial. *Medical Journal of Australia.* 2003;178(11):542-545.
389. Quiboeuf E, Saurel-Cubizolles MJ, Fritel X, Grp EM-CCS. Trends in urinary incontinence in women between 4 and 24 months postpartum in the EDEN cohort. *Bjog-an International Journal of Obstetrics and Gynaecology.* 123(7):1222-1228.
390. Rabiei L, Mazaheri MA, Masoudi R, Hasheminia SAM. Fordyce happiness program and postpartum depression. *Journal of Research in Medical Sciences.* 19(3):251-256.
391. Reading AE. A comparison of the McGill Pain Questionnaire in chronic and acute pain. *Pain.*13(2):185-192.
392. Richter HE, Nager CW, Burgio KL, et al. Incidence and Predictors of Anal Incontinence After Obstetric Anal Sphincter Injury in Primiparous Women. *Female Pelvic Medicine and Reconstructive Surgery.* 21(4):182-189.
393. Rogers RG, Borders N, Leeman LM, Albers LL. Does Spontaneous Genital Tract Trauma Impact Postpartum Sexual Function? *Journal of Midwifery & Women's Health.* 54(2):98-103.
394. Rogers RG, Leeman LM, Borders N, et al. Contribution of the second stage of labour to pelvic floor dysfunction: a prospective cohort comparison of nulliparous women. *Bjog-an International Journal of Obstetrics and Gynaecology.* 121(9):1145-1153.
395. Rogers RG, Leeman LM, Migliaccio L, Albers LL. Does the severity of spontaneous genital tract trauma affect postpartum pelvic floor function? *Int Urogynecol J Pelvic Floor Dysfunct.* 19(3):429-435.
396. Ros C, Martinez-Franco EM, Elias N, Lopez M, Palacio M, Espuña M. Pelvic floor symptoms and strength of pelvic floor muscles in women with history of obstetric anal sphincter injuries. Analysis according to the mode of delivery. *Neurourology and Urodynamics.* 2014;33(6):890-892.
397. Ros C, Martinez-Franco EM, Elias N, et al. Persistency of anal sphincter defects in women with obstetric anal sphincter injuries and the function of pelvic floor muscles after delivery. How they influence on anal incontinence symptoms? *Neurourology and Urodynamics.* 2015;34:S176-S177.
398. Ross LE, Gilbert Evans SE, Sellers EM, Romach MK. Measurement issues in postpartum depression part 2: assessment of somatic symptoms using the Hamilton Rating Scale for Depression. *Arch Women's Ment Health.*6(1):59-64.
399. Rouhe H, Salmela-Aro K, Toivanen R, et al. Group psychoeducation with relaxation for severe fear of childbirth improves maternal adjustment and childbirth experience - a randomised controlled trial. *Journal of Psychosomatic Obstetrics and Gynecology.*36(1):1-9.
400. Rowe-Murray HJ, Fisher JR. Operative intervention in delivery is associated with compromised early mother-infant interaction. *Bjog.*108(10):1068-1075.
401. Ruchala PL. *The postpartum experience: a study of maternal concerns, confidence, and support*, Rush University, College of Nursing; 1991.
402. Sadat Z, Abedzadeh Kalahroudi M, Kafaei Atrian M, Karimian Z, Sooki Z. The impact of postpartum depression on quality of life in women after child's birth. *Iranian Red Crescent Medical Journal.* 16(2):e14995.
403. Sadat Z, Atrian MK, Alavi NM, Abbaszadeh F, Karimian Z, Taherian A. Effect of mode of delivery on postpartum depression in Iranian women. *Journal of Obstetrics and Gynaecology Research.* 40(1):172-177.
404. Salonen AH, Kaunonen M, Åstedt-Kurki P, Järvenpää A-L, Isoaho H, Tarkka M-T. Effectiveness of an internet-based intervention enhancing Finnish parents' parenting satisfaction and parenting self-efficacy during the postpartum period. *Midwifery.*

© 2020 Sultan P et al. *JAMA Network Open.*

2011;27(6):832-841.
405. Salonen AH, Pridham KF, Brown RL, Kaunonen M. Impact of an internet-based intervention on Finnish mothers' perceptions of parenting satisfaction, infant centrality and depressive symptoms during the postpartum year. *Midwifery.* 30(1):112-122.
406. Santoro E, Stagni-Brenca E, Olivari MG, Confalonieri E, Di Blasio P. Childbirth Narratives of Women With Posttraumatic Stress Symptoms in the Postpartum Period. *Jognn-Journal of Obstetric Gynecologic and Neonatal Nursing.* 47(3):333-341.
407. Saotome TT, Yonezawa K, Suganuma N. Sexual Dysfunction and Satisfaction in Japanese Couples During Pregnancy and Postpartum. *Sexual Medicine.* 6(4):348-355.
408. Sarberg M, Bladh M, Svanborg E, Josefsson A. Postpartum depressive symptoms and its association to daytime sleepiness and restless legs during pregnancy. *BMC Pregnancy and Childbirth.* 16(1):8.
409. Sato Y, Kato T, Kakee N. A six-month follow-up study of maternal anxiety and depressive symptoms among Japanese. *Journal of Epidemiology.*18(2):84-87.
410. Satoh A, Kitamiya C, Kudoh H, Watanabe M, Menzawa K, Sasaki H. Factors associated with late post-partum depression in Japan. *Japan Journal of Nursing Science.* 2009;6(1):27-36.
411. Savarimuthu RJ, Ezhilarasu P, Charles H, Antonisamy B, Kurian S, Jacob KS. Post-partum depression in the community: a qualitative study from rural South India. *Int J Soc Psychiatry.* 56(1):94-102.
412. Saydam BK, Akyuz MD, Sogukpinar N, Turfan EC. Effect of delivery method on sexual dysfunction. *Journal of Maternal-Fetal & Neonatal Medicine.*32(4):568-572.
413. Sayed Ahmed WA, Kishk EA, Farhan RI, Khamees RE. Female sexual function following different degrees of perineal tears. *Int Urogynecol J.* 28(6):917-921.
414. Scheer I, Andrews V, Thakar R, Sultan AH. Urinary incontinence after obstetric anal sphincter injuries (OASIS)-is there a relationship? *International Urogynecology Journal.* 19(2):179-183.
415. Schwerla F, Rother K, Rother D, Ruetz M, Resch KL. Osteopathic Manipulative Therapy in Women With Postpartum Low Back Pain and Disability: A Pragmatic Randomized Controlled Trial. *J Am Osteopath Assoc.* 115(7):416-425.
416. Sealy-Jefferson S, Giurgescu C, Slaughter-Acey J, Caldwell C, Misra D. Neighborhood Context and Preterm Delivery among African American Women: the Mediating Role of Psychosocial Factors. *Journal of Urban Health.* 2016;93(6):984-996.
417. Séjourné N, De la Hammaide M, Moncassin A, O'Reilly A, Chabrol H. Study of the relations between the pain of childbirth and postpartum, and depressive and traumatic symptoms. *Gynecologie Obstetrique Fertilite et Senologie.* 2018;46(9):658-663.
418. Sekiguchi Y, Utsugisawa Y, Azekosi Y, et al. Laxity of the Vaginal Introitus After Childbirth: Nonsurgical Outpatient Procedure for Vaginal Tissue Restoration and Improved Sexual Satisfaction Using Low-Energy Radiofrequency Thermal Therapy. *Journal of Women's Health (15409996).* 2013;22(9):775-781.
419. Senturk V, Hanlon C, Medhin G, et al. Impact of perinatal somatic and common mental disorder symptoms on functioning in Ethiopian women: The P-MaMiE population-based cohort study. *Journal of Affective Disorders.* 136(3):340-349.
420. Seppanen P, Sund R, Ala-Kokko T, et al. Obstetric patients' health-related quality of life before and after intensive care. *Australian Critical Care.* 32(2):116-121.
421. Shaban Z, Dolatian M, Shams J, Alavi-Majd H, Mahmoodi Z, Sajjadi H. Post-Traumatic Stress Disorder (PTSD) Following Childbirth: Prevalence and Contributing Factors. *Iranian Red Crescent Medical Journal.* 15(3):177-182.

© 2020 Sultan P et al. *JAMA Network Open.*

422. Sharma C, Verma A, Soni A, Thusoo M, Mahajan VK, Verma S. A randomized controlled trial comparing cosmetic outcome after skin closure with 'staples' or 'subcuticular sutures' in emergency cesarean section. *Arch Gynecol Obstet.* 290(4):655-659.
423. Shaw E, Levitt C, Wong S, Kaczorowski J, McMaster Univ Postpartum Res G. Systematic review of the literature on postpartum care: Effectiveness of postpartum support to improve maternal parenting, mental health, quality of life, and physical health. *Birth-Issues in Perinatal Care.* 33(3):210-220.
424. Shimizu A, Nishiumi H, Okumura Y, Watanabe K. Depressive symptoms and changes in physiological and social factors 1 week to 4 months postpartum in Japan. *Journal of Affective Disorders.* 179:175-182.
425. Shorey S, Chan SWC, Chong YS, He HG. A randomized controlled trial of the effectiveness of a postnatal psychoeducation programme on self-efficacy, social support and postnatal depression among primiparas. *Journal of Advanced Nursing.* 71(6):1260-1273.
426. Simavli S, Kaygusuz I, Gumus I, Usluogullari B, Yildirim M, Kafali H. Effect of music therapy during vaginal delivery on postpartum pain relief and mental health. *Journal of Affective Disorders.* 156:194-199.
427. Singata-Madliki M, Hofmeyr GJ, Lawrie TA. The effect of depot medroxyprogesterone acetate on postnatal depression: a randomised controlled trial. *Journal of Family Planning and Reproductive Health Care.* 42(3):171-176.
428. Sioutis D, Thakar R, Sultan AH. Overdiagnosis and rising rate of obstetric anal sphincter injuries (OASIS): time for reappraisal. *Ultrasound in Obstetrics & Gynecology.* 50(5):642-647.
429. Sit D. Seasonality of depression risk and suicidal symptoms in the postpartum period. *Neuropsychopharmacology.* 2010;35:S378.
430. Sit D, Seltman H, Wisner KL. Seasonal effects on depression risk and suicidal symptoms in postpartum women. *Depression and Anxiety.* 28(5):400-405.
431. Siu BWM, Ip P, Chow HMT, et al. Impairment of Mother-Infant Relationship Validation of the Chinese Version of Postpartum Bonding Questionnaire. *Journal of Nervous and Mental Disease.* 198(3):174-179.
432. Sivertsen B, Hysing M, Dørheim SK, Eberhard-Gran M. Trajectories of maternal sleep problems before and after childbirth: a longitudinal population-based study. *BMC Pregnancy & Childbirth.* 2015;15(1):129-129.
433. Sivertsen B, Petrie KJ, Skogen JC, Hysing M, Eberhard-Gran M. Insomnia before and after childbirth: The risk of developing postpartum pain-A longitudinal population-based study. *European Journal of Obstetrics & Gynecology and Reproductive Biology.* 210:348-354.
434. Skari H, Skreden M, Malt UF, et al. Comparative levels of psychological distress, stress symptoms depression and anxiety after childbirth - a prospective population-based study of mothers and fathers. *BJOG-an International Journal of Obstetrics and Gynaecology.* 109(10):1154-1163.
435. Skreden M, Skari H, Bjork MD, et al. Psychological distress in mothers and fathers of preschool children: a 5-year follow-up study after birth. *BJOG-an International Journal of Obstetrics and Gynaecology.* 115(4):462-471.
436. Small R, Johnston V, Orr A. Depression after childbirth: The views of medical students and women compared. *Birth-Issues in Perinatal Care.*24(2):109-115.
437. Small R, Lumley J, Donohue L, Potter A, Waldenstrom U. Randomised controlled trial of midwife led debriefing to reduce maternal depression after operative childbirth. *BMJ.* 321(7268):1043-1047.

© 2020 Sultan P et al. *JAMA Network Open.*

438. Small R, Lumley J, Toomey L. Midwife-led debriefing after operative birth: four to six year follow-up of a randomised trial ISRCTN24648614. *BMC Medicine*.4:4.
439. Soares ADS, Couceiro TCD, Lima LC, Flores FLL, Alcoforado EMB, Couceiro RD. Association of Pain Catastrophizing with the Incidence and Severity of Acute and Persistent Perineal Pain after Natural Childbirth: Longitudinal Cohort Study. *Revista Brasileira De Anestesiologia.* 63(4):317-321.
440. Soderquist J, Wijma B, Thorbert G, Wijma K. Risk factors in pregnancy for post-traumatic stress and depression after childbirth. *BJOG.* 116(5):672-680.
441. Soligo M, Turri A, Scebba I, et al. 2/3D ultrasound abnormalities and pelvic floor symptoms persistence one year after delivery: Is there a correlation? *Neurourology and Urodynamics.* 2016;35:S148-S149.
442. Soltani N, Abedian Z, Mokhber N, Esmaily H. The Association of Family Support After Childbirth With Posttraumatic Stress Disorder in Women With Preeclampsia. *Iranian Red Crescent Medical Journal.* 17(10):6.
443. Song M, Ishii H, Toda M, et al. Association Between Sexual Health and Delivery Mode. *Sexual Medicine.* 2(4):153-158.
444. Spiteri MC, Jomeen J, Martin CR. Reimagining the General Health Questionnaire as a measure of emotional wellbeing: A study of postpartum women in Malta. *Women and Birth.* 26(4):E105-E111.
445. Sredniawa A, Kruk K, Jarczewska DL, et al. Postpartum depression and breastfeeding in primary care in Krakow, Poland. *Clinical and Experimental Obstetrics & Gynecology.* 2018;45(6):880-885.
446. Stadlmayr W, Bitzer J, Amsler F, et al. Acute stress reactions in the first 3 weeks postpartum: a study of 219 parturients. *Eur J Obstet Gynecol Reprod Biol.* 135(1):65-72.
447. Stevenson MD, Scope A, Sutcliffe PA, et al. Group cognitive behavioural therapy for postnatal depression: a systematic review of clinical effectiveness, cost-effectiveness and value of information analyses. *Health Technology Assessment.* 14(44):IX-+.
448. Strickl, TL, James R, et al. Psychological characteristics related to cocaine use during pregnancy: a postpartum assessment. *J Natl Med Assoc.* 85(10):758-760.
449. Sword W, Bai YQ, Thabane L, et al. Predictors of postpartum depression over the first year following childbirth. *Journal of Paediatrics and Child Health.* 2012;48:62.
450. Sylven SM, Elenis E, Michelakos T, et al. Thyroid function tests at delivery and risk for postpartum depressive symptoms. *Psychoneuroendocrinology.* 38(7):1007-1013.
451. Tachibana Y, Koizumi N, Akanuma C, et al. Integrated mental health care in a multidisciplinary maternal and child health service in the community: the findings from the Suzaka trial. *BMC Pregnancy & Childbirth.* 2019;19(1).
452. Tan EC, Chua TE, Lee TMY, Tan HS, Ting JLY, Chen HY. Case-control study of glucocorticoid receptor and corticotrophin-releasing hormone receptor gene variants and risk of perinatal depression. *Bmc Pregnancy and Childbirth.*15:6.
453. Tan EC, Lim HW, Chua TE, Tan HS, Lee TMY, Chen HY. Investigation of variants in estrogen receptor genes and perinatal depression. *Neuropsychiatric Disease and Treatment.* 2018;14:919-925.
454. Tani F, Castagna V. Maternal social support, quality of birth experience, and post-partum depression in primiparous women. *Journal of Maternal-Fetal & Neonatal Medicine.* 2017;30(6):689-692.
455. Tatano Beck C, Gable RK, Sakala C, Declercq ER. Postpartum Depressive Symptomatology: Results from a Two-Stage US National Survey. *Journal of Midwifery & Women's Health.* 2011;56(5):427-435.
456. Taylor DA, Merten SL, ercoe GD, et al. Abdominoplasty Improves Low Back Pain

© 2020 Sultan P et al. *JAMA Network Open.*

and Urinary Incontinence. *Plastic and Reconstructive Surgery.* 141(3):637-645.
457. Taylor J, Johnson M. How women manage fatigue after childbirth. *Midwifery.* 2010;26(3):367-375.
458. Teissedre F, Chabrol H. Detecting women at risk for postnatal depression using the Edinburgh Postnatal Depression Scale at 2 to 3 days postpartum. *Canadian Journal of Psychiatry-Revue Canadienne De Psychiatrie.* 49(1):51-54.
459. Tennfjord MK, Hilde G, Staer-Jensen J, Siafarikas F, Engh ME, Bo K. Effect of postpartum pelvic floor muscle training on vaginal symptoms and sexual dysfunctionsecondary analysis of a randomised trial. *BJOG-an International Journal of Obstetrics and Gynaecology.*123(4):634-642.
460. Tennfjord MK, Hilde G, Staer-Jensen J, Siafarikas F, Engh ME, Bo K. Coital Incontinence and Vaginal Symptoms and the Relationship to Pelvic Floor Muscle Function in Primiparous Women at 12 Months Postpartum: A Cross-Sectional Study. *Journal of Sexual Medicine.* 12(4):994-1003.
461. Tham V, Christensson K, Ryding EL. Sense of coherence and symptoms of post-traumatic stress after emergency caesarean section. *Acta Obstetricia Et Gynecologica Scandinavica.* 2007;86(9):1090-1096.
462. Thome M, Orlygsdottir B, Elvarsson BT. Evaluation of the clinical effect of an on-line course for community nurses on post-partum emotional distress: a community-based longitudinal time-series quasi-experiment. *Scandinavian Journal of Caring Sciences.* 2012;26(3):494-504.
463. Thompson JF, Roberts CL, Currie MJ, Ellwood DA. Early discharge and postnatal depression: a prospective cohort study. *Medical Journal of Australia.*172(11):532-536.
464. Thompson WM, Harris B, Lazarus J, Richards C. A comparison of the performance of rating scales used in the diagnosis of postnatal depression. *Acta Psychiatrica Scandinavica.*98(3):224-227.
465. Tin RY, Schulz J, Gunn B, Flood C, Rosychuk RJ. The prevalence of anal incontinence in post-partum women following obstetrical anal sphincter injury. *Int Urogynecol J.*21(8):927-932.
466. Tobback E, Behaeghel K, Hanoulle I, et al. Comparison of subjective sleep and fatigue in breast- and bottle-feeding mothers. *Midwifery.*47:22-27.
467. Torkan B, Parsay S, Lamyian M, Kazemnejad A, Montazeri A. Postnatal quality of life in women after normal vaginal delivery and caesarean section. *BMC Pregnancy and Childbirth.*9:7.
468. Torstensson T, Lindgren A, Kristiansson P. Improved function in women with persistent pregnancy-related pelvic pain after a single corticosteroid injection to the ischiadic spine: a randomized double-blind controlled trial. *Physiother Theory Pract.*29(5):371-378.
469. Trivino-Juarez JM, Romero-Ayuso D, Nieto-Pereda B, et al. Health related quality of life of women at the sixth week and sixth month postpartum by mode of birth. *Women and Birth.*30(1):29-39.
470. Tsai S-Y, Thomas KA. Sleep disturbances and depressive symptoms in healthy postpartum women: A pilot study. *Research in Nursing & Health.* 2012;35(3):314-323.
471. Tsuchiya M, Mori E, Sakajo A, Iwata H, Maehara K, Tamakoshi K. Cross-sectional and longitudinal validation of a 13-item fatigue scale among Japanese postpartum mothers. *International Journal of Nursing Practice.* 22:5-13.
472. Tuohy A, McVey C. Experience of pregnancy and delivery as predictors of postpartum depression. *Psychology, Health & Medicine.* 2008;13(1):43-47.

© 2020 Sultan P et al. *JAMA Network Open.*

473. Turel FD, Langer S, Shek KL, Dietz HP. Medium- to Long-term Follow-up of Obstetric Anal Sphincter Injury. *Diseases of the Colon & Rectum.* 62(3):348-356.
474. Turkstra E, Gamble J, Creedy DK, et al. PRIME: impact of previous mental health problems on health-related quality of life in women with childbirth trauma. *Archives of Women's Mental Health.* 16(6):561-564.
475. Turkstra E, Mihala G, Scuffham PA, et al. An economic evaluation alongside a randomised controlled trial on psycho-education counselling intervention offered by midwives to address women's fear of childbirth in Australia. *Sexual & Reproductive Healthcare.* 11:1-6.
476. Ugarriza DN. Screening for postpartum depression. *J Psychosoc Nurs Ment Health Serv.* 38(12):44-51.
477. Valente MA, Kh, uja KS. Layered surgical repair of traumatic cloacal deformities: technical details and functional outcomes. *Tech Coloproctol.* 16(2):153-156.
478. van Anders SM, Hipp LE, Low LK. Exploring Co-Parent Experiences of Sexuality in the First 3 Months after Birth. *Journal of Sexual Medicine.* 10(8):1988-1999.
479. van de Waarsenburg MK, Withagen MI, Grob AT, Schweitzer KJ, van Veelen GA, van der Vaart CH. Mean echogenicity and area of puborectalis muscle in women with stress urinary incontinence during pregnancy and after delivery. *Int Urogynecol J.* 27(11):1723-1728.
480. Van Der Waerden J, Galéra C, Larroque B, Saurel-Cubizolles MJ, Sutter-Dallay AL, Melchior M. Timing, chronicity and severity of maternal depressive symptoms and children's behavior at age five: The EDEN mother-child cohort. *Archives of Women's Mental Health.* 2015;18(2):277.
481. Vasseur A, Lepigeon K, Baud D, et al. Counseling after perineal laceration: does it improve functional outcome? *International Urogynecology Journal.* 30(6):925-931.
482. Vennat D, Belot RA, Capponi I, Mellier D. Lack of the family support in the post-partum: What impacts on the emergence of maternal distress? *Neuropsychiatrie de l'Enfance et de l'Adolescence.* 2018;66(6):370-381.
483. Verreault N, Da Costa D, March, et al. PTSD following childbirth: A prospective study of incidence and risk factors in Canadian women. *Journal of Psychosomatic Research.* 2012;73(4):257-263.
484. Vogeli JM, Hooker SA, Everhart KD, Kaplan PS. Psychometric properties of the postpartum depression screening scale beyond the postpartum period. *Res Nurs Health.* 41(2):185-194.
485. Volloyhaug I, Van Gruting IM, Sultan AH, Thakar R. Does mode of delivery affect bladder neck, urethral mobility and urinary incontinence symptoms four years after delivery? *International Urogynecology Journal and Pelvic Floor Dysfunction.* 2016;27(1):S36-S37.
486. von Sydow K, Ullmeyer M, Happ N. Sexual activity during pregnancy and after childbirth: results from the Sexual Preferences Questionnaire. *J Psychosom Obstet Gynaecol.* 22(1):29-40.
487. Waddell V, Schaffir J. Postpartum sexual function and breastfeeding. *Journal of Sexual Medicine.* 2010;7:147.
488. Wan EL, Goldstein AT, Tolson H, Dellon AL. Injury to Perineal Branch of Pudendal Nerve in Women: Outcome from Resection of the Perineal Branches. *J Reconstr Microsurg.* 33(6):395-401.
489. Wan EY, Moyer CA, Harlow SD, Fan ZT, Jie Y, Yang HX. Postpartum depression and traditional postpartum care in China: Role of Zuoyuezi. *International Journal of Gynecology & Obstetrics.* 104(3):209-213.
490. Watkins S, Meltzer-Brody S, Zolnoun D, Stuebe A. Early Breastfeeding Experiences

© 2020 Sultan P et al. *JAMA Network Open.*

and Postpartum Depression. *Obstetrics and Gynecology.*118(2):214-221.
491. Watt S, Sword W, Krueger P, Sheehan D. A cross-sectional study of early identification of postpartum depression: Implications for primary care providers from The Ontario Mother & Infant Survey. *BMC Family Practice.*3:1-7.
492. Webster J, Nicholas C, Velacott C, Cridl, N, Fawcett L. Validation of the WHOQOL-BREF among women following childbirth. *Australian & New Zealand Journal of Obstetrics & Gynaecology.* 50(2):132-137.
493. Westad S, Backe B, Salvesen KÅ, et al. A 12-week randomised study comparing intravenous iron sucrose versus oral ferrous sulphate for treatment of postpartum anemia. *Acta Obstetricia et Gynecologica Scandinavica.* 2008;87(9):916-923.
494. Wickberg B, Hwang CP. Counselling of postnatal depression: A controlled study on a population based Swedish sample. *Journal of Affective Disorders.* 39(3):209-216.
495. Wiklund I, Edman G, Andolf E. Cesarean section on maternal request: reasons for the request, self-estimated health, expectations, experience of birth and signs of depression among first-time mothers. *Acta Obstetricia Et Gynecologica Scandinavica.* 2007;86(4):451-456.
496. Wilkie S, Crawley R, Button S, Thornton A, Ayers S. Assessing physical symptoms during the postpartum period: reliability and validity of the primary health questionnaire somatic symptom subscale (PHQ-15). *Journal of Psychosomatic Obstetrics & Gynecology.* 2018;39(1):56-63.
497. Wilkins C, Baker R, Bick D, Thomas P. Emotional processing in childbirth: a predictor of postnatal depression? *British Journal of Midwifery.* 2009;17(3):154-159.
498. Wisner KL, Sit DKY. Screening for PPD in an obstetrical hospital population. *Archives of Women's Mental Health.* 2013;16:S8.
499. Wojcik J, Dudek D, Schlegel-Zawadzka M, et al. Antepartum/postpartum depressive symptoms and serum zinc and magnesium levels. *Pharmacol Rep.* 58(4):571-576.
500. Woolhouse H, Gartl, D, Hegarty K, Donath S, Brown SJ. Depressive symptoms and intimate partner violence in the 12 months after childbirth: A prospective pregnancy cohort study. *BJOG: An International Journal of Obstetrics and Gynaecology.* 2012;119(3):315-323.
501. Woolhouse H, Gartl, D, Perlen S, Donath S, Brown SJ. Physical health after childbirth and maternal depression in the first 12 months post partum: Results of an Australian nulliparous pregnancy cohort study. *Midwifery.* 30(3):378-384.
502. Yamamoto N, Abe Y, Arima K, et al. Mental health problems and influencing factors in Japanese women 4 months after delivery. *Journal of Physiological Anthropology.* 33:6.
503. Yamashita H, Yoshida K. Impact of perinatal stress on mother-infant interaction: Mother-infant interaction and infant outcome in mothers with mood and/or anxiety disorder. *Archives of Women's Mental Health.* 2013;16:S145.
504. Yee LM, Kaimal AJ, Nakagawa S, Houston K, Kuppermann M. Predictors of Postpartum Sexual Activity and Function in a Diverse Population of Women. *Journal of Midwifery & Women's Health.* 58(6):654-661.
505. Yohay D, Weintraub AY, Mauer-Perry N, et al. Prevalence and trends of pelvic floor disorders in late pregnancy and after delivery in a cohort of Israeli women using the PFDI-20. *European Journal of Obstetrics & Gynecology and Reproductive Biology.* 200:35-39.
506. Yoshida K, Yamashita H. Clinical survey of themother-infant mental health clinic in kyushu university hospital: Psychiatric characteristics of consecutive 109 women and their infants. *Archives of Women's Mental Health.* 2013;16:S104-S105.
507. Yu CY, Hung CH, Huang MC, Chan TF. Predictors of Hyperglycemic Women's

© 2020 Sultan P et al. *JAMA Network Open.*

Perinatal Health Status. *Worldviews on Evidence-Based Nursing.* 13(6):445-453.
508. Yuvarani G, Daggumati HB, Ramach, ran S, Sudhakar S. The effect of tRa training and pilates in women with stress urinary incontinence. *Biomedicine (India).* 2018;38(3):404-407.
509. Zanardo V, Gabrieli C, Straface G, Savio F, Soldera G. The interaction of personality profile and lactation differs between mothers of late preterm and term neonates. *J Matern Fetal Neonatal Med.* 30(8):927-932.
510. Zanardo V, Volpe F, de Luca F, et al. Maternity blues: a risk factor for anhedonia, anxiety, and depression components of Edinburgh Postnatal Depression Scale. *J Matern Fetal Neonatal Med.* 1-7.
511. Zhang Y, Johnston L, Ma DM, Wang F, Zheng XL, Xu XF. An exploratory study of the effect of labor pain management on postpartum depression among Chinese women. *Ginekologia Polska.* 2018;89(11):627-636.
512. Zhou SZ, Wang XL, Wang Y. Design of a questionnaire for evaluating the quality of life of postpartum women (PQOL) in China. *Qual Life Res.* 18(4):497-508.
513. Zielinski R, Kane Low L, Smith AR, Miller JM. Body after baby: a pilot survey of genital body image and sexual esteem following vaginal birth. *Int J Women's Health.* 2017;9:189-198.
514. Zourladani A, Tsaloglidou A, Tzetzis G, Tsorbatzoudis C, Matziari C. The effect of a low impact exercise training programme on the well-being of Greek postpartum women: A randomised controlled trial. *International Sportmed Journal.* 2011;12(1):30-38.
515. Zutshi M, Ferreira P, Gurl, B, Hull T. Biologics in anal sphincter repair: To use or not to use. *Colorectal Disease.*12:4.

© 2020 Sultan P et al. *JAMA Network Open.*

eTable 1. Most Frequently Utilized Patient-Reported Outcome Measures (PROMs) Among All Included (Outpatient and Inpatient) Studies

**PROM**	**No. Studies**	**Domain (Sub-domain)**
EPDS	267	Psychosocial distress (depression)
SF-36	40	Global
FSFI	35	Sexual function
STAI	31	Psychosocial distress (anxiety)
Beck depression inventory (I/II)	29	Psychosocial distress (depression)
IES(-R)	24	Psychosocial distress (psychological)
General health questionnaire	18	Psychosocial distress (anxiety & depression)
McGill pain	17	Pain
IFSAC	16	Global
ICIQ-UI-SF	16	Pelvic floor complications (urinary)
EuroQoL 5 & 6	15	Global
UDI-6	14	Pelvic floor complications (urinary)
CES-D	14	Psychosocial distress (depression)
Profile of mood states	13	Psychosocial distress (psychological)
IIQ-7	13	Pelvic floor complications (urinary)
Patient health questionnaire	11	Psychosocial distress (depression)
Blues questionnaire	10	Psychosocial distress (depression)
Wexner scale	8	Pelvic floor complications (colorectal)
PISQ-12	8	Sexual function
SF-12	8	Global
Breastfeeding self-efficacy	8	Breastfeeding / breast health
Perinatal PTSD questionnaire	8	Psychosocial distress (psychological)
St. Mark's score	7	Pelvic floor complications (colorectal)
Perceived stress scale	7	Psychosocial distress (psychological)
Fecal incontinence severity index	7	Pelvic floor complications (colorectal)
Hospital anxiety and depression scale	7	Psychosocial distress (anxiety & depression)

EPDS=Edinburgh postnatal depression score; SF-36=Short Form (36) Health Survey; FSFI=Female Sexual Function Index; STAI=State-Trait Anxiety Inventory; IES(-R)=Impact of Event Scale (-Revised); IFSAC=Inventory of Functional Status After Childbirth; ICIQ-SF=International Consultation on Incontinence Questionnaire – urinary incontinence -short form; UDI-6=Urinary Distress Inventory, short form; CES-D=Center for Epidemiological Studies Depression scale; IIQ-7=Incontinence Impact Questionnaire, short form; PISQ-12= Pelvic organ prolapse/urinary Incontinence Sexual Questionnaire; SF-12=12-item Short Form Health Survey; PTSD=Post Traumatic Stress Disorder

© 2020 Sultan P et al. *JAMA Network Open.*

eTable 2. Classification of 201 Patient-Reported Outcome Measures (PROMs) Used to Evaluate Outpatient Recovery Following Childbirth According to Domains

Physical function	Surgical complications			Pain	Psychosocial distress			Psychosocial support	Sleep	Motherhood experience		Breast feeding / breast health	Fatigue	Sexual function	Scar /and wound healing	Global recovery
	Urinary	Gynecology	Colorectal		Psychological (Other)	Anxiety	Depression			Adapting to maternal role	Maternal – neonatal Bonding					
(2 & 2)	(17 & 72)	(7 & 16)	(11 & 47)	(8 &19)	(50 & 115)	(11 & 53)	(25 & 362)	(27 & 40)	(7 &13)	(11 & 21)	(5 & 13)	(2 & 7)	(5 &10)	(13 & 56)	(1 & 1)	(17 & 88) [Domains]
Disability rating index (1)	ICIQ-UI SF (Taiwan, Italian, Portuguese) (16)	ICIQ-VS (3)	Wexner scale (fecal 8)	McGill pain score (Taiwan) (9)	Impact of event scale (and revised form, Dutch) (21)	STAI (19)	EPDS (Chinese, Portuguese, Swedish, Japanese, Tamil, Iran, Malay) (231)	Personal resource questionnaire (6)	Pittsburgh sleep quality index (5)	Parenting sense of competence scale-efficacy sub-scale (Chinese) (6)	Mother-infant bonding scale (Portugese) (5)	Breastfeeding self efficacy scale (& short form & Italian) (6)	Modified fatigue symptom checklist (3)	FSFI (Taiwan, Thai, Japanese, Turkish, Arabic) (34)	Patient scar assessment scale (1)	SF-36 (V2, Croatia, Iran) (32)
KATZ-ADL (1)	UDI-6 (14)	Vaginal laxity questionnaire (1)	Fecal incontinence severity index (7)	Roland-Morris low back pain and disability questionnaire (2)	Profile of mood states (Japanese) (12)	Generalized anxiety disorder scale (3)	Beck depression inventory I / II (Persian) (24)	Multidimensional scale of perceived social support (4)	Bergen insomnia scale (2)	Parenting stress index (short form) (3)	Postpartum bonding questionnaire (Chinese) (5)	Breast milk expression experience (1)	Checklist individual strength (2)	PISQ-12 (8)		IFSAC (Iran) (14) [5]
	IIQ7 (13)		St. Mark's score (7)	Oswestry disability index (2)	Perinatal posttraumatic stress disorder questionnaire (PPQ) (7)	General health questionnaire + GHQ-12 (Maltese & Japanese) (16)		Medical outcome study social support survey (Malay) (3)	Turkish postpartum sleep quality scale (2)	Perceived maternal parental self-efficacy scale (2)	Attachment – caregiving balance scale (1)		Multidimensional fatigue inventory (2)	ICIQ-FLUTSsex (3)		EuroQoL 5 / 6 (13)
	Australian pelvic floor dysfunction (5)		Fecal incontinence quality of life scale (6)	Pain catastrophizing scale (2)	Perceived stress scale (6)	Hospital anxiety and depression scale (Japanese) (7)		Intimate relationship scale (2)	Insomnia severity index (1)	What being the parent of a new baby is like (revised) (2)	Mother infant togetherness survey (1)		Fatigue assessment scale (Spanish) (2)	Arizona sexual experience scale (2)		SF-12 (8)
	Pelvic floor impact questionnaire (5)			Bodily pain scale (1)	Big five inventory (5)	Hopkins symptom checklist -25 / 10 (4)		Perceived social support scale (2)	Epworth sleepiness scale (1)	Infant care survey (2)	Maternal attachment scale (1)		Fatigue continuum form (1)	Sexual function questionnaire (1)		Barkin index of maternal functioning (4) [5]
	Pelvic floor distress inventory-20 (Hebrew) (4)			Vulvar pain functional questionnaire (1)	Hung postpartum stress scale (Taiwan (4)	Aga Khan University anxiety and depression scale (2)		Berlin social support scale (2)	Sleep symptom checklist (1)	Postpartum self-evaluation questionnaire* (1)				Carol postpartum sexual function and dyspareunia assessment scale (1)		WHOQOL-BREF (3)
	ICIQ-OAB (Portuguese Taiwan) (3)		Modified Manchester health questionnaire (3)	Brief Pain inventory (1)	Rosenberg self esteem scale (4)	Depression and anxiety stress scale (2)		Duke UNC functional social support questionnaire (1)	General sleep disturbance scale (1)	Swedish parental stress questionnaire (1)				Sexual preferences questionnaire (1)		Nottingham health profile (2)
	Bristol female lower urinary tract symptoms		Cleveland clinic incontinen	OWHSQ (1)	Crown-Crisp experiential index (2)		CES-D (Taiwan) (12)	Family support scale (1)		Infancy parenting styles questionnaire				Sexual health outcomes in women		Maternal concerns questionna

© 2020 Sultan P et al. *JAMA Network Open.*

	(Chinese) (2)		ce score (3)							(1)				questionnaire (1)		ire (2) [10]
	ICIQ-KH (2)		Pescatori anal incontinence score (2)		Posttraumatic stress disorder symptom scale self report (2)		Patient health questionnaire -2/8/9/15 (9)	Health and social services utilisation questionnaire (1)		Awareness regarding newborn care questionnaire (1)				Sexual quality of life questionnaire (1)		Maternal postpartum quality of life tool (Portuguese) (2) [7]
	Pelvic floor symptom bother questionnaire (1)				Whitley index (2)		Hamilton rating scale for depression (7)	Current abuse score (1)		Maternal adjustment and attitude scale (1)				Sexual desire inventory (1)		Sexual function questionnaire medical impact scale (1)
	Female pelvic floor questionnaire (1)				Modified perinatal post traumatic stress disorder questionnaire (2)		Blues questionnaire (7)	Conflict tactics score (1)		Lederman postpartum self-evaluation questionnaire (1)				Vaginal changes sexual and body esteem (1)		Female sexual distress scale-revised (1)
	Prolapse and incontinence knowledge questionnaire (1)				Traumatic events scale (2)		Zung's self-rating depression scale (7)	Perinatal infant care social support scale (1)						Female sexual distress scale-revised (1)		Postpartum quality of life (RPQoL) (1) [9]
	Medical epidemiological and social aspects of ageing questionnaire (1)				Kellner symptoms questionnaire (2)		Postpartum depression screening scale (7)	Dyadic adjustment scale (1)						Sexual function questionnaire medical impact scale (1)		WHO disability assessment scale (1)
	Sandvik UI severity score (1)				Maudsley obsessive compulsive inventory (2)		Montgomery -Asberg depression rating scale (5)	Family adaptability and cohesion evaluation scales II (1)								Mother-generated index (1) [respondent driven]¥
	Ubersax incontinence impact questionnaire (1)				City trauma birth scale (Hebrew) (2)		Stein's maternity blues scale (Dutch) (4)	Maternal support scale (1)								Quality of life inventory (1)
	Overactive bladder questionnaire (1)				Schilling-sifneos personality scale (2)		Chinese health questionnaire (4)	Brown's support behavior inventory (1)								Swedish health related quality of life (1)
	Incontinence specific QoL questionnaire (1)				Karolinska scale of personality (2)		WHO self reporting questionnaire (3)	Marital adjustment score (1)								PSC (1) [6]
					Sense of coherence scale (2)		Postpartum depression predictors inventory (3)	Smilkstein's social support scale (1)								

© 2020 Sultan P et al. *JAMA Network Open.*

					Primary health questionnaire PHQ-15 (2)		Postnatal negative thoughts questionnaire (2)	Norbeck social support questionnaire (1)								
					Life event scale (2)		Taiwanese depression scale (1)	Interpersonal support evaluation list (1)								
					Yale-Brown obsessive compulsive scale (1)		Leverton questionnaire (1)	Partner support scale (1)								
					Zuckerman’s sensation seeking scale (1)		Dhoop chaon (1)	Social adjustment scale (1)								
					Demoralisation scale (1)		Punjabi postnatal depression score (1)	Social support questionnaire (1)								
					Neo five factor inventory (1)		General help seeking questionnaire (1)	Composite abuse scale (1)								
					Crockenberg stress checklist (1)		WHO-5 well-being index (1)	Index of marital satisfaction (1)								
					Maternal self-esteem questionnaire (1)			Relationship assessment scale (1)								
					Launay-Slade hallucination scale –revised (1)			Situation specific support scale (1)								
					Emotional processing scale (1)											
					Somatic symptom checklist 8 (1)											
					Unconditional self-acceptance questionnaire (1)											
					Jackson’s personality research form (1)											
					California personality inventory subscale (1)											
					Eysenck’s personality questionnaire (1)											
					Peters delusions inventory (1)											
					Maternal self report inventory (1)											

© 2020 Sultan P et al. *JAMA Network Open.*

					Self analysis questionnaire (1)											
					COPE inventory (1)											
					Highs scale (1)											
					Minnesota multiphasic personality inventory (1)											
					Affects balance scale (1)											
					Baby related and motherhood negative thoughts (1)											
					Body image self-conscious scale (1)											
					Coopersmith's self-esteem inventory (1)											
					Differential emotions scale (1)											
					Frost multidimensional perfectionism scale (1)											
					Kessler's psychological distress scale (1)											
					Modified PTSD symptom scale self-report (1)											
					Post traumatic diagnostic scale (Turkish) (1)											
					Posttraumatic stress diagnostic scale (1)											
					Psychological well-being scale (1)											

No PROMs assessing cognition domain among included outpatient studies; Many studies used several PROMs; Numbers below domain titles (X & Y): X signifies number of PROMs reported within domains and Y signifies the total number of studies using a PROM within this domain. For individual PROMs numbers in parentheses signify number of studies utilizing this PROM. Where relevant different languages of PROM are also stated in parentheses if stated in included study; For global recovery PROMs, number of domains covered by *obstetric specific* PROMs presented as [number]; ¥ mother generated index is a respondent driven PROM and therefore does not have a consistent number of domains; *Contains 3 domains about satisfaction and remaining domains about relationship between partners and infant care and social support. EPDS=Edinburgh postnatal depression score; SF-36=Short Form (36) Health Survey; FSFI=Female Sexual Function Index; STAI=State-Trait Anxiety Inventory; IES(-R)=Impact of Event Scale (-Revised); IFSAC=Inventory of Functional Status After Childbirth; ICIQ-SF=International

© 2020 Sultan P et al. *JAMA Network Open.*

Consultation on Incontinence Questionnaire – urinary incontinence -short form; UDI-6=Urinary Distress Inventory, short form; CES-D=Center for Epidemiological Studies Depression scale; IIQ-7=Incontinence Impact Questionnaire, short form; PISQ-12= Pelvic organ prolapse/urinary Incontinence Sexual Questionnaire; SF-12=12-item Short Form Health Survey; PTSD=Post Traumatic Stress Disorder; COPE; WHO=world health organization; QoL=quality of life; UI=urinary incontinence; ICIQ-KH= International Consultation on Incontinence Questionnaire (king's Health); OAB=overactive bladder; VS=vaginal symptoms; FLUTSsex= Female Sexual Matters Associated with Lower Urinary Tract Symptoms; KATZ-ADL= KATZ index of independence in activities of daily living; WHOQOL-BREF; WHO quality of life instrument; OWHSQ, Oxfordshire Women's Health Study questionnaire

© 2020 Sultan P et al. *JAMA Network Open.*

eTable 3. Summary of 73 Patient-Reported Outcome Measures (PROMs) Used to Evaluate Inpatient Recovery Following Childbirth

Physical function	Pain	Psychosocial distress			Psychosocial support	Sleep	Motherhood experience		Breast feeding / breast health	Fatigue	Sexual function	Cognition	Global recovery
(2 & 2)	(2 & 9)	Psychological (19 & 25)	Anxiety (3 & 15)	Depression (11 & 58)	(5 & 6)	(1 & 1)	Adapting to maternal role (2 & 2)	Maternal-neonatal bonding (5 & 6)	(2 & 3)	(5 & 6)	(1 & 1)	(3 & 3)	(13 & 23) [No. Domains]
*Rhodes index of nausea and vomiting (1)	McGill pain (short form, French) (8)	Impact of event scale (revised) (3)	STAI (12)	EPDS (36)	Berlin social support scale (2)	*Athens Insomnia scale (1)	*Parenting needs and parenting confidence questionnaire (1)	Postpartum bonding questionnaire (2)	Breastfeeding self-efficacy scale (BSES / SF) (2)	*Fatigue continuum form (2)	FSFI (Taiwan) (1)	*Attentional function index (1)	SF-36 (8)
KATZ-ADL (1)	Pain catastrophizing scale (1)	Big five inventory (2)	Zung's self-rating anxiety scale (1)	Beck depression inventory I/II (5)	*Four factor index of social status (1)		Parenting sense of competence scale (1)	*Intimate bonds measure (1)	*Infant breastfeeding assessment tool (1)	Multidimensional fatigue inventory (1)		*Wechsler logical memory test (1)	EuroQoL 5 (2)
		Highs scale (Japanese) (2)		Blues questionnaire (3)	Karolinska scales of personality (1)			*Maternal attachment inventory (1)		Modified fatigue symptom checklist (1)		*Wechsler visual reproduction test (1)	Inventory of functional status after childbirth (2) [5]
		Neo Five factor inventory (2)		CES-D (Taiwan) (2)	*Mieczyslaw Plopa and Jan Rostowski marriage questionnaire (1)			*Mother infant bonding scale (1)		*Multidimensional fatigue scale (1)			*ObsQoR-11 (2) [4]
		*Peritraumatic dissociative experience questionnaire (2)	General health questionnaire (2)		Social support questionnaire (1)			*Parental bonding instrument (1)		*Postpartum fatigue scale (1)			Nottingham health profile (1)
		*Life orientation test (1)		*Kennerley and gath score (2)									*Parents' postnatal sense of security Swedish instrument (1) [5]
		*General self-efficacy scale (1)		Patient health questionnaire 9/15 (2)									*Postpartum comfort questionnaire (1) [4]
		*Peritraumatic emotions list (1)		Stein's maternity blues scale (2)									*QoR-15 (1)
		*Post-discharge		Zung's self-									*QoR-40 (1)

© 2020 Sultan P et al. *JAMA Network Open.*

		coping difficulty scale (1)		rating depression scale (2)									
		*Trauma history questionnaire (1)		*Daily experiences questionnaire (1)									*Readiness for hospital discharge scale (1)
		*Vulnerable personality scale self-report instrument (1)		Montgomery-Asberg depression rating scale (1)									Barkin index of maternal functioning (1) [5]
		Maudsley obsessive compulsive inventory (1)											PSC (1) [6]
		Perceived stress scale (1)											*RCSS (1) [5]
		Perinatal PTSD questionnaire (1)											
		Maternal self report inventory (1)											
		Profile of mood states (1)											
		Schilling-sifneos personality scale (1)											
		Sense of coherence scale (1)											
		Whitley index (1)											

* = 32 unique inpatient recovery PROMs (PROMs not included in any outpatient studies / Table 2). For global recovery PROMs, number of domains covered by obstetric specific PROMs presented as [number]; No PROMs assessed “Surgical complications” or “Scar and wound healing” domains as part of included inpatient recovery studies. ObsQoR-11=Obstetric Quality of recovery -11 scoring tool; PSC=postpartum symptom checklist; RCSS= Recovery from Caesarean Section Scale; QoR-15=Quality of Recovery -15 scoring tool; QoR-40=Quality of Recovery-40 scoring tool

© 2020 Sultan P et al. *JAMA Network Open.*
